# Zeb2 Controls Retinal Physiological and Pathological Angiogenesis by Regulating Astrocyte Proliferation and Differentiation

**DOI:** 10.1111/cpr.70236

**Published:** 2026-05-26

**Authors:** Jing Liu, Yanan Guo, Kangjian Xiang, Shuyi Wang, Dongchang Xiao, Mengqing Xiang

**Affiliations:** ^1^ State Key Laboratory of Ophthalmology, Guangdong Provincial Key Laboratory of Ophthalmology and Visual Science, Zhongshan Ophthalmic Center, Sun Yat‐sen University Guangzhou China; ^2^ Guangdong Provincial Key Laboratory of Brain Function and Disease, Zhongshan School of Medicine, Sun Yat‐sen University Guangzhou China

**Keywords:** neurotoxic astrocyte, oxygen‐induced retinopathy, proliferation, retinal astrocyte, retinopathy of prematurity, Zeb2

## Abstract

Retinal angiogenesis relies on a precisely timed interaction between astrocytes and endothelial cells (ECs), yet the transcriptional regulatory program underlying this complex neurovascular crosstalk remains poorly characterized. Here, we define Zeb2 as a pivotal transcriptional modulator of retinal astrocyte function that coordinates developmental and pathological vascular growth. It is transiently expressed in retinal astrocyte progenitor cells during development and re‐induced under pathological hypoxia. Conditional ablation of *Zeb2* in retinal astrocytes enhanced their proliferation, migration, and maturation by upregulating VEGFA and altering other signalling, leading to excessive superficial vascular growth during development. In oxygen‐induced retinopathy, *Zeb2* inactivation exacerbated pathological neovascularization while impairing reparative revascularization, which is associated with a transcriptional signature favouring tuft ECs over tip ECs. Mechanistically, it inhibited the neurotoxic A1 astrocyte identity, resulting in dampened inflammatory response and diminished genetic program promoting revascularization and/or preventing neovascularization including Plxnd1, Nrf2, and FGF2 signalling. These findings establish Zeb2 as an oxygen‐sensitive regulator of astrocyte function that differentially modulates physiological and pathological angiogenesis, highlighting its potential as a therapeutic target in proliferative retinopathies.

## Graphical abstract

During development, the oxygen‐sensitive transcription factor Zeb2 restrains astrocyte proliferation and maturation to ensure balanced retinal angiogenesis. In disease, it promotes the neurotoxic A1 astrocyte phenotype and inflammation, thereby promoting reparative revascularization over pathological neovascularization.

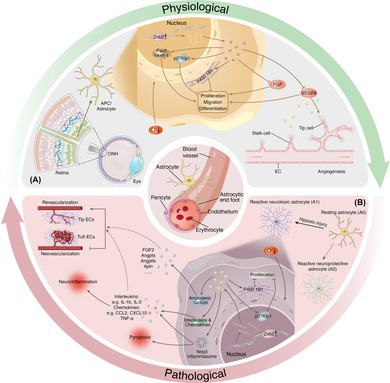

## 1 Introduction

Astrocytes are an essential component of the central nervous system (CNS), serving critical functions in both supporting and compartmentalizing nerve cells and participating in the formation of the blood–brain barrier [1]. Recent years have seen a growing interest by researchers in the direct effects of astrocytes on non‐neuronal cells, especially endothelial cells (ECs). Previous studies have explored the important guidance role of astrocytes during retinal vascular development and pathological processes. Due to the unique structure of retinal blood vessels, the retina serves as an important model for studying the mechanism of mutual regulation between astrocytes and blood vessels, both in development and pathological conditions. During early ocular vascular development, astrocytes are involved as a major player in regulating angiogenesis in the retinal surface layer by providing a physical meshwork and pro‐angiogenic factors. Also, substances secreted by ECs and oxygen that enters after vascular formation, in turn, may alter the characteristics of astrocytes [2, 3]. On the other hand, astrocyte dysfunction can lead to impaired retinal vascularization, disruption of the blood‐retinal barrier (BRB), and reactive gliosis, resulting in a range of blinding disorders, such as proliferative retinopathies (e.g., retinopathy of prematurity, diabetic retinopathy), corneal defects, and glaucoma [4, 5, 6, 7]. Therefore, understanding the precise regulation of angiogenesis by astrocytes is of great significance for the clinical treatment of vascular‐related diseases.

Various cell adhesion molecules, such as integrins, cadherins, and laminin protein complexes from astrocytes, serve as scaffolds for angiogenesis. Pro‐ and anti‐angiogenic factors are also released by astrocytes. For instance, the synthesis and release of VEGF, FGF2, and angiopoietins by astrocytes may be essential for the formation and growth of the vascular bed in the brain and retina [8, 9, 10]. In particular, depending on the level of activation of the HIF/VEGF system and the developmental context, the elimination of hypoxia inducible factor‐2α (HIF2a) or VEGF production from astrocytes both inhibits radial migration of the retinal ECs and angiogenesis [11, 12]. Moreover, injury‐induced reactive astrocyte proliferation occurs with changes in the morphology and function of the astrocytes [13, 14]. It is plausible that such changes may cause vascular defects. In fact, it has been suggested that overproduction of astrocytes can delay angiogenesis and alter vascular patterning in the pathogenesis of retinopathy of prematurity [15, 16]. Yet, despite recent advances, many aspects of the astrocyte biology during angiogenesis still remain poorly understood.

The zinc finger E‐box binding homeobox 2 (Zeb2) transcription factor, also known as SIP1, is a key regulator of cell fate determination, differentiation, and epithelial‐to‐mesenchymal transition in various developmental contexts [17]. Mutations in *ZEB2* cause the Mowat‐Wilson syndrome in humans, which is associated with neurodevelopmental defects and ocular abnormalities [17, 18], highlighting its importance in neural development. Previous studies have established critical roles for Zeb2 in the nervous system, such as corticogenesis, myelination in the CNS, Schwann cell differentiation, and the regulation of reactive astrogliosis and functional recovery following CNS injury [19, 20, 21, 22]. While we and others have previously demonstrated an essential function of Zeb2 in the specification and maintenance of non‐photoreceptor cells during retinal development [23, 24], its expression pattern and functional significance in retinal astrocytes remain unexplored.

In this study, we set out to determine the spatiotemporal expression pattern of Zeb2 during retinal astrogliogenesis, and to investigate its specific function in retinal astrocytes by generating astrocyte‐specific conditional knockout mice. We further examined the impact of astrocytic *Zeb2* ablation on vascular development and, using the oxygen‐induced retinopathy (OIR) model, on the pathological responses to hypoxic injury. Our findings reveal that Zeb2 is oxygen‐sensitive and transiently expressed in retinal astrocyte progenitor cells (APCs), and its astrocytic ablation promotes astrocyte proliferation, maturation, and migration, thereby reshaping the astrocytic template network during development. This, in turn, alters vascular growth and branching during developmental angiogenesis. Paradoxically, in the pathological context of OIR, Zeb2 promotes a neurotoxic A1 astrocyte response that, while inflammatory, is necessary to prevent exacerbated neovascularization and support physiological revascularization. This work establishes astrocytic Zeb2 as a central coordinator of the neurovascular unit both in health and disease.

## 2 Results

### 2.1 Transient Expression of Zeb2 in Retinal APCs and Differentiating Astrocytes

To determine whether Zeb2 is expressed in retinal astrocytes during development, we detected Zeb2 expression by immunofluorescence staining of flat‐mount retinas from developing wild‐type mice. At E15.5 when APCs begin to colonize the retina [25, 26], all APCs surrounding the optic nerve head (ONH) were immunoreactive for Pax2, a nuclear protein marker for APCs and mature astrocytes, but were negative for Zeb2 (Figure S1). However, 2 days later, most of the Pax2‐marked APCs became immunoreactive for Zeb2 at E17.5, and Zeb2 remained to be seen in the differentiating astrocytes migrating away from the ONH (Figure S1). By P0, however, most emerging APCs lost Zeb2 expression (Figure S1). Therefore, Zeb2 appears to be transiently expressed in retinal APCs and differentiating astrocytes during embryonic development, implicating an early role in retinal astrocyte development. Blood vessels are known to enter the retina through the ONH around P0 [9, 26], changing ONH to an oxygenated state from a state of physiological hypoxia in the embryonic retina. Interestingly, Zeb2 downregulation in APCs surrounding the ONH is coincident with the timing of ONH oxygenation.

### 2.2 Astrocyte‐Specific *Zeb2* Ablation Enhances Proliferation, Migration and Maturation of Retinal Astrocytes

**FIGURE 1:**
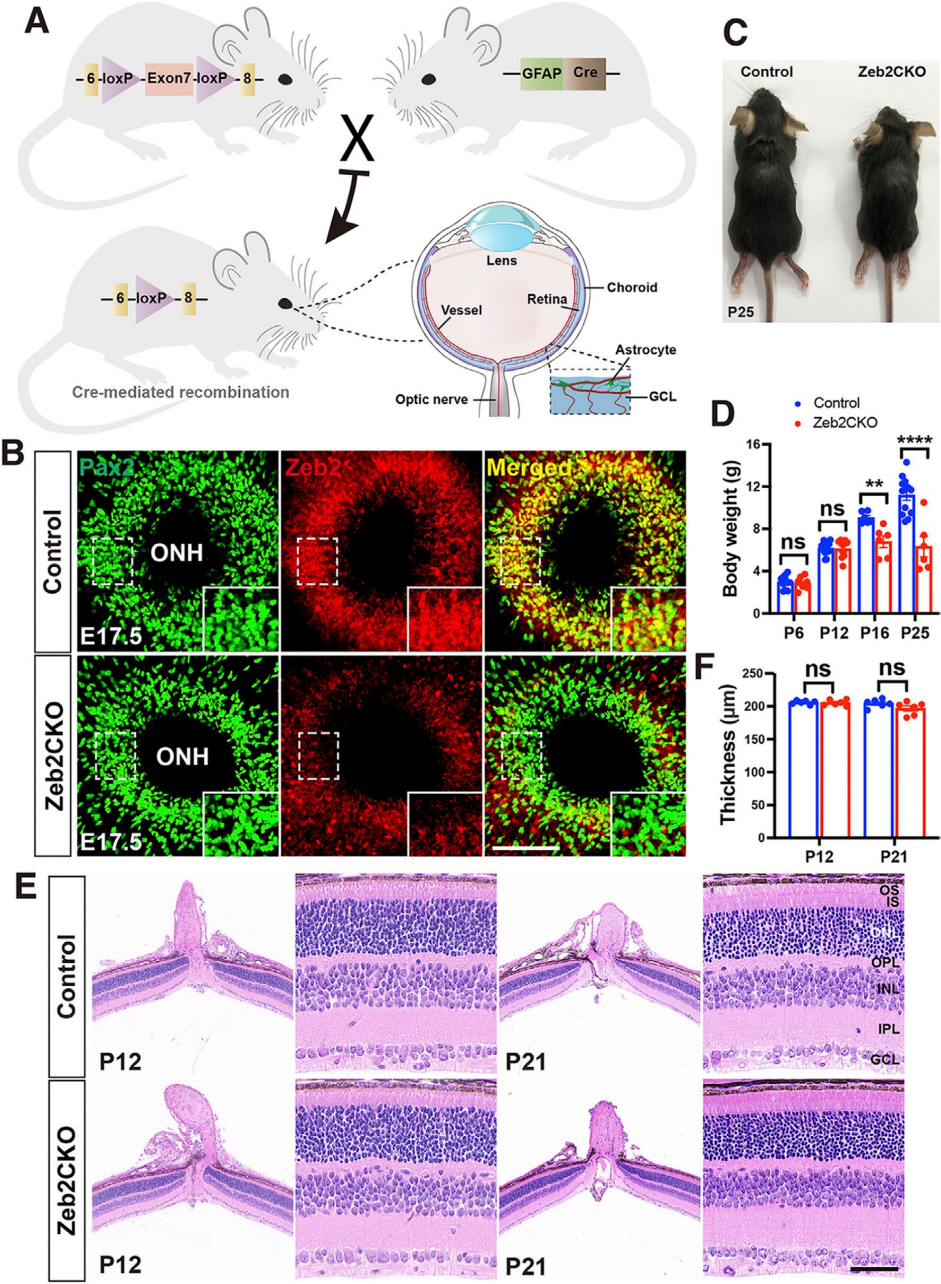
Astrocytic *Zeb2* ablation impairs postnatal mouse development but not retinal structure. (A) Schematic representation of the GFAP‐Cre transgene and Cre‐mediated recombination of the floxed *Zeb2* allele, which results in deletion of exon 7 of *Zeb2* in developing astrocytes of the Zeb2CKO (Zeb2^fl/fl^; GFAP‐Cre) mice. Depicted also is a cross‐sectional eyeball structure with the retinal vascular and astrocytic networks. **(B)** E17.5 flat‐mount retinas were double‐immunolabelled with an anti‐Zeb2 antibody (red) and an anti‐Pax2 antibody (green) to validate *Zeb2* ablation in Zeb2CKO retinal astrocytes. (C) The body size of Zeb2CKO animals is significantly diminished at P25. (D) Quantification of body weight of control and Zeb2CKO mice at different postnatal stages. Data are presented as mean ± SEM (*n* = 6–16). **p < 0.01, ****p < 0.0001. (E) Retinal sections from P12 and P21 control and Zeb2CKO animals were stained with HE (haematoxylin–eosin). (F) Quantification of the thickness of control and Zeb2CKO retinas at P12 and P21. Data are presented as mean ± SEM (*n* = 6). ns, no significance. Abbreviations: GCL, ganglion cell layer; INL, inner nuclear layer; IPL, inner plexiform layer; IS, inner segment; ONH, optic nerve head; ONL, outer nuclear layer; OPL, outer plexiform layer; OS, outer segment. Scale bar: B, 100 μm; E, 50 μm.

To investigate the potential role of Zeb2 during the development of retinal astrocytes, we conditionally inactivated *Zeb2* in retinal astrocytes and their progenitors using a floxed *Zeb2* allele and the GFAP‐Cre driver mouse line [12, 27], which is approximately 99% efficient in Cre‐mediated recombination in neonatal retinal astrocytes [12] (Figures 1A and S2A). In conditional knockout (Zeb2^fl/fl^; GFAP‐Cre) mice (hereafter referred to as Zeb2CKO), only residual Zeb2‐positive retinal APCs remained adjacent to the ONH at E17.5, demonstrating the efficiency and specificity of *Zeb2* inactivation (Figure 1B).

The Zeb2CKO animals were born at a normal Mendelian ratio; however, they exhibited abnormal walking and epileptic behaviours at 12 days after birth (P12), and then died around P25, consistent with the previously reported phenotype [28]. In addition, Zeb2CKO mice showed delayed physical development, with a significant decrease in body weight after P16 (Figure 1C,D). Despite the reduced body size of Zeb2CKO mice, the sizes of their eyeballs and retinas were essentially the same as those of the control group (Figure S2B,C). We further performed HE (haematoxylin–eosin) staining of retinal sections, which did not reveal any difference in retinal structure and thickness between the two groups (Figure 1E,F). These results suggest that astrocytic Zeb2 is strictly required for overall mouse development and survival, but may be largely dispensable for retinal development.

**FIGURE 2:**
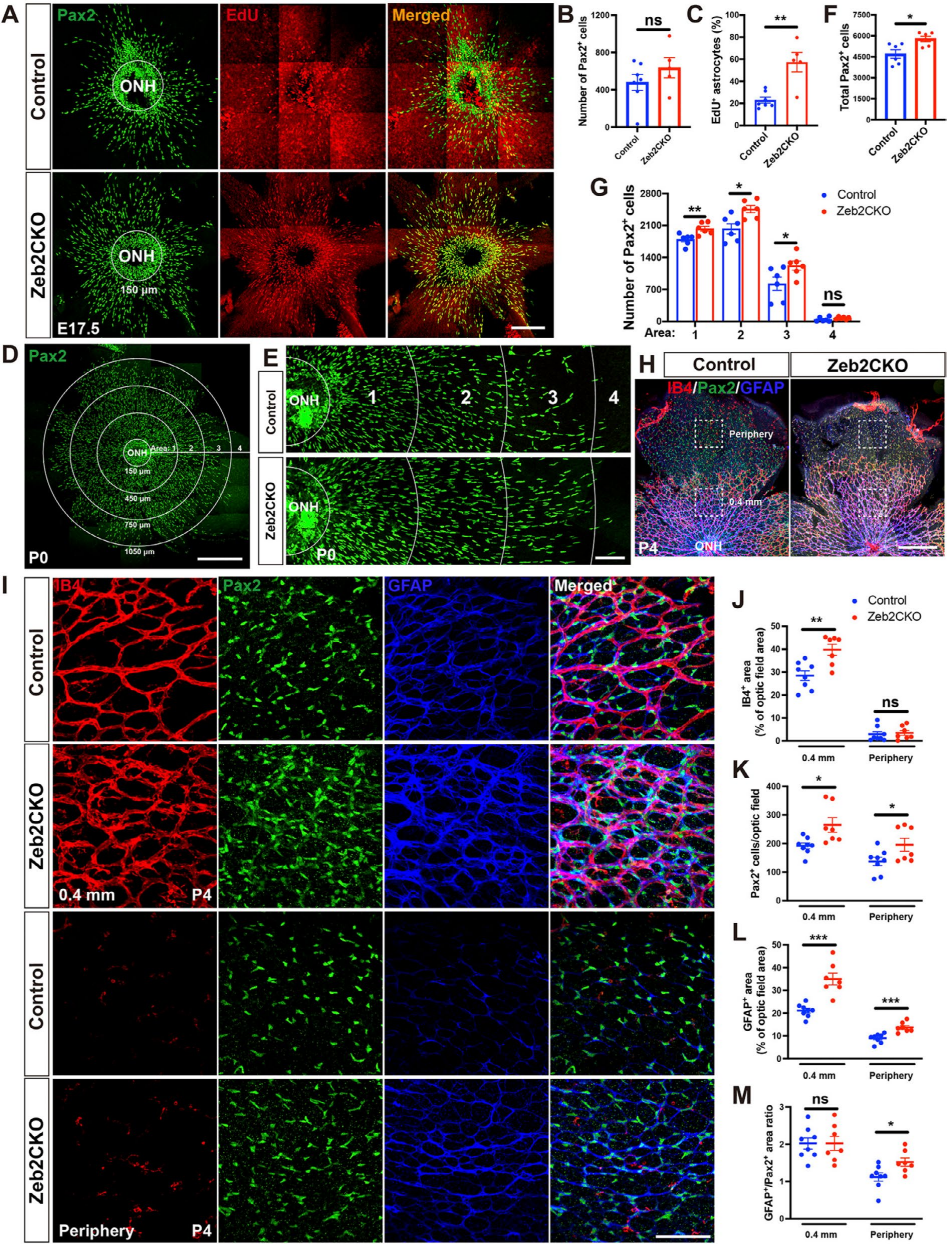
Astrocytic *Zeb2* ablation strengthens the proliferation, migration and maturation of retinal astrocytes. (A) Flat‐mount retinas from E17.5 control and Zeb2CKO embryos were co‐labelled for Pax2 (green) and EdU (red). (B) Quantification of all Pax2^+^ cells in the region beyond 150 μm from the ONH (denoted in A) in E17.5 control and Zeb2CKO retinas. Data are presented as mean ± SEM (*n* = 5–7). ns, no significance. (C) Percentage of proliferative astrocytes (EdU^+^Pax2^+^/Pax2^+^) in the region beyond 150 μm from the ONH in E17.5 control and Zeb2CKO retinas. Data are presented as mean ± SEM (*n* = 5–7). **p < 0.01. (D) Representative confocal image of Pax2 immunofluorescence of a P0 flat‐mount retina divided into four circular regions every 300 μm starting from 150 μm from the ONH. (E) Representative higher magnification images of Pax2 immunofluorescence from the four circular areas in P0 control and Zeb2CKO retinas. (F) Quantification of the total Pax2^+^ nuclei from all four circular areas in P0 control and Zeb2CKO retinas. Data are presented as mean ± SEM (*n* = 6). *p < 0.05. (G) Quantification of the Pax2^+^ nuclei from each of the four circular areas in P0 control and Zeb2CKO retinas. Data are presented as mean ± SEM (*n* = 6). *p < 0.05, **p < 0.01; ns, no significance. (H) P4 control and Zeb2CKO flat‐mount retinas were triple‐immunostained for IB4, Pax2 and GFAP. (I) Higher‐magnification and single‐channel images of the two outlined regions labelled as 0.4 mm (from the ONH) or Periphery in (H). (J–M) Quantification of IB4^+^ area (J), Pax2^+^ cells (K), GFAP^+^ area (L), and GFAP^+^/Pax2^+^ area ratio (M) from the two magnified regions in P4 control and Zeb2CKO retinas. Data are presented as mean ± SEM (*n* = 7 or 8). *p < 0.05, **p < 0.01, ***p < 0.001. ns, no significance. Abbreviations: IB4, isolectin B4; ONH, optic nerve head. Scale bar: D, H, 500 μm; A, 200 μm; E, I, 100 μm.

Murine retinal astrocytes are born as APCs in the optic stalk and migrate from the ONH to the surface of the retina from about E16 onward, moving in a radial pattern toward the retinal margin [25, 26]. To investigate the effect of astrocytic *Zeb2* inactivation on APCs, we pulse‐labelled E17.5 retinas by EdU (5‐ethynyl‐2′‐deoxyuridine), followed by simultaneous immunostaining for the APC marker Pax2 and EdU labeling to allow for analysis of both the total and proliferative APCs (Figure 2A). In the Zeb2CKO retina, the total number of Pax2^+^ APCs displayed an upward trend compared to the control, without reaching statistical difference (Figure 2A,B). Nevertheless, the number of EdU^+^ APCs increased by approximately 1.5‐fold in the Zeb2CKO retina (Figure 2A,C), indicating a dramatic enhancement in APC proliferation. At P0 when ECs start to enter the retina from the ONH, we compared, between the control and Zeb2CKO retinas, the total number of Pax2^+^ astrocytes and the number of astrocytes in the 4 circular areas shown in Figure 2D. We observed a 22.9% increase in the total number of Pax2^+^ astrocytes in the Zeb2CKO retina compared to the control (Figure 2E,F). Moreover, this difference appeared to centrifugally increase from the central to the peripheral retina, as we detected a 13.3%, 21.3%, and 48.4% increase in the number of astrocytes in areas 1, 2, and 3 of the Zeb2CKO retina, respectively (Figure 2E,G), suggesting astrocyte migration is hastened in the Zeb2CKO retina. Thus, astrocytic ablation of *Zeb2* appears to promote retinal astrocyte proliferation and migration during development.

To investigate astrocyte maturation, we triple‐stained P4 control and Zeb2CKO flat‐mount retinas for isolectin B4 (IB4), Pax2 and GFAP to specifically label blood vessels, astrocytic nuclei and mature astrocytes, respectively. We found that at a distance of 0.4 mm from the ONH, where astrocytes have already matured, there were more Pax2^+^ astrocytes and a denser GFAP^+^ astrocytic network in the Zeb2CKO retina (Figure 2H,I,K,L). However, the GFAP^+^/Pax2^+^ area ratio, an indicator of astrocyte maturation [15], exhibited no significant change between the two groups in this region (Figure 2M). In the periphery of the control retina, we observed numerous Pax2^+^ astrocyte nuclei as well, but only weak GFAP^+^ signals and a low value of GFAP^+^/Pax2^+^ area ratio (Figure 2H,I,K–M), indicating that the degree of astrocyte maturation in the control peripheral retina is very low at P4. In contrast, we observed a substantial increase in Pax2^+^ astrocytes, GFAP^+^ signals and GFAP^+^/Pax2^+^ area ratio in the periphery of the Zeb2CKO retina (Figure 2I,K–M), suggesting that astrocytic *Zeb2* inactivation results in enhanced astrocyte maturation. By P12, however, the observed enhancement effect of *Zeb2* ablation on astrocyte number and maturation disappeared (Figure S3A–D). Overall, these results together suggest that astrocytic ablation of *Zeb2* leads to enhanced astrocyte proliferation, migration and maturation during retinal development.

### 2.3 Astrocytic *Zeb2* Ablation Leads to Excessive Growth of Retinal Superficial Vessels

**FIGURE 3:**
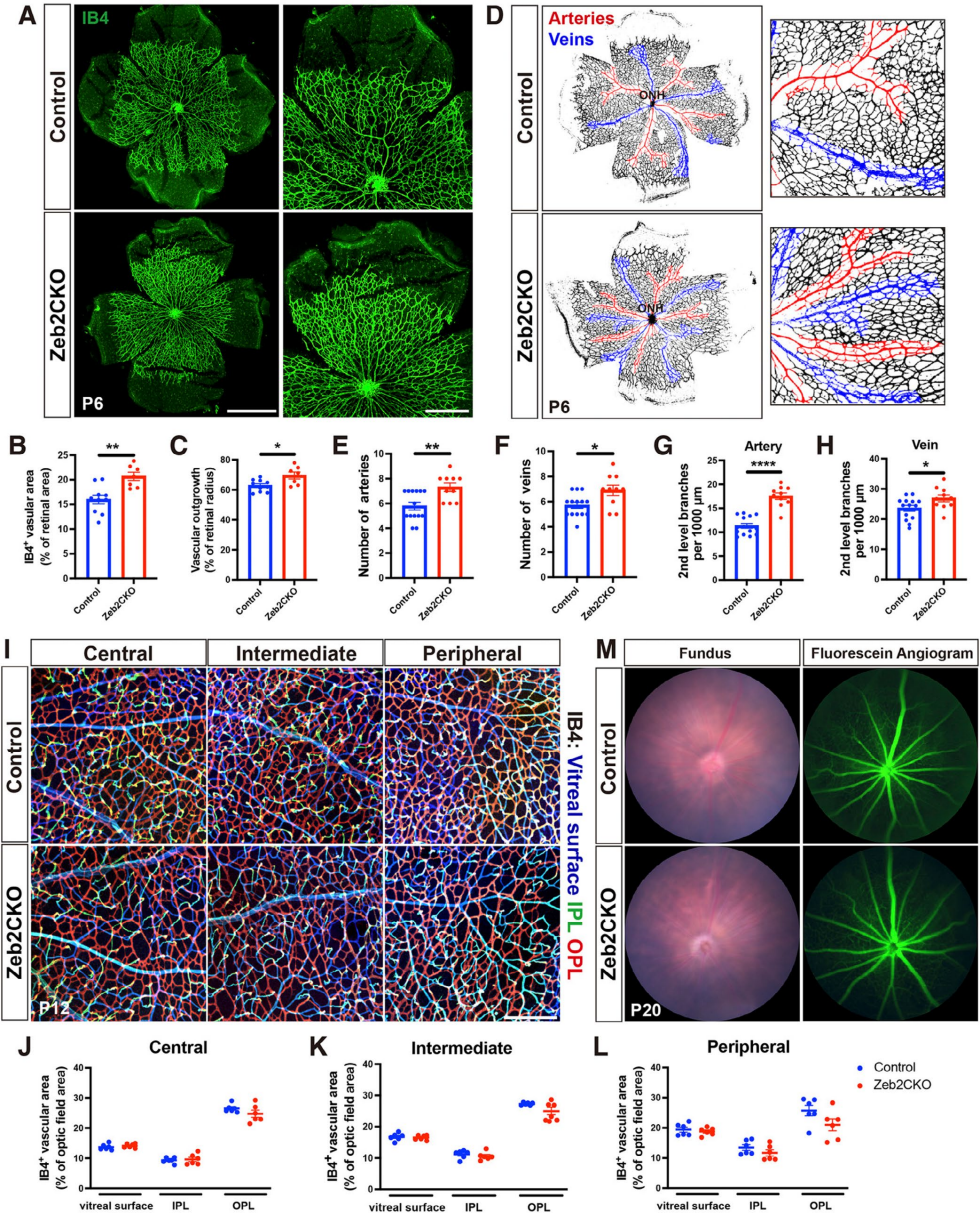
Astrocytic *Zeb2* ablation alters early vascular morphogenesis in the retina. (A) Representative overview images of IB4 immunofluorescence (left) and corresponding higher‐magnification images (right) of P6 control and Zeb2CKO flat‐mount retinas for visualizing vascular development of the superficial vascular plexus. (B) Quantification of IB4^+^ vascular area in P6 control and Zeb2CKO retinas. Data are presented as mean ± SEM (*n* = 7–10). **p < 0.01. (C) Quantification of vascular outgrowth in P6 control and Zeb2CKO retinas. Data are presented as mean ± SEM (*n* = 7–10). *p < 0.05. (D) Representative cartoon diagrams of arteries (red) and veins (blue) in the flat‐mount vasculature of P6 control and Zeb2CKO retinas. (E) Quantification of the number of arteries in P6 control and Zeb2CKO retinas. Data are presented as mean ± SEM (*n* = 10–14). **p < 0.01. (F) Quantification of the number of veins in P6 control and Zeb2CKO retinas. Data are presented as mean ± SEM (*n* = 10–14). *p < 0.05. (G) Quantification of 2nd level branches of arterial blood vessels starting from 1000 μm beyond the ONH in P6 control and Zeb2CKO retinas. Data are presented as mean ± SEM (*n* = 10–14). ****p < 0.0001. (H) Quantification of 2nd level branches of venous blood vessels starting from 1000 μm beyond the ONH in P6 control and Zeb2CKO retinas. Data are presented as mean ± SEM (*n* = 10–14). *p < 0.05. (I) IB4 immunofluorescence (vitreal surface vasculature, blue; IPL vasculature, green; OPL vasculature, red) images of P12 control and Zeb2CKO flat‐mount retinas in the central, intermediate and peripheral regions. (J–L) Quantification of IB4^+^ vascular area of P12 control and Zeb2CKO retinas in the central (J), intermediate (K) and peripheral (L) regions. Data are presented as mean ± SEM (*n* = 6). (M) Fundus photographs (left) and corresponding fluorescein angiograms of P20 control and Zeb2CKO retinas. Abbreviations: IPL, inner plexiform layer; ONH, optic nerve head; OPL, outer plexiform layer. Scale bar: A, 1000 μm (left), 500 μm (right); I, 200 μm.

Astrocytes are known to regulate retinal angiogenesis through the astrocytic arbor network as a template and providing molecular cues to guide the growth of ECs [25, 26]. We therefore investigated whether the astrocytic developmental defects caused by the conditional *Zeb2* ablation would subsequently affect retinal angiogenesis. By comparing and quantifying ECs immunoreactive for the nuclear ERG (ETS‐related gene) protein marker at P0, we observed a significant elevation in the number of ECs that entered the retina in Zeb2CKO vs. control animals (Figure S4). Similarly, compared to controls, the density of IB4‐positive vasculature at the 0.4 mm region (from the ONH) was higher in P4 Zeb2CKO retinas (Figure 2I,J). The increased ECs or vascular density was consistent with the elevated Pax2^+^ astrocytes in Zeb2CKO retinas at these two stages (Figure 2E–G,I–K). At P6, the retinal vasculature of control animals is still actively sprouting and the more proximal region is remodelling into a hierarchical network with arteries, arterioles, venules, and veins (Figure 3A). In contrast, a denser vascular plexus was formed in P6 Zeb2CKO retinas with larger vascular area and increased radial vessel outgrowth (Figure 3A–C). There was also a notable increase in the total number of arteries and veins in Zeb2CKO retinas (Figure 3D–F). Moreover, when comparing branches of individual arteries and veins, we observed a significant increase in the 2nd level branches of arteries and veins in Zeb2CKO retinas (Figure 3D,G,H), suggesting that astrocytic *Zeb2* inactivation may cause a microvascular abnormality resulting from defective remodelling of the superficial vascular plexus.

At later stages of retinal angiogenesis, we analysed and compared all three‐layer vascular plexuses on the vitreal surface and within the inner plexiform and outer plexiform layers of the control and Zeb2CKO retinas. At P12, compared to control retinas, Zeb2CKO retinas exhibited similar vascular densities in all three plexus layers in the central, intermediate, and peripheral regions (Figure 3I–L), suggesting a recovery from the earlier vascular overproduction phenotype in P12 Zeb2CKO retinas. This vascular recovery was in line with the normal number and maturation of astrocytes determined by Pax2 and GFAP immunolabelling in P12 Zeb2CKO retinas (Fig. S3A‐D). Further, similar vasculature results were obtained from P16 control and Zeb2CKO retinas (Figure S3E–H), implicating a critical role for astrocytic Zeb2 in early rather than late retinal angiogenesis.

We next performed intravital imaging of the retinal vasculature in control and Zeb2CKO animals using fluorescein angiography (FFA) to determine the BRB intactness. In P20 Zeb2CKO retinas, we observed no fluorescence signal dispersion or significant alteration in the vasculature (Figure 3M), indicating astrocytic *Zeb2* inactivation has no effect on the BRB. Together, our results thus suggest a crucial role for Zeb2 in early angiogenesis of the retinal superficial layer via regulation of astrocytic proliferation, differentiation, and migration.

### 2.4 Astrocytic *Zeb2* Ablation Causes Expression Alteration of Angiogenic and Astrogliogenic Genes

To gain molecular insights into the astrocytic and angiogenic phenotypes caused by the conditional *Zeb2* inactivation, we performed RNA‐seq analysis to identify differentially expressed genes (DEGs) in the Zeb2CKO retina. RNA was extracted from control and Zeb2CKO retinas at E17.5 when Zeb2 is strongly expressed in APCs. This analysis identified 159 DEGs, most of which are upregulated in the mutant retina (Figure S5A–D; Table S1). GO (gene ontology) enrichment analysis and GSEA (gene set enrichment analysis) showed that many of the upregulated DEGs are enriched for angiogenesis‐related GO terms, including positive regulation of angiogenesis, vasculogenesis, endothelium development, and endothelial cell development; additionally, some are enriched for those associated with glial cell differentiation, FGF receptor signalling, or GDNF receptor signalling (Figure S5E–G). Consistent with these results, the expression heatmap shows in E17.5 Zeb2CKO retinas obvious upregulation of a variety of angiogenic genes such as *Sox18, Tie1, Tek*, and *Cd34*, as well as *Vegfa*, *Pax2*, and *Nkx6‐1* (Figure S5H), which have previously been implicated in astrocyte proliferation and differentiation [12, 29, 30, 31]. Moreover, *Cyp1b1* (P450 1B1) and *Cdkn1b* (p27Kip1) are known inhibitory genes of astrocyte proliferation [32, 33] and are downregulated in the Zeb2CKO retina (Figure S5H). qRT‐PCR assay further validated the expression changes of most of these DEGs in P0 Zeb2CKO retinas (Figure S5I), consistent with the enhanced angiogenesis and astrogliogenesis observed in Zeb2CKO retinas.

Despite the *Vegfa* upregulation, qRT‐PCR assay detected no alteration in the expression of *Hif1a*, *Epas1 (Hif2a), Angpt1, Angpt2, and Fgf2* in the P0 Zeb2CKO retina (Figure S6A). Consistent with the RNA expression levels, Western blot and/or immunolabelling analyses confirmed VEGFA upregulation but unchanged HIF2a protein expression in the mutant retina (Figure S6B–E), indicating that the observed *Vegfa* upregulation in Zeb2CKO retinas may be independent of HIF1a and HIF2a. To determine whether Zeb2 directly represses *Vegfa* expression, we performed chromatin immunoprecipitation (ChIP) assay using chromatin DNA prepared from adult mouse retinas. Within a 2.3‐kb region upstream of the transcription start site (TSS) of *Vegfa*, we identified 4 putative Zeb2 binding motifs [5′‐CACCT(G)‐3′] [34] (Figure S7A). We tested two of them and both were specifically enriched by an anti‐Zeb2 antibody, whereas there was no significant enrichment for two intron fragments containing no Zeb2 binding motifs (Figure S7A,B). We next subcloned a 2‐kb *Vegfa* promoter fragment into a luciferase reporter vector to obtain the *Vegfa* promoter reporter plasmid, which displayed substantial basal luciferase activity in 293T cells in the presence of control expression plasmid (Figure S7C,D). However, this activity was decreased by cotransfection with the Zeb2 expression plasmid in a dose‐dependent manner (Figure S7C,D). Therefore, Zeb2 appears to act as a transcriptional repressor of *Vegfa* by directly binding to its promoter.

To further understand how Zeb2 may control the expression of the identified DEGs that may contribute to astrocyte proliferation and development, we carried out CUT&Tag chromatin profiling of E17.5 mouse retinas, where Zeb2 is strongly expressed in APCs. Numerous Zeb2 binding peaks were identified which were weakly enriched for the CUT&Tag signals of H3K27me3, a histone mark associated with transcriptional gene repression (Figure S7E). Based on a de novo motif search, the Zeb2 peaks are enriched for the Zeb2 DNA binding motif (5′‐CACCTG‐3′) as expected (Figure S7F). Consistent with the ChIP assay result, Zeb2 exhibits several CUT&Tag peaks in the *Vegfa* promoter region (Figure S7G). In addition, Zeb2 occupies the promoter regions of *Cdkn1b, Cyp1b1* and *Nkx6‐1* (Figure S7G), but not *Pax2*, suggesting that Zeb2 may both directly and indirectly regulate downstream genes involved in astrocyte proliferation and differentiation.

### 2.5 Zeb2 Is Hypoxia‐Inducible and Promotes Pathological Astrocyte Activation

**FIGURE 4:**
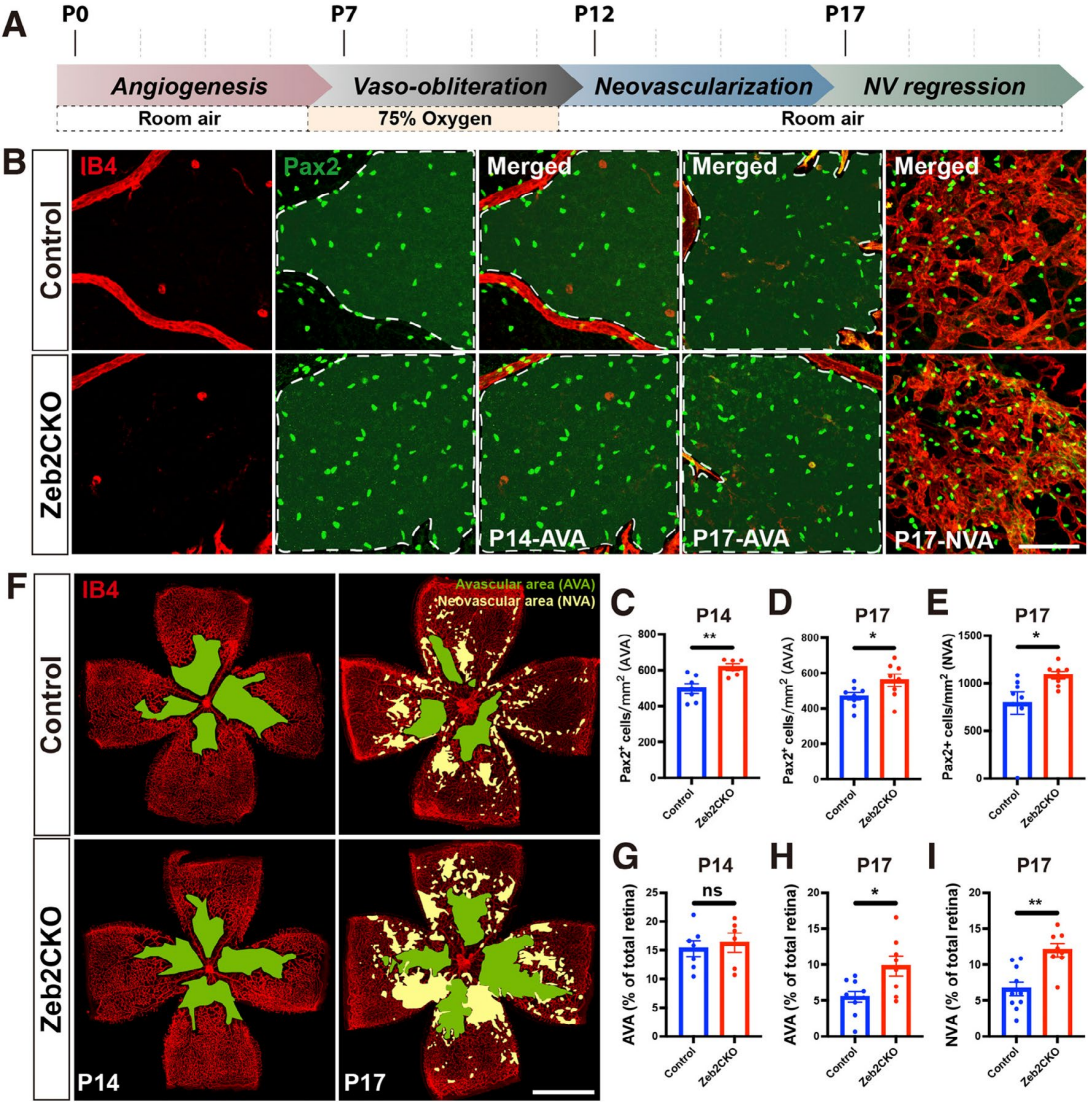
Pathological astrocyte activation and aggravated neovascularization and vaso‐obliteration in Zeb2CKO retinas of oxygen‐induced retinopathy (OIR) mice. (A) Experimental design for generating OIR mouse models. NV, neovascularization. (B) Representative confocal images of IB4 (red) and Pax2 (green) immunostaining of control and Zeb2CKO retinas from P14 and P17 OIR mice. The regions of avascular area (AVA) and neovascular area (NVA) are shown, and the AVA regions are outlined by dashed lines. (C) Quantification of Pax2^+^ cells in the AVA of P14 control and Zeb2CKO OIR retinas. Data are presented as mean ± SEM (*n* = 6 or 7). **p < 0.01. (D,E) Quantification of Pax2^+^ cells in the AVA or NVA of P17 control and Zeb2CKO OIR retinas. Data are presented as mean ± SEM (*n* = 8). *p < 0.05. (F) Representative confocal images of IB4 immunolabelling of P14 and P17 control and Zeb2CKO OIR retinas. The AVA and NVA are highlighted in green and yellow, respectively, on the flat‐mount retina. (G) Quantification of the AVA in P14 control and Zeb2CKO OIR retinas. Data are presented as mean ± SEM (*n* = 6 or 7). ns, no significance. (H, I) Quantification of the AVA or NVA in P17 control and Zeb2CKO OIR retinas. Data are presented as mean ± SEM (*n* = 8–10). *p < 0.05, **p < 0.01. Scale bar: B, 100 μm; F, 1 mm.

It has been shown that hypoxic injury leads to pathological activation of astrocytes, resulting in aberrant angiogenesis [15]. Given the developmental role of Zeb2 in astrocytic proliferation and differentiation, we speculated that Zeb2 might also be a driving force of astrocyte proliferation and activation in pathological conditions. To test this possibility, we generated OIR mouse models by first placing P7 control and Zeb2CKO mice under hyperoxic conditions (75% O_2_) for 5 days, which kept vaso‐obliteration to the superficial vascular plexus. We then returned the mice to normal oxygen conditions (21% O_2_), which elicited a strong hypoxic response and resulted in pathological neovascularization and tuft formation (Figure 4A,B). Both control and Zeb2CKO retinas displayed stereotypical patterns of avascular areas (AVAs) and neovascular areas (NVAs) with clustered capillary loops (Figure 4F), indicating successful generation of OIR models.

Consistent with the postnatal downregulation of Zeb2 (Figure S1), its immunoreactivity was weak or absent in adult retinal astrocytes under normoxia (Fig. S8). Following hypoxic injury, however, similar to embryonic stages, Zeb2 was highly expressed in astrocytes surrounding the ONH, but appeared to be weak or absent from the more peripherally located astrocytes in either vascular or avascular areas (Figure S8). These results suggest that Zeb2 may be induced by hypoxia in APCs or APC‐like cells surrounding the ONH in both embryonic and OIR retinas. To investigate whether *Zeb2* inactivation affects pathological proliferation and activation of astrocytes, we focused on the phase of neovascularization (P14 and P17). Pax2^+^ astrocytes were immunolabelled and quantified in the AVAs and NVAs of both control and Zeb2CKO retinas. Interestingly, at P14, we observed a significant increase of astrocytes in the AVAs of Zeb2CKO retinas compared to the control (Figure 4B,C), and this difference remained in the AVAs between the two groups at P17 (Figure 4B,D). Moreover, there was a similar elevation of Pax2^+^ astrocytes in the NVAs of Zeb2CKO retinas at P17 (Figure 4B,E), suggesting that *Zeb2* ablation increases pathological activation of astrocytes in the OIR retina.

We next investigated whether pathological astrocyte activation would result in pathological vascular alterations in Zeb2CKO retinas following hypoxic injury. By immunolabelling retinal vasculature for IB4, we found that while the absence of *Zeb2* in astrocytes had no appreciable effect on AVAs of P14 Zeb2CKO retinas after hypoxic injury (Figure 4F,G), the AVAs and NVAs in P17 Zeb2CKO retinas were increased by 77.7% and 81.0%, respectively (Figure 4F,H,I). The more severe avascular and neovascular phenotypes indicate impeded revascularization and exacerbated neovascularization. Thus, Zeb2 may modulate both retinal neovascularization and revascularization by promoting pathological activation of astrocytes following hypoxic injury.

### 2.6 Zeb2 Regulates the Differentiation of Retinal Astrocytes After Hypoxic Injury

**FIGURE 5:**
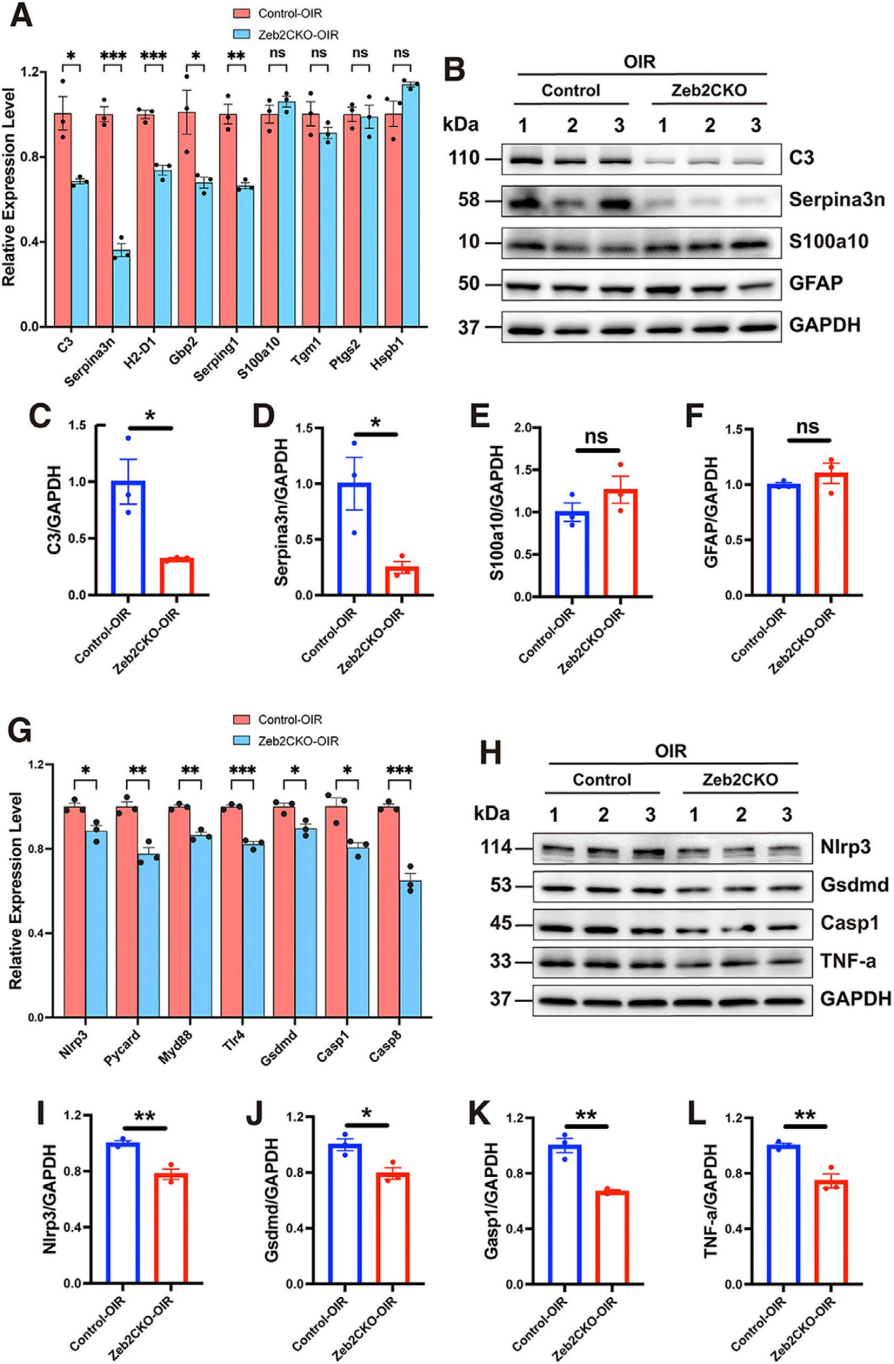
Astrocytic *Zeb2* ablation causes downregulation of A1 reactive astrocyte markers as well as Nlrp3 inflammasome pathway markers in P17 Zeb2CKO OIR retinas. (A) Relative RNA expression levels of the indicated genes were determined by qRT‐PCR assay in P17 control and Zeb2CKO OIR retinas. Data are presented as mean ± SEM (*n* = 3). *p < 0.05, **p < 0.01, ***p < 0.001; ns, no significance. (B) Western blot analysis of C3, Serpina3n, S100a10, and GFAP protein levels in retinas from 3 each P17 control and Zeb2CKO animals under the OIR condition. GAPDH served as the internal protein control. (C–F) Quantification of C3 (C), Serpina3n (D), S100a10 (E), and GFAP (F) protein levels in P17 control and Zeb2CKO OIR retinas. Data are presented as mean ± SEM (*n* = 3). *p < 0.05; ns, no significance. (G) Relative RNA expression levels of the indicated genes were determined by qRT‐PCR assay in P17 control and Zeb2CKO OIR retinas. Data are presented as mean ± SEM (*n* = 3). *p < 0.05, **p < 0.01, ***p < 0.001. (H) Western blot analysis of Nlrp3, Gsdmd, Casp1, and TNF‐a protein levels in retinas from 3 each P17 control and Zeb2CKO animals under the OIR condition. GAPDH served as the internal protein control. (I‐L) Quantification of Nlrp3 (I), Gsdmd (J), Casp1 (K), and TNF‐a (L) protein levels in P17 control and Zeb2CKO OIR retinas. Data are presented as mean ± SEM (*n* = 3). *p < 0.05; **p < 0.01.

Previous studies have shown that different pathological conditions induce different types of reactive astrocytes to regulate pathological progression. For example, inflammatory insult produces more neurotoxic (A1) reactive astrocytes, whereas an ischemic injury induces more neuroprotective (A2) reactive astrocytes [35]. We therefore examined the expression of known molecular markers for reactive astrocyte differentiation in control and Zeb2CKO retinas under hypoxic conditions. Under the OIR condition, qRT‐PCR assay revealed in P17 Zeb2CKO retinas a significant downregulation of *C3, Serpina3n, H2‐D1, Gbp2*, and *Serping1*, which are molecular markers for A1 reactive astrocytes, but no change of *S100a10, Tgm1, Ptgs2*, and *Hspb1*, molecular markers for A2 reactive astrocytes (Figure 5A). Consistent with this, Western blot analysis showed a dramatic reduction in the expression of both C3 and Serpina3n proteins but no significant alteration of S100a10 and GFAP protein expression in P17 Zeb2CKO OIR retinas (Figure 5B–F). These results indicate that astrocytic *Zeb2* inactivation may lead to a decrease in neurotoxic reactive astrocytes following hypoxic injury.

### 2.7 Astrocytic *Zeb2* Ablation Alleviates Retinal Neuroinflammation After Hypoxic Injury

**FIGURE 6:**
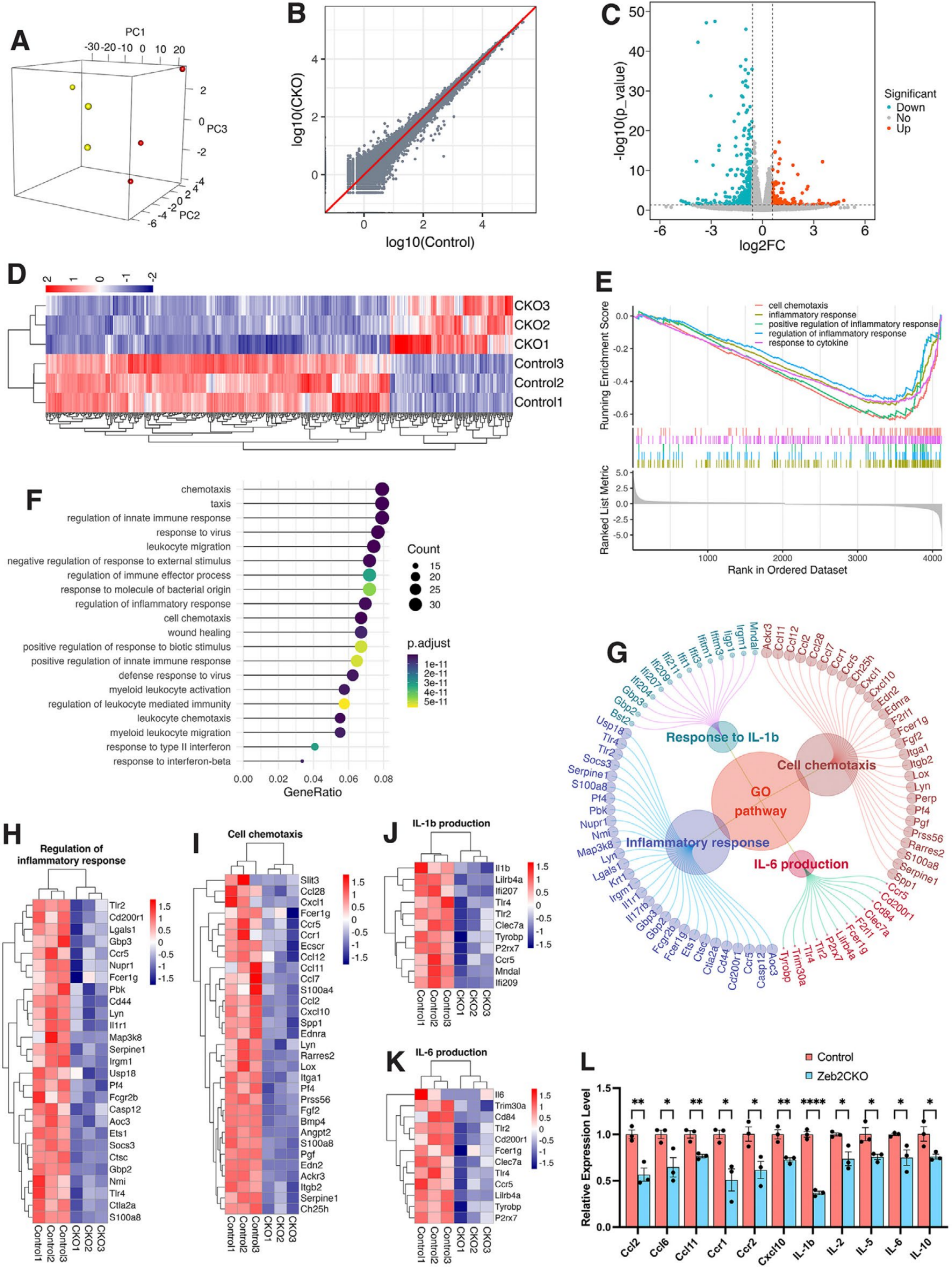
Transcriptome changes detected by RNA‐seq analysis between P17 OIR Zeb2CKO and control retinas. (A) Principal component (PC) analysis of RNA‐seq data showing that the Zeb2CKO retinal samples (red) are well discriminated from the control samples (yellow). (B) Scatter plot of global gene expression profiles in CKO and control retinas. Gene expression levels are depicted in log10 scale. The diagonal line represents equal expression in the two genotypes. (C) Volcano plot (significance vs. fold change) of significantly downregulated (green) and upregulated (red) genes (fold change ≥ 1.5 and *p* < 0.05) between the CKO and control retinas. (D) Heatmap of the DEGs reveals a large group of significantly downregulated genes as well as a smaller cluster of significantly upregulated ones in CKO retinas. (E) GSEA of the RNA‐seq data identifies enriched gene sets associated with inflammatory response, regulation of inflammatory response, positive regulation of inflammatory response, cell chemotaxis, and response to cytokine. (F) Top 20 enriched GO terms for the DEGs between the CKO and control retinas. (G) Network plot of 4 representative enriched GO terms or gene sets (nodes) and their associated DEGs. Node size represents the gene‐set size. (H‐K) Expression heatmaps of a set of DEGs involved in the regulation of inflammatory response (H), cell chemotaxis (I), IL‐1b production (J), and IL‐6 production (K) in P17 OIR control and CKO retinas. (L) qRT‐PCR assay of the RNA expression levels of the indicated chemokine‐related and interleukin genes in P17 OIR control and Zeb2CKO retinas. Data are presented as mean ± SEM (*n* = 3). *p < 0.05, **p < 0.01, ****p < 0.0001.

Given the cellular alterations observed in Zeb2CKO retinas following hypoxic injury, we further investigated the molecular changes in Zeb2CKO retinas under the OIR condition by RNA‐seq analysis. This analysis yielded a large group of downregulated genes and a small set of upregulated ones in P17 OIR Zeb2CKO retinas compared to controls (Figure 6A–D; Table S2). GSEA and GO enrichment analysis revealed that the DEGs are mostly enriched for genes involved in inflammatory and innate immune responses. They are associated with numerous inflammation‐related GO terms such as inflammatory response, regulation of inflammatory response, wound healing, cell chemotaxis, response to cytokine, interleukin‐1 beta (IL‐1b) production, regulation of innate immune response, and so on (Figure 6E–G). Separate GO enrichment, KEGG pathway enrichment, and GSEA analyses of only the downregulated DEGs led to similar results; for instance, enrichment for GO terms and pathways including positive regulation of inflammatory response, rheumatoid arthritis, activation of innate immune response, chemotaxis, chemokine signalling pathway, TNF signalling pathway, IL‐17 signalling pathway, etc. (Figure S9). These results suggest that astrocytic *Zeb2* inactivation results in the suppression of inflammatory responses in Zeb2CKO OIR retinas, consistent with the observed reduction of A1 reactive astrocytes, which are characterized by their production of pro‐inflammatory substances including cytokines and neurotoxins [35, 36, 37].

To validate the downregulation of inflammation‐associated genes in Zeb2CKO OIR retinas, we first examined the expression heatmaps of enriched sets of DEGs involved in relevant GO terms and pathways. As examples, this analysis revealed a downregulation of many genes responsible for the regulation of inflammatory response and cell chemotaxis, as well as downregulation of about a dozen genes each for IL‐1b and IL‐6 production (Figure 6H–K), indicating decreased inflammatory response in P17 Zeb2CKO retinas under the OIR condition. qRT‐PCR assay further confirmed in Zeb2CKO OIR retinas significant downregulation of CC and CXC chemokine signalling genes including *Ccl2, Ccl6, Ccl11, Ccr1, Ccr2*, and *Cxcl10*, and interleukin genes including *IL‐1b, IL‐2, IL‐5*, and *IL‐6* (Figure 6L). Consistent with dampened IL‐1b production, qRT‐PCR and Western blot analyses showed in Zeb2CKO OIR retinas reduced expression of *Nlrp3, Pycard, Myd88, Tlr4, Gsdmd, Casp1, Casp8*, and *Tnf/TNF‐a* (Figure 5G–L), all genes involved in Nlrp3 inflammasome activation characterized by the release of IL‐1b and other cytokines. Thus, astrocytic *Zeb2* ablation inhibits retinal neuroinflammatory responses following hypoxic injury, in agreement with reduced A1 reactive astrocytes.

### 2.8 Astrocytic *Zeb2* Ablation Leads to Elevated Tuft ECs but Decreased Tip ECs After Hypoxic Injury

**FIGURE 7:**
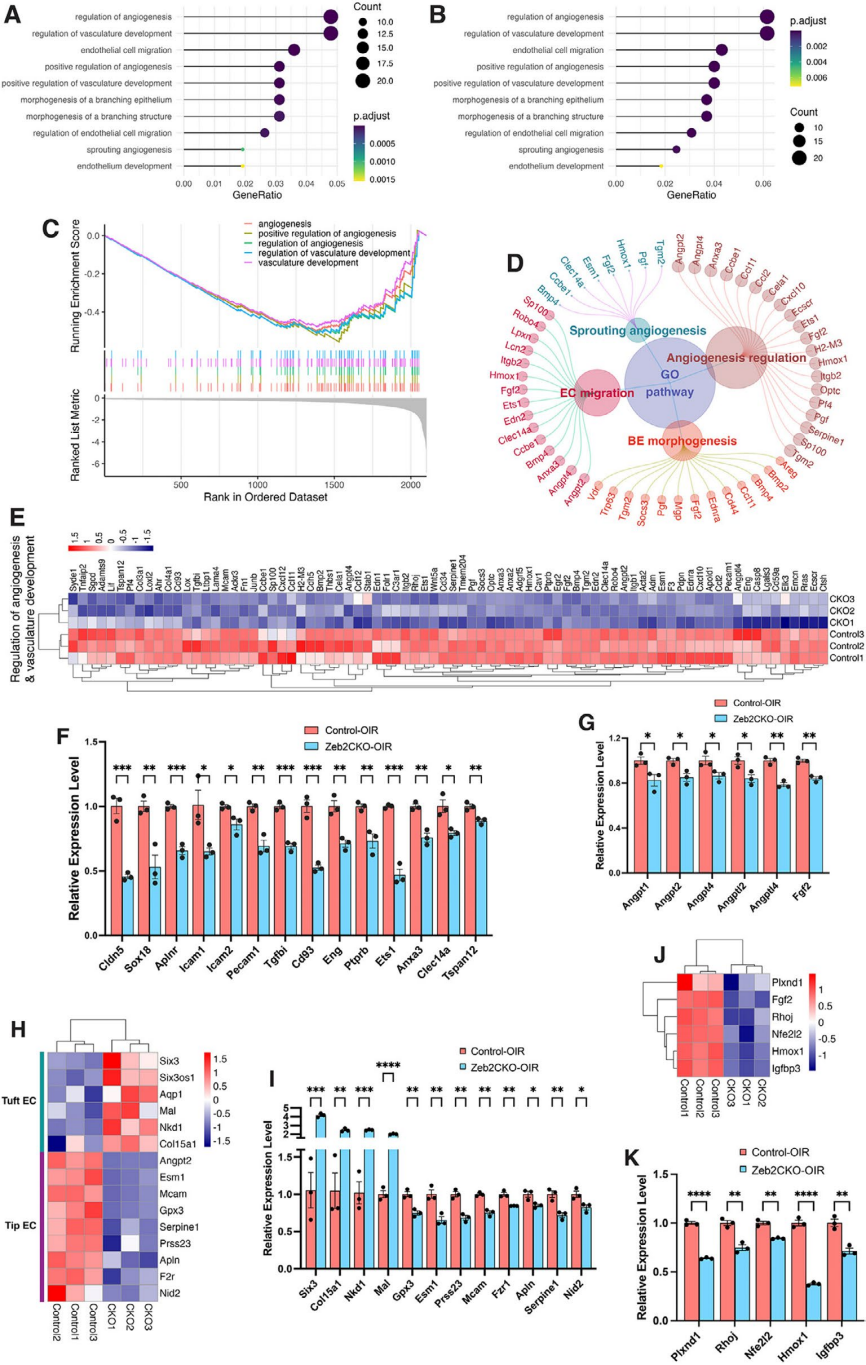
Increased neovascularization but diminished revascularization in P17 OIR Zeb2CKO retinas. (A, B) Representative vascularization‐related GO terms enriched for all DEGs (A) and downregulated DEGs (B) between P17 OIR Zeb2CKO and control retinas. (C) GSEA of the downregulated genes identifies enriched gene sets associated with angiogenesis, regulation of angiogenesis and vasculature development, positive regulation of angiogenesis, and vasculature development. (D) Network plot of 4 representative enriched vascularization‐related GO terms or gene sets (nodes) and their associated DEGs. Node size represents the gene‐set size. Abbreviations: BE, branching epithelium; EC, endothelial cell. (E) Expression heatmap of the set of downregulated genes involved in the regulation of angiogenesis and vasculature development. (F) qRT‐PCR assay of the RNA expression levels of the indicated vascularization‐related genes. Data are presented as mean ± SEM (*n* = 3). *p < 0.05; **p < 0.01; ***p < 0.001. (G) qRT‐PCR assay of the RNA expression levels of the indicated angiopoietin, angiopoietin‐like, and *Fgf2* genes. Data are presented as mean ± SEM (*n* = 3). *p < 0.05; **p < 0.01. (H) Expression heatmap of the tuft and tip EC marker genes. (I) qRT‐PCR assay of the RNA expression levels of representative tuft and tip EC marker genes. Data are presented as mean ± SEM (*n* = 3). *p < 0.05; **p < 0.01; ***p < 0.001; ****p < 0.0001. (J) Expression heatmap of a set of genes involved in revascularization and/or neovascularization. (K) qRT‐PCR assay of the RNA expression levels of several genes involved in neovascularization and/or revascularization. Data are presented as mean ± SEM (*n* = 3). **p < 0.01, ****p < 0.0001.

We explored the molecular basis underlying the pathological vascular changes in P17 Zeb2CKO OIR retinas. Apart from enrichment for inflammatory response genes, the above GO enrichment analysis also revealed that the DEGs in P17 Zeb2CKO OIR retinas are enriched for vascularization‐related GO terms including regulation of angiogenesis, sprouting angiogenesis, regulation of vasculature development, endothelial cell migration, endothelium development, morphogenesis of a branching epithelium, etc. (Figure 7A). Further GO enrichment and GSEA analyses of only the downregulated DEGs showed enrichment of similar vascularization‐related GO terms (Figure 7B–D), suggesting that astrocytic *Zeb2* ablation results in diminished overall vascularization in the OIR retina.

In line with decreased vascularization, expression heatmap showed that numerous genes associated with the regulation of angiogenesis and vasculature development are downregulated in P17 Zeb2CKO OIR retinas (Figure 7E). And we were able to confirm by qRT‐PCR assay the downregulation of many of the angiogenesis marker genes (e.g., *Cldn5, Pecam1, Eng*) (Figure 7F). Notably, the angiopoietin genes *Angpt2* and *Angpt4*, the angiopoietin‐like gene *Angptl4*, and *Fgf2* (*bFGF*) are also downregulated in the Zeb2CKO OIR retina as visualized by the expression heatmap (Figure 7E). In agreement, qRT‐PCR assay validated the significant downregulation of *Angpt1, Angpt2, Angpt4, Angptl2, Angptl4*, and *Fgf2* (Figure 7G). By contrast, there is no significant change in the expression of *Hif1a*, *Epas1(Hif2a), Vegfa, Slc2a1(Glut1)*, and *Ldha* in the mutant retina as determined by qRT‐PCR, Western blot and/or immunostaining analyses (Figure S10).

It has been reported recently that the neovascularization‐associated tuft ECs and the tip ECs, responsible for physiological revascularization, are both characterized by unique transcriptional signatures [38]. Based on the expression heatmap, we found that most tuft EC markers are upregulated while nearly all the tip EC markers are downregulated in the P17 Zeb2CKO OIR retina (Figure 7H). And this observation was validated by qRT‐PCR analysis (Figure 7I), indicating elevated tuft ECs and reduced tip ECs in the mutant retina, which may lead to exacerbated neovascularization but diminished physiological revascularization. Thus, the observed drop in overall vascularization in Zeb2CKO OIR retinas may be attributed to the net outcome of these two opposing effects on vascular development following hypoxic injury. To further explore the mechanism underlying the exacerbation of neovascularization and impediment of physiological angiogenesis in Zeb2CKO OIR retinas, we identified several genes (*Plxnd1, Rhoj, Nfe2l2/Nrf2, Hmox1, Fgf2, and Igfbp3*) whose expression is downregulated in the mutant retina as shown by the expression heatmap and qRT‐PCR assay (Figure 7G,J,K). These genes are known to prevent hypoxia‐induced neovascularization and/or promote reparative revascularization [39, 40, 41, 42, 43, 44], so their downregulation caused by *Zeb2* inactivation may result in more severe pathological angiogenic phenotypes under the OIR condition.

### 2.9 Astrocytic *Zeb2* Ablation Promotes the Proliferation and Migration of Primary Astrocytes

**FIGURE 8:**
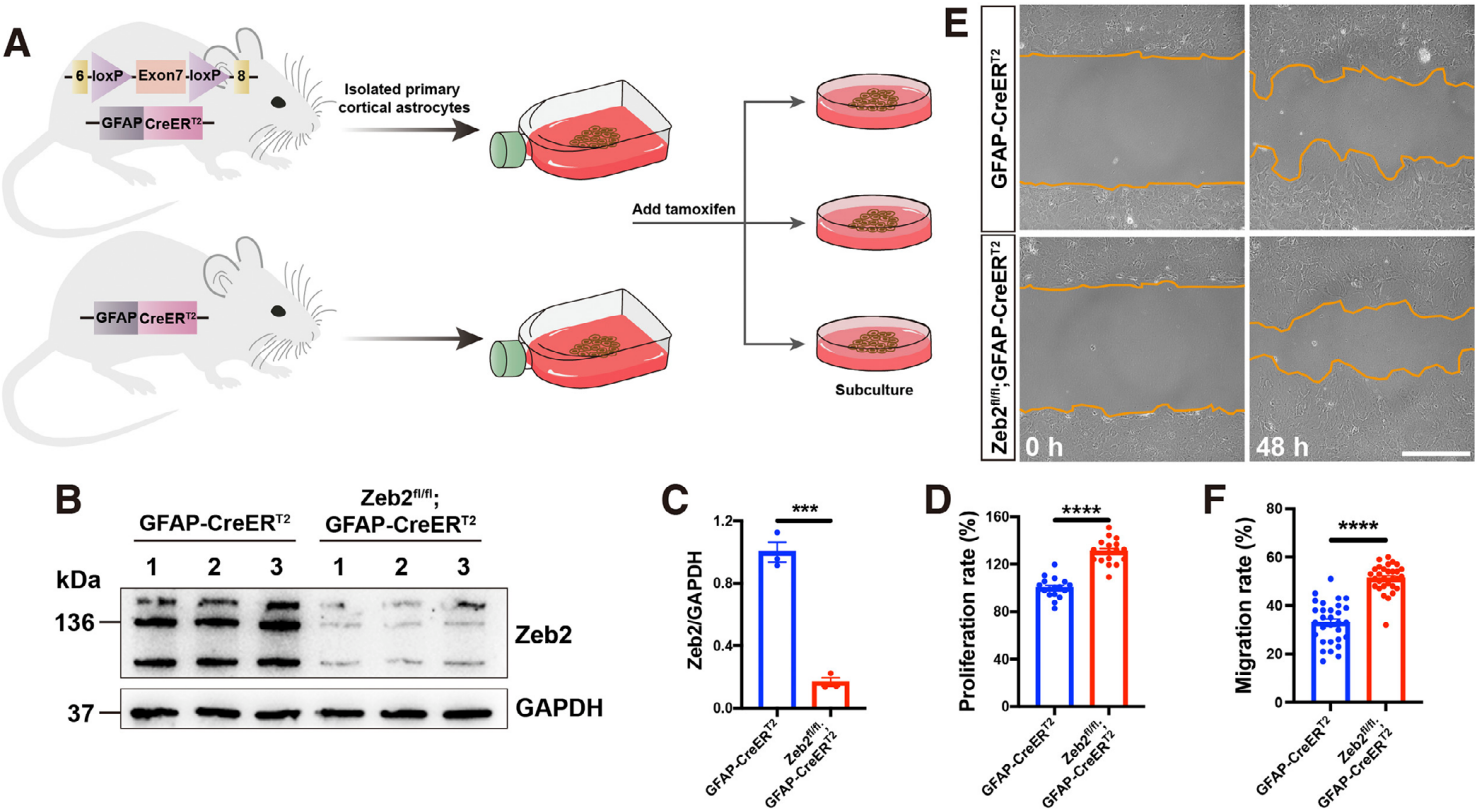
*Zeb2* ablation promotes the proliferation and migration of primary astrocytes (pACs). (A) Schematic diagram of the GFAP‐CreER^T2^ transgenic mice, Zeb2^fl/fl^; GFAP‐CreER^T2^ mice, and culture of the isolated pACs. pACs were isolated from P2 pups of the indicated genotypes, and tamoxifen was administered for *Zeb2* knockdown before experiments. (B) Western blot analysis of Zeb2 protein levels in pACs from GFAP‐CreER^T2^ and Zeb2^fl/fl^; GFAP‐CreER^T2^ mice in 3 independent experiments, 48 h post tamoxifen treatment. GAPDH served as the internal protein control. (C) Quantification of Zeb2 protein levels in pACs from GFAP‐CreER^T2^ and Zeb2^fl/fl^; GFAP‐CreER^T2^ animals, 48 h post tamoxifen treatment. Data are presented as mean ± SEM (*n* = 3). ***p < 0.001. (D) Quantification of cellular proliferation rate of pACs from GFAP‐CreER^T2^ and Zeb2^fl/fl^; GFAP‐CreER^T2^ mice, as determined by the CCK8 assay 48 h post tamoxifen treatment. Data are presented as mean ± SEM (*n* = 21 or 22). ****p < 0.0001. (E) Representative phase‐contrast images of pAC cell culture at the indicated timepoints following the removal of the culture‐insert during the wound healing assay. GFAP‐CreER^T2^ and Zeb2^fl/fl^; GFAP‐CreER^T2^ pACs were treated with tamoxifen for 48 h, followed by treatment with mitomycin C for 1 h, and were photographed at two timepoints: 0 and 48 h. (F) Quantification of cellular migration rate of GFAP‐CreER^T2^ and Zeb2^fl/fl^; GFAP‐CreER^T2^ pACs at 48 h during the wound healing assay. Data are presented as mean ± SEM (*n* = 30). ****p < 0.0001. Scale bar: F, 200 μm.

To investigate in vitro the effect of *Zeb2* inactivation on astrocytes, we isolated and cultured primary astrocytes (pACs) from the cerebral cortices of GFAP‐CreER^T2^ and Zeb2^fl/fl^; GFAP‐CreER^T2^ mice. The purity of the isolated pACs reached more than 95% as determined by immunolabelling for astrocyte‐specific protein markers (Figure S11). These cells were treated with tamoxifen following cell passage to delete *Zeb2* (Figure 8A). To monitor recombination specificity and efficiency, subsequent Western blot analysis showed that this procedure led to over 83% reduction in expression of the full‐length Zeb2 protein in Zeb2^fl/fl^; GFAP‐CreER^T2^ pACs (Figure 8B,C). Quantification of cell proliferation revealed that the Zeb2^fl/fl^; GFAP‐CreER^T2^ pACs displayed approximately 30.55% increase in cell proliferation rate compared to the control cells (Figure 8D). Likewise, in a wound healing test, the Zeb2^fl/fl^; GFAP‐CreER^T2^ pACs exhibited 55.41% increase in cell migration rate (Figure 8E,F). These results suggest that astrocytic *Zeb2* inactivation enhances the proliferation and migration of pACs, in agreement with the observed elevation in astrocyte proliferation and migration in Zeb2CKO retinas.

## 3 Discussion

**FIGURE 9:**
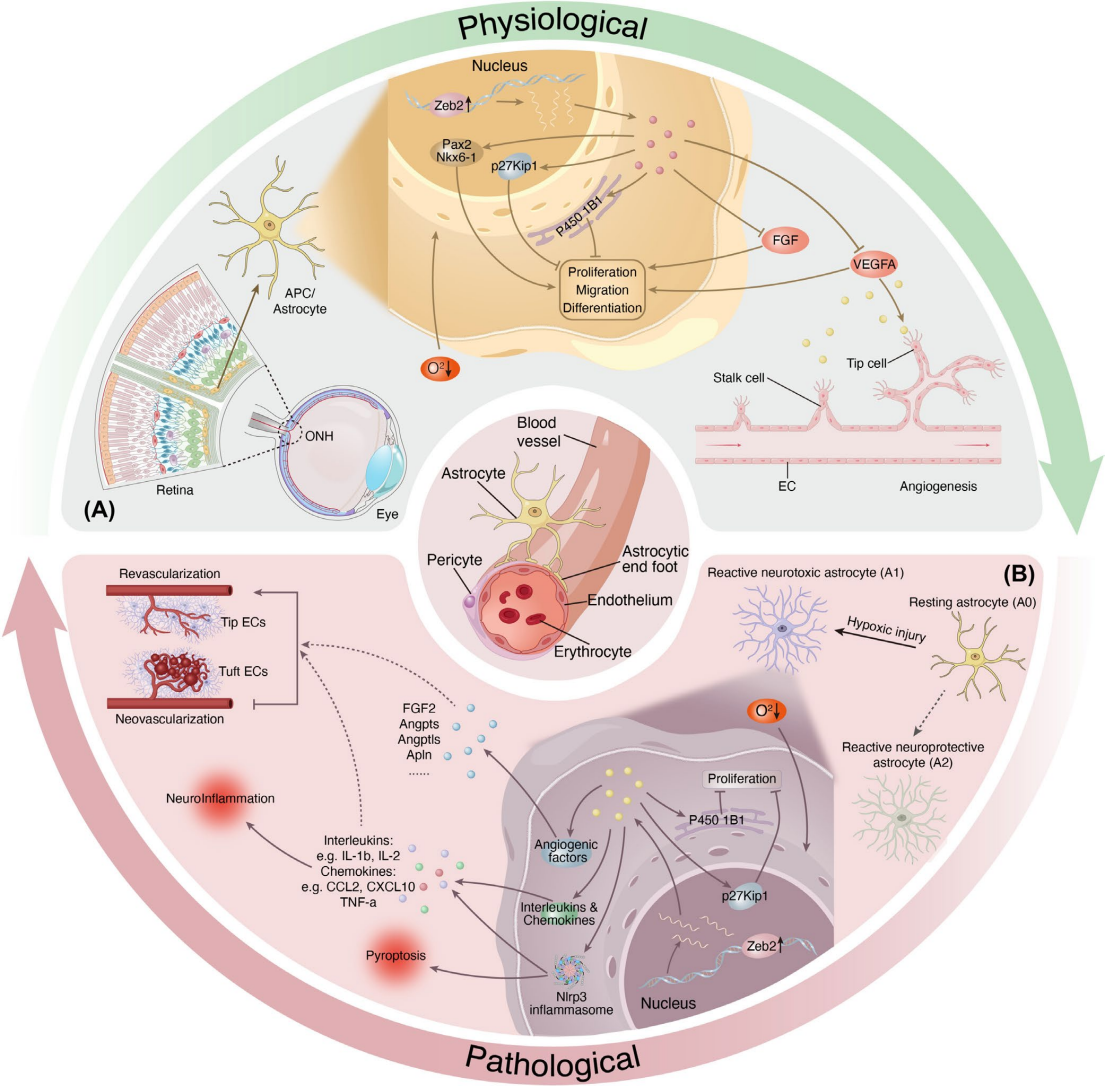
Working model of how astrocytic Zeb2 may regulate retinal angiogenesis under physiological and pathological conditions. (A) During late embryonic development, retinal APCs in the ONH region sense hypoxic signals and upregulate Zeb2 expression in response. Zeb2 in turn directly and/or indirectly modulates the expression of downstream targets (e.g., VEGFA, p27Kip1, Pax2, and FGF) to restrain astrocyte proliferation, migration and maturation. In particular, it directly represses *Vegfa* expression to prevent excessive and disorganized angiogenesis in the superficial layer. (B) Under hypoxic injury, Zeb2 is re‐induced and promotes the differentiation of the neurotoxic A1 astrocytes that, while inflammatory, may be required for balanced physiological revascularization and suppression of pathological neovascularization. A shift in astrocyte identity may result in elevated release of pro‐angiogenic factors (e.g., FGF2, Angpts and Angptls), interleukins (e.g., IL‐1b and IL‐2) and chemokines (e.g., CCL2 and CXCL10), creating a milieu that promotes reparative tip ECs over pathogenic tuft ECs. APC, astrocyte progenitor cell; EC, endothelial cell; ONH, optic nerve head.

In this study, we have identified Zeb2 as a critical oxygen‐sensing regulator of retinal astrocyte development and function, with profound implications for both physiological and pathological angiogenesis (Figure 9). Our findings demonstrate that Zeb2 is transiently expressed in retinal APCs and differentiating astrocytes during embryonic development. Conditional ablation of *Zeb2* in astrocytes led to enhanced proliferation and migration, and precocious maturation of these cells, resulting in an overproduction of astrocytes and a denser glial network. This, in turn, caused a transient but significant overgrowth of the superficial retinal vasculature in early postnatal stages, characterized by increased vascular density and aberrant remodelling. Importantly, in a murine model of pathological angiogenesis (OIR), astrocytic *Zeb2* ablation exacerbated the disease outcome by worsening pathological neovascularization and impeding physiological revascularization. Mechanistically, we found that Zeb2 regulates the differentiation of activated astrocytes following hypoxic injury, and that its inactivation promotes a shift away from a neurotoxic (A1) phenotype, which results in dampened neuroinflammation, elevated tuft ECs but decreased tip ECs. These multifaceted roles of astrocytic Zeb2 position it as a key coordinator of the retinal neurovascular unit in health and disease.

Our work provides both in vivo and in vitro evidence indicating the expression of Zeb2 in retinal astrocytes is dynamically regulated by oxygen levels, aligning with the observation in some other cell types. For instance, in glioma cells, hypoxic conditions similarly upregulate Zeb2, mediating repression of ephrinB2 and enhancing invasive behaviours [45]. In the developing retina, Zeb2 is transiently expressed in APCs during embryonic physiological hypoxia but downregulated upon vascularization and oxygenation postnatally. Notably, its expression is re‐induced by pathological hypoxia in the OIR model, specifically in APC‐like cells surrounding the ONH. This hypoxia‐responsive regulation distinguishes it from some other cell types (e.g., retinal cells) where Zeb2 expression may be constitutive or regulated by different cues [23, 24]. Functionally, this expression pattern positions Zeb2 as a critical oxygen‐sensing transcriptional switch: during development, it restrains astrocyte proliferation and maturation to ensure precise template formation for physiological angiogenesis; whereas under pathological hypoxia, it promotes a neurotoxic A1 astrocyte response that appears to be necessary for certain aspects of the reparative angiogenesis while mitigating pathological neovascularization (Figure 9). Thus, Zeb2 acts as an oxygen‐dependent modulator of astrocyte function, with contrasting outcomes in developmental versus pathological angiogenesis.

Our finding that Zeb2 is transiently expressed in migrating and differentiating retinal APCs provides a crucial temporal context for its function. This expression pattern suggests Zeb2 acts as a key modulator during a critical window of astrocyte development, likely fine‐tuning the genetic programs that control their proliferation, migration, and integration into the nascent glial network. The observation that *Zeb2* ablation enhanced all these processes indicates that its primary role in developing retinal astrocytes is to restrain and precisely time their expansion and maturation. This is consistent with its known function as a transcriptional repressor in other contexts [45, 46, 47]. At the molecular level, our transcriptome profiling indeed revealed in Zeb2CKO retinas upregulation of *Vegfa*, *Pax2* and *Nkx6‐1*, which are key genes implicated in astrocyte specification and differentiation [12, 29, 30, 31]. The observed *Fgf17* and *Fgf23* upregulation may additionally contribute to excessive APC generation in the mutant given that FGF signalling promotes the APC fate [48]. Moreover, increased expression of VEGFA, which is an implicated promoter of astrocyte proliferation [12], as well as downregulated expression of Cyp1b1 and Cdkn1b, which inhibit astrocyte proliferation [32, 33], is expected to contribute to the expansion of APCs in the Zeb2CKO retina (Figure 9). HIF2a is required to induce *Vegfa* expression and stimulate astrocyte proliferation [16]. However, Zeb2 does not appear to regulate *Hif2a* to exert its inhibitory effect on APC proliferation and *Vegfa* expression given the unaltered *Hif2a* expression in the mutant retina.

The astrocytic template is indispensable for orchestrating the patterned growth of retinal blood vessels. The initial overproduction of astrocytes in Zeb2CKO mice provided an altered scaffold and molecular microenvironment, which directly correlated with the excessive and disorganized growth of the superficial vascular plexus. This phenotype, including increased EC number and vascular density, and abnormal arterial/venous branching, underscores the sensitivity of vascular development to the precise quantity and spatial organization of astrocytes. The fact that these vascular defects were transient and resolved by P12, coinciding with the normalization of the astrocyte network, strongly supports a model where Zeb2 indirectly controls early angiogenesis by first ensuring the proper development of the astrocytic template. This aligns well with a previous study showing that astrocyte dysregulation disrupts vascular patterning [16]. Given the essential role of astrocyte‐derived VEGFA in radial migration of ECs and formation of the exquisite superficial vascular plexus [12], Zeb2 may function to fine‐tune this process by maintaining VEGFA at a proper level through direct repression of *Vegfa* expression.

A unique finding of this study is the seemingly paradoxical role of Zeb2 in pathological settings. Contrary to its restrictive role in physiological development, *Zeb2* ablation in the OIR model led to a more severe pathological outcome. This paradox may be explained by the altered response of *Zeb2*‐deficient astrocytes to hypoxia. In particular, loss of *Zeb2* markedly reduced the expression of neurotoxic A1 astrocyte markers such as complement C3 and Serpina3n, thereby causing a shift of the mutant astrocytes to a more homeostatic identify. Consequently, we observed that *Zeb2*‐deficient astrocytes expressed reduced transcripts for inflammatory interleukins (e.g., IL‐1b, IL‐6), CC and CXC chemokines (e.g., Ccl2, Cxcl10), as well as Nlrp3 inflammasome factors (e.g., Gsdmd, TNF‐a) (Figures 5 and 6). This finding suggests that Zeb2 normally promotes an astrocyte secretory program rich in inflammatory/angiogenic mediators (Figure 9), and that its inactivation blunts this program. While a reduced inflammatory response is often considered beneficial, in the complex context of OIR, the A1 astrocyte‐derived factors might be necessary for initiating certain reparative processes or for communicating with immune cells that help resolve damage. Consistent with this idea, in Zeb2CKO retinas, there is significant downregulation of pro‐angiogenic factor genes including angiopoietin and angiopoietin‐like genes *Angpt1, Angpt2, Angpt4, Angptl2*, and *Angptl4*, fibroblast growth factor gene *Fgf2*, and apelin gene *Apln* (Figure 7). In addition, the increased astrocytes in avascular and neovascular areas likely create a more hostile microenvironment, impeding reparative revascularization and exacerbating the formation of pathological tufts in the mutant retina. This is precedented by a previous report demonstrating the number of astrocytes correlates with the severity of vascular abnormalities in a different OIR mouse model [16].

Further supporting that *Zeb2*‐deficient astrocytes have altered properties in response to hypoxia, our molecular analysis revealed that the exacerbated pathological angiogenesis in Zeb2CKO OIR retinas was associated with a transcriptional signature favouring pathological tuft ECs over reparative tip ECs (Figure 9). This switch is evident in the Zeb2CKO retina since most genes of tip EC markers display a downregulation whereas those of tuft EC markers exhibit an upregulation as determined by RNA‐seq and qRT‐PCR analyses. The downregulation of key revascularization‐promoting and/or neovascularization‐preventing genes (e.g., *Igfbp3, Plxnd1, Rhoj, Nfe2l2, Hmox1, Fgf2*) may partly provide a genetic explanation for the impeded physiological revascularization and expanded neovascularization. For instance, disrupting *Igfbp3* in mice causes an increase of retinal vaso‐obliteration by reducing tip ECs while administration of its protein promotes reparative vascular regrowth [41]. *Nfe2l2* plays a crucial role in both neovascularization and revascularization [39]: its deficiency not only inhibits retinal revascularization but also increases pathological neovascularization in OIR mice. In contrast, pharmacologic *Nfe2l2* activation enhances physiological angiogenesis as well as prevents pathologic neovascularization. Similar to Nfe2l2 and as one of its targets, hemeoxygenase‐1 (Hmox1) facilitates reparative angiogenesis and alleviates pathological neovascularization [42, 43, 49]. FGF2 is also well‐known for its activity to mitigate neovascularization and promote revascularization [44]. Plxnd1 and Rhoj, on the other hand, are key components of the Sema3E‐Plexind1 signalling pathway that suppresses pathological neovascularization by inhibiting the disoriented angiogenesis, without affecting reparative revascularization [40]. Interestingly, the apparent change of *Zeb2*‐deficient astrocytes in response to hypoxia appears to occur independently of HIFs and VEGF (Figure S10), highlighting a novel, HIF/VEGF‐independent pathway through which Zeb2, via astrocytes, may modulate the pathological vascular response.

In summary, our work establishes Zeb2 as an essential oxygen‐sensing regulator that calibrates astrocyte generation and reactivity. It acts as a brake on astrocyte proliferation and maturation during development to ensure the precise formation of the astrocytic and vascular networks. However, in response to pathological insult, Zeb2 is required to promote a specific neurotoxic (A1) astrocyte response that, while inflammatory, appears to be a necessary component of the complex response to injury, and its absence leads to a worsened vascular outcome. In particular, Zeb2 appears to be necessary for the balanced astrocyte response that fosters reparative revascularization while restraining aberrant tuft formation during hypoxic injury. Therefore, therapeutic strategies aimed at modulating Zeb2 activity in astrocytes must be context‐specific. Enhancing Zeb2 function is most likely beneficial in suppressing excessive astrogliosis and anomalous angiogenesis in developmental disorders or hypoxic injury, while inhibiting Zeb2 could be a strategy to mitigate neuroinflammation in certain pathological conditions. These properties make Zeb2 a compelling target for novel therapies targeting neurovascular diseases.

## 4 Experimental Procedures

### 4.1 Animals

The astrocyte‐specific *Zeb2* knockout mice were generated by mating between GFAP‐Cre or GFAP‐CreER^T2^ and *Zeb2* ^*fl/fl*^ animals with a C57BL/6 background and maintained in a standard SPF level animal facility at Zhongshan Ophthalmic Center, Sun Yat‐sen University. Conditional knockout mice (Zeb2^fl/fl^; GFAP‐Cre or Zeb2^fl/fl^; GFAP‐CreER^T2^) were obtained by breeding a floxed *Zeb2* mouse line [27] with the GFAP‐Cre or GFAP‐CreER^T2^ transgenic line to delete exon 7 of *Zeb2* in astrocytes. The two Cre driver transgenic lines were purchased from the Jackson laboratory, including the GFAP‐Cre line originally generated by Albee Messing (Jax stock number 004600) [50] and the GFAP‐CreER^T2^ line originally generated by Flora Vaccarino (Jax stock number 012849) [51]. Primary brain cortical astrocytes from GFAP‐CreER^T2^ and Zeb2^fl/fl^; GFAP‐CreER^T2^ animals were treated with tamoxifen to obtain control and *Zeb2*‐deficient cells. Genotyping for *Zeb2* mutations was conducted as described previously [23] (Figure S2A). The C57BL/6 mice were purchased from GemPharmatech Co. Ltd. (China). The starting stage of mouse embryos was defined by taking the morning as E0.5 when the copulation plug was seen. All animal experiments were performed according to the Institutional Animal Care and Use Committee (IACUC Z2023068) of Zhongshan Ophthalmic Center, Sun Yat‐sen University, and adhered to the ARVO Statement for the Use of Animals in Ophthalmic and Vision Research.

### 4.2 Immunofluorescence

#### 4.2.1 Retinal Wholemount Immunostaining

To analyse postnatal neovascularization in the mouse retina, littermates were euthanized at the indicated postnatal day. The eyes were fixed in 4% paraformaldehyde (PFA) solution for 30 min, transferred into pre‐cooled phosphate‐buffered saline (PBS), and then the retinas were dissected. The retinas were permeabilized and blocked in 5% normal donkey serum (NDS) with 0.3% PBS‐T (PBS with 0.3% Triton X‐100) for 1 h at room temperature (RT). They were then incubated overnight at 4°C with desired primary antibodies (Table S3) in 2.5% NDS with 0.3% PBS‐T. Following the incubation, the retinas were washed three times with PBS and incubated for 1 h at RT with secondary antibodies (Table S4) in 2.5% NDS with 0.3% PBS‐T. After incubation, the retinas were washed 3 times with PBS and flat‐mounted for observation and analysis with a laser scanning confocal microscope.

#### 4.2.2 Eye Section Staining

After fixation, the eyes were briefly rinsed with PBS and then immersed in 30% sucrose in PBS at 4°C for up to 6 h. The cornea and lens were removed under the dissection microscope, and the rest of the eye balls were processed for O.C.T. compound embedding. Tissue blocks were sectioned at 15‐μm thickness with a cryostat. The sections with tissues were briefly rinsed with PBS, permeabilized, and blocked in 10% NDS with 0.5% PBS‐T for 1 h at RT, followed by incubation overnight at 4°C with primary antibodies (Table S3) in 2.5% NDS with 0.5% PBS‐T. The following day, the samples were washed three times in PBS and then incubated for 1 h at RT with secondary antibodies (Table S4) and 4′6‐diamidino‐2‐phenylindole (DAPI) (Invitrogen, USA) diluted in 2.5% NDS with 0.5% PBS‐T. Following incubation, the sections were washed three times with PBS and mounted for analysis.

#### 4.2.3 Cell Staining

Primary astrocytes were cultured for 2 days after passaging, then fixed with 4% PFA for 15 min and washed twice with PBS. Cells were blocked in 10% NDS with 0.1% PBS‐T for 1 h at RT, followed by incubation with primary antibodies (Table S3) diluted in 2.5% NDS with 0.1% PBS‐T for 2 h at RT. After washing with PBS, cells were incubated with secondary antibodies (Table S4) and DAPI diluted in 2.5% NDS with 0.1% PBS‐T for 1 h at RT. Following a final wash with PBS, samples were mounted for analysis.

### 4.3 Haematoxylin–Eosin (HE) Staining and Thickness Measurement

After euthanizing the mice followed by cervical dislocation, the eyeballs of the mice were extracted, and excess tissue on the surface of the eyeballs was removed. They were then placed in the FAS eyeball fixative (G1109; Servicebio, China) and fixed overnight at 4°C. The fixed eyeballs were embedded in paraffin and sectioned with a thickness of 15 μm using a microtome. Sections from the central optic disc area of the retina were selected for HE staining. The processes of sectioning and staining were carried out by Servicebio Co. Ltd.

### 4.4 EdU Injection and Labeling

The EdU solution was intraperitoneally injected into pregnant mice on day 17.5 of gestation at a concentration of 5 mg/kg and the animals were sacrificed after 2 h. Analysis of EdU incorporation was performed using the Click‐iT Plus EdU kit (Thermo Fisher Scientific, USA). Retinal flat‐mount preparations were washed in PBS for 5 min and then incubated in 5% NDS with 0.5% PBS‐T for 1 h at RT. Retinas were then incubated with the corresponding primary and secondary antibodies. Subsequently, EdU detection components were resuspended according to the manufacturer's instructions. 1X Click‐iT reaction buffer, CuSO_4_, Alexa Fluor azide, and 1X reaction buffer additive were added in order to prepare a reaction suspension according to the instructions. After incubation in the reaction suspension for 30 min at RT, samples were washed with PBS 3 times and mounted for detection.

### 4.5 Fundus Imaging and Fluorescein Fundus Angiography (FFA)

To detect vascular leakage in the mouse retina, all the FFA images were taken using a retinal‐imaging microscope as previously described [52]. Mice were anaesthetised with intraperitoneal injection of sodium pentobarbital (50 mg/kg, Tocris Bioscience, UK), and 0.5% dicaine hydrochloride eye drops (Zhongshan Ophthalmic Center, China) were used for local ocular surface anaesthesia. Pupils were dilated using 0.5% tropicamide and 0.5% phenylephrine hydrochloride (Shenyang Xingqi Pharmaceutical Co. Ltd., China). Following the dilation of pupils, the eyes were immersed with 1% hypromellose eye drop (Zhongshan Ophthalmic Center) to keep the cornea moist. Images of fundi were captured using a Micron IV mouse fundus camera (Phoenix Research Laboratories Inc., USA). To conduct FFA, fluorescein sodium (150 mg/kg, Alcon Research LLC, USA) was injected intraperitoneally. Images were taken immediately after fluorescein injection and then 5 min later to show vascular leakage accumulation.

### 4.6 Establishment of Oxygen‐Induced Retinopathy (OIR) Mouse Models

The OIR mouse models were established using the method described in previous studies [53, 54]. In brief, during postnatal stages P0 to P7, which is the period of angiogenesis, mice were kept at normoxic conditions (room air). They and their mothers were transferred to hyperoxic conditions of 75% O_2_ for 5 days from P7 to P12, causing retinal vaso‐obliteration. Finally, the pups were placed in room air from P12 under normoxic conditions to induce pathologic retinal neovascularization. During this process, the pups were sacrificed at desired time points by exposure to CO_2_ followed by cervical dislocation. Eyeballs were removed and prepared as described above. The pups used in this experiment all weighed over 6 g.

### 4.7 Quantitative Real‐Time PCR (qRT‐PCR)

Retinas were harvested from control and Zeb2CKO mice under normoxia and OIR conditions. RNA was prepared using the TRIzol reagent (Abcam, UK) and converted to cDNA using the HiScript III RT SuperMix (Vazyme Biotech Co., China). One microgram of total RNA per reaction was used for qRT‐PCR analysis. Reactions were set up in MicroAmp Fast Optical 384‐well reaction plates using the Kapa SYBR Fast qPCR Kit (NIPPON Genetics, Japan) and run in the qTOWER3 G Real‐Time PCR system (Analytik Jena, DEU). The expression level of each gene was quantified by the comparative cycle threshold method (2^−ΔΔct^ calculation method). All data were tested for significance using the multiple *t*‐test. The primer sequences used for qRT‐PCR are listed in Table S5.

### 4.8 Western Blotting

Retinas from P0 control and Zeb2CKO mice as well as cultured primary astrocytes (GFAP‐CreER^T2^ and Zeb2^fl/fl^; GFAP‐CreER^T2^) were collected and lysed in the RIPA buffer (Thermo Fisher Scientific) containing 1× protease and phosphatase inhibitor cocktail (Beyotime Biotechnology, China), followed by incubation for 30 min at 4°C. The lysate was subsequently centrifuged for 15 min at 14,000 × *g* at 4°C to separate the cellular debris. Then, the supernatant was transferred into a new tube and the protein concentration was determined using the enhanced BCA protein assay kit (Beyotime Biotechnology). Protein lysates were diluted in the protein loading buffer (Beyotime Biotechnology), heated to 95°C for 10 min, and stored at −20°C until use. 10 μg of total protein was separated by SDS‐PAGE (Beyotime Biotechnology) and subsequently transferred onto a 0.45 μm polyvinylidene difluoride transfer membrane using a Bio‐Rad transfer system for 60 min (300 mA). After transfer, membranes were blocked in the blocking buffer containing 5% skim milk (BD Biosciences, USA) in TBST (TBS with 0.1% Tween‐20) for 1 h at RT. They were then incubated overnight with primary antibodies diluted in blocking buffer at 4°C. The following day, the membranes were washed three times with TBST and subsequently incubated with secondary antibodies diluted in blocking buffer for 1 h at RT. Finally, membranes were washed three times for 10 min each. Target proteins were visualized with SuperSignal West Pico PLUS (Thermo Fisher Scientific) and imaged using G:BOX Chemi XT4 (Syngene, India). Detailed antibody information is provided in Tables S3 and S4.

### 4.9 RNA‐Seq Analysis

RNA‐seq analysis was carried out as described previously [55]. Total RNA of control and Zeb2CKO retinas was isolated at P0 or P17 under normoxia and OIR conditions. RNA‐seq libraries were prepared using the Hieff NGS Ultima Dual‐mode mRNA Library Prep Kit (Yeasen Biotechnology, China) and sequenced on an Illumina NovaSeq X plus sequencer (BioMarker Technologies CO. LTD., China). The obtained reads were trimmed and then aligned to the mouse reference genome (mm10) with STAR [56], followed by computation of gene expression changes using DESeq2 [57]. Principal component analysis, and scatter and volcano plot analyses of gene expression levels were performed using the R software. GO enrichment and KEGG enrichment analyses were performed using clusterProfiler and gene set enrichment analysis (GSEA) [58, 59]. RNA‐seq data reported in this paper were deposited in the NCBI SRA database under accession numbers SRR35773322‐SRR35773333.

### 4.10 Chromatin Immunoprecipitation (ChIP) Assay

The ChIP assay was conducted using the BeyoChIP Enzymatic ChIP Assay Kit (Beyotime Biotechnology) with modifications as previously described [60]. Retinas of 8‐week‐old C57BL/6 mice were cross‐linked with 1% formaldehyde and used for chromatin extraction. Chromatin was fragmented to around 100 to 600 bp via micrococcal nuclease digestion. Immunoprecipitation was carried out overnight at 4°C using 2 μg of the Zeb2 antibody or control IgG (Table S3). Purified DNA was subjected to qRT‐PCR analysis. Putative Zeb2 binding motifs were identified using JASPAR (http://jaspar.genereg.net). The sequences of all primers used are listed in Table S6.

### 4.11 CUT&Tag Analysis

CUT&Tag analysis was performed as previously described [60]. Briefly, retinas from C57BL/6 mice at E17.5 were dissociated into single‐cell suspensions, filtered to remove debris, and processed for CUT&Tag assay. For each reaction, 5 × 10^5^ cells were immobilized on Concanavalin A beads and incubated overnight at 4°C with 2 μg of primary antibodies (anti‐Zeb2 for the target protein, anti‐H3K27me3 as positive control, and IgG as negative control) with rotation. After washing, cells were incubated with species‐matched secondary antibodies for 1 h at RT with shaking, followed by incubation with the assembled pA‐Tn5 transposase complex for targeted DNA fragmentation. Purified DNA fragments were amplified into sequencing libraries, which were quality‐controlled using a bioanalyzer prior to sequencing. The ensuing bioinformatic analyses were conducted according to previous description [55, 60].

### 4.12 Dual‐Luciferase Reporter Assay

The full‐length Zeb2 coding sequence was subcloned into the pCI expression vector (Youbio Technology Co. Ltd., China) as an effector, while the promoter (2 kb upstream of the TSS) of *Vegfa* was inserted into the pGL3‐Basic reporter vector (Promega, USA) as a reporter. As previously described [60], a dual‐luciferase reporter assay was conducted. The effector and reporter plasmids were cotransfected into 293T cells together with the control Renilla luciferase reporter vector pRL‐TK (Promega). After transfection and incubation, luciferase activities were measured using the Dual‐Luciferase Reporter Assay System (Beyotime Biotechnology) and the Infinite M200 Pro Nanoq microplate reader (TECAN, Switzerland). The assays were independently repeated at least three times.

### 4.13 Culture of Primary Astrocytes (pACs)

Mouse cortical astrocytes were isolated from P2 GFAP‐CreER^T2^ and Zeb2^fl/fl^; GFAP‐CreER^T2^ mouse pups. In short, before starting the dissection procedure, each T75 flask (NEST, Switzerland) was coated with 20 mL of 50 μg/mL poly‐D‐lysine (Gibco, USA) in ddH_2_O for 1 h at 37°C. Mouse brains were removed at P2 from timed pups and quickly placed in ice‐cold HBSS (Gibco) containing 1% penicillin and streptomycin (Gibco). Olfactory bulbs, hippocampus, subcortical structures, and meninges were carefully removed. Four dissected cortices in a batch were transferred into a new 3.5 cm dish with 5 mL 0.25% trypsin–EDTA (Gibco). They were sliced into 1 mm^2^ chunks and incubated at 37°C for 30 min with gentle shaking every 10 min. Digested tissues were centrifuged at 300 g for 5 min at RT and the supernatant was carefully removed. Cell chunks were vigorously pipetted about 20 times in 5 mL astrocyte medium, consisting of DMEM (Gibco) with 10% fetal bovine serum (Gibco) and 1% pen‐strep, to ensure that the cortex tissue was dissociated into single cells. Next, poly‐D‐lysine was aspirated from the T75 culture flask and washed 3 times with ddH_2_O. Single‐cell suspension was adjusted to a volume of 20 mL using the astrocyte medium, transferred into the T75 culture flask, and incubated at 37°C in the CO_2_ incubator. Medium change was carried out every 3 days thereafter. After 8 days, T75 flasks were placed directly on an incubator shaker (ZHICHENG Type ZWY‐2120C, China), shaken for at least 20 h at 100 rpm at 37°C, and then washed twice with 10 mL of DPBS lacking Ca^2+^ and Mg^2+^ (Gibco). This was followed by three rounds of vigorous manual shaking for 30 s each in 10 mL of DPBS to remove any contaminating microglia and oligodendrocyte precursor cells. Adherent astrocytes were dissociated by incubation in 5 mL of 0.25% trypsin diluted in DPBS for 5 min at 37°C, and plated in the astrocyte medium. They were passaged a maximum of two times for experimental use.

### 4.14 CCK8 Assay

Cell proliferation was analyzed using the CCK‐8 Cell Counting Kit (CCK8, Vazyme, China) according to the manufacturer's protocol. pACs were seeded and cultured at a density of 5 × 10^3^/well in 100 μL of the astrocyte medium containing 1 μM/mL tamoxifen in 96‐well microplates (Corning, USA). After treatment for 48 h, CCK8 reagent (10 μL) was added to each well and then cultured for 2 more hours. The absorbance was measured at 450 nm using a BioTek Synergy HTX (Agilent, USA), using wells without cells as blanks. The proliferation rate of pACs was expressed by the absorbance. All experiments were performed in triplicate.

### 4.15 Wound Closure Migration Assay

GFAP‐CreER^T2^ and Zeb2^fl/fl^; GFAP‐CreER^T2^ pACs were trypsinized and counted as described above. 2 × 10^5^ cells were seeded into a 3‐well culture insert (Ibidi, Germany) in a 12‐well plate. They were cultured overnight in the astrocyte medium, which was changed the next day with astrocyte medium containing 1 μM/mL tamoxifen. After 48 h, pACs were treated with mitomycin C (1 μg/mL) for 1 h to block cell proliferation. The culture insert was then removed to generate wound‐like gaps. The cells were washed with DPBS twice and cultured in DMEM with 0.2% fetal bovine serum and 1% pen‐strep. The gaps were imaged at two time points (0 and 48 h) using an inverted optical microscope to monitor gap closure.

### 4.16 Statistical Analysis

Images of stained retinal sections or wholemounts were taken using the same parameters with a Zeiss LSM700 confocal microscope. The grayscale signal of the Western blot was captured using a G:BOX Chemi XT4. Fluorescent intensity, retinal thickness, distance of wound closure and signal in immunoblotting were all measured by the ImageJ software using auto threshold selection. Statistical analysis was calculated and performed using the Microsoft Excel Software and GraphPad Prism 9. All data were shown as mean ± SEM with sample quantity and statistical details for each experiment indicated in the corresponding figures and figure legends. The unpaired two‐tailed Student's parametric *t*‐test was used to assess differences between two groups, and one‐way parametric ANOVA with Bonferroni's correction was used to assess differences between three or more groups (e.g., the luciferase assay data). Each animal experiment, comprising a minimum of three mice, entailed quantification of 5–14 retinas. All experiments were conducted at least in triplicates, and prior to statistical analyses, all data were tested for normality and met the assumptions required for parametric testing.

## Author Contributions

M.X. and D.X. conceived and designed the research. J.L., M.X., D.X., Y.G., K.X., and S.W. performed the experiments and analysed the data. M.X. and J.L. interpreted the data and wrote the manuscript. All authors contributed to critical reading of the manuscript.

## Funding

This work was supported by the Ministry of Science and Technology of the People's Republic of China (2021ZD0202603), National Natural Science Foundation of China (32270864, 81970794), Science and Technology Program of Guangzhou (SL2024A03J01135), Science and Technology Planning Project of Guangdong Province (2023B1212060018), Fundamental Research Funds of the State Key Laboratory of Ophthalmology.

## Conflicts of Interest

The authors declare no conflicts of interest.

## Supporting information


**Table S1:** List of genes differentially expressed in E17.5 Zeb2CKO retinas as determined by RNA‐seq analysis

**Table S2:** List of genes differentially expressed in P17 OIR Zeb2CKO retinas as determined by RNA‐seq analysis

**Table S3:** Vascular markers and primary antibodies used in Western blotting, immunostaining, ChIP or CUT&Tag
**Table S4:** Secondary antibodies used in Western blotting or immunostaining
**Table S5:** Gene‐specific primer sequences used for qRT‐PCR analysis
**Table S6:** Gene‐specific primer sequences used for ChIP assay

**Figure S1:** Zeb2 is transiently expressed in astrocyte progenitor cells in developing mouse retinas. (A–D) Double‐immunofluorescence staining of wild‐type whole‐mount retinas for Pax2 (green) and Zeb2 (red) with DAPI counterlabeling (blue) at developmental stages E15.5, E17.5, and P0. The image shown in (B) is from the central region of a representative E17.5 retina and that in (C) is from the intermediate to peripheral region. Note that the Zeb2‐immunoreactive cells in (D) are retinal ganglion cells and amacrine cells located within the retinal ganglion cell layer. Abbreviation: ONH, optic nerve head. The insets in (B, C) show corresponding outlined regions at a higher magnification. Scale bar, 100 μm.
**Figure S2:** The retinal and eyeball sizes of control and Zeb2CKO mice. (A) PCR analysis of genomic DNA from Zeb2^fl/fl^, Zeb2^+/+^ and Zeb2^+/fl^ mice. The wild‐type and floxed (fl) alleles yield a product of 197 bp and 339 bp, respectively. (B) Retinal morphology of P16 control and Zeb2CKO mice. (C) The morphology of eyeballs (upper) and retinas (lower) of P25 control and Zeb2CKO animals. Scale bar: B, C, 1 mm.
**Figure S3:** The astrocytes and vasculature are normal in Zeb2CKO retinas after P12. (A) P12 control and Zeb2CKO flat‐mount retinas were triple‐immunostained for IB4, Pax2 and GFAP. Shown are representative confocal images in the central, intermediate and peripheral regions. (B‐D) Quantification of Pax2^+^ cells (B), GFAP^+^ area (C) and GFAP^+^/Pax2+ area ratio (D) in P12 control and Zeb2CKO retinas in the central, intermediate and peripheral regions. Data are presented as mean ± SEM (*n* = 6). (E) IB4 immunofluorescence (vitreal surface vasculature, blue; IPL vasculature, green; OPL vasculature, red) images of P16 control and Zeb2CKO flat‐mount retinas in the central, intermediate and peripheral regions. (F‐H) Quantification of IB4^+^ vascular area of P16 control and Zeb2CKO retinas in the central (F), intermediate (G) and peripheral (H) regions. Data are presented as mean ± SEM (*n* = 7). Abbreviations: IPL, inner plexiform layer; OPL, outer plexiform layer. Scale bar: E, 200 μm; A, 100 μm.
**Figure S4:** Increased endothelial cells in P0 Zeb2CKO retinas. (A) ERG immunofluorescence images of P0 control and Zeb2CKO flat‐mount retinas around the ONH (optic nerve head) area. (B) Quantification of all ERG^+^ cells in the region beyond 150 μm from the ONH (denoted in A) in P0 control and Zeb2CKO retinas. Data are presented as mean ± SEM (*n* = 5–6). **p < 0.01. Scale bar: A, 100 μm.
**Figure S5:** Altered transcriptome profiles detected by RNA‐seq analysis between E17.5 Zeb2CKO and control retinas. (A) Principal component (PC) analysis of RNA‐seq data showing that the Zeb2CKO retinal samples (red) are discriminated from the control samples (yellow). (B) Scatter plot of global gene expression profiles in CKO and control retinas. Gene expression levels are depicted in log10 scale. The diagonal line represents equal expression in the two genotypes. (C) Volcano plot (significance vs. fold change) of significantly downregulated (green) and upregulated (red) genes (fold change ≥ 1.5 and *p* < 0.05) between the CKO and control retinas. (D) Heatmap of differentially expressed genes (DEGs) reveals a large group of significantly upregulated genes as well as a smaller cluster of significantly downregulated ones in CKO retinas. (E) Top 16 enriched GO terms plus 4 representative GO terms associated with glial cell differentiation, FGF receptor signalling, or GDNF receptor signalling for the DEGs between the CKO and control retinas. (F) GSEA of the RNA‐seq data identifies enriched gene sets associated with endothelium development, endothelial cell differentiation, establishment of endothelial barrier, vasculogenesis, and epithelial cell development. (G) Network plot of 4 representative enriched angiogenesis‐related GO terms or gene sets (nodes) and their associated DEGs. Node size represents the gene‐set size. (H) Expression heatmap of a set of DEGs involved in angiogenesis, gliogenesis or FGF signalling. (I) qRT‐PCR assay of the RNA expression levels of the indicated genes involved in angiogenesis, gliogenesis or FGF signalling in P0 control and Zeb2CKO retinas. Data are presented as mean ± SEM (*n* = 3). *p < 0.05, **p < 0.01, ***p < 0.001.
**Figure S6:** Upregulation of *Vegfa* expression in P0 Zeb2CKO retinas. (A) Relative RNA expression levels of *Hif1a*, *Epas1 (Hif2a), Vegfa, Angpt1, Angpt2*, and *Fgf2* were determined by qRT‐PCR assay in P0 control and Zeb2CKO retinas. Data are presented as mean ± SEM (*n* = 3). **p < 0.01. (B) Western blot analysis of HIF2a and VEGFA protein levels in 3 each P0 control and Zeb2CKO retinas. GAPDH served as the internal protein control. (C, D) Quantification of HIF2a and VEGFA protein levels in P0 control and Zeb2CKO retinas. Data are presented as mean ± SEM (*n* = 3). *p < 0.01; ns, no significance. (E) Representative confocal images of HIF2a immunofluorescence and DAPI labeling of P0 control and Zeb2CKO retinal sections. Abbreviations: GCL, ganglion cell layer; NBL, neuroblastic layer. Scale bar: D, 50 μm.
**Figure S7:** Direct regulation of downstream gene expression by Zeb2. (A) Schematic of the *Vegfa* promoter region with the positions of the 4 putative Zeb2 binding motifs [5′‐CACCT(G)‐3′] indicated. The horizontal arrows indicate the positions of PCR primers used to amplify the precipitated DNA fragments (sites 1–4) in the chromatin immunoprecipitation (ChIP) assay. The negative control fragments (sites 3 and 4) without binding motifs are located within the intron region. Indicated also is the transcription start site (TSS). (B) In the ChIP assay, chromatin DNA was prepared from adult mouse retinas, immunoprecipitated by an anti‐Zeb2 antibody, and quantified by qRT‐PCR. Data are presented as mean ± SEM (*n* = 3). **p < 0.01, ****p < 0.0001; ns, no significance. (C) Schematic of the luciferase assay. A 2‐kb *Vegfa* promoter (P) fragment was inserted upstream of Luc (luciferase) in the pGL3‐Basic vector. The open reading frame of the Zeb2 transcription factor (TF) gene was inserted into the pCI expression vector. (D) Relative luciferase activities after cotransfection of the *Vegfa* reporter plasmid with the control (pCI) plasmid or the indicated increasing amount of Zeb2 expression plasmid in 293T cells. Histograms represent the mean ± SEM of triplicate assays in a single experiment. ***p < 0.001, ****p < 0.0001. (E) CUT&Tag analysis of E17.5 mouse retinas were conducted to map genomic sites bound by Zeb2. Shown are heatmaps of the Zeb2 and H3K27me3 CUT&Tag signals around the Zeb2 CUT&Tag peak region. Each row represents a 1‐kb region centered on the Zeb2 peak summit, sorted by Zeb2 or H3K27me3 signal enrichment. IgG serves as the negative control. (F) A top‐ranked Zeb2‐binding motif (*p* = 1e‐67) identified by de novo motif search in a 300‐bp window centered at the peak summit. (G) Genome browser views of Zeb2, H3K27me3, and IgG CUT&Tag signals at the *Vegfa, Cdkn1b, Cyp1b1*, and *Nkx6‐1* loci. The y axis represents the number of normalized reads.
**Figure S8:** Upregulation of Zeb2 expression in retinal astrocytes after hypoxic injury. (A) Pax2 and Zeb2 double‐immunofluorescence staining of flat‐mount retinas from wild‐type mice at P12 under the normoxia condition, or 8 (P12‐8h) and 24 (P13‐24h) hours post vaso‐obliteration under the OIR condition. The insets show the corresponding outlined regions at a higher magnification. (B) Pax2, Zeb2 and ERG triple‐immunofluorescence staining of flat‐mount retinas from wild‐type mice at 8 (P12‐8h) and 24 (P13‐24h) hours post vaso‐obliteration under the OIR condition. AVA, avascular area; OIR, oxygen‐induced retinopathy; ONH, optic nerve head; VA, vascular area. Scale bar: A, B, 100 μm.
**Figure S9:** GO enrichment, KEGG pathway enrichment and gene set enrichment analyses (GSEA) of the downregulated DEGs between P17 OIR Zeb2CKO and control retinas. (A, B) Top 20 enriched GO terms (A) and KEGG pathways (B) for the downregulated DEGs. (C) GSEA of the downregulated genes identifies enriched gene sets associated with inflammatory response, regulation of inflammatory response, positive regulation of inflammatory response, cell chemotaxis, and response to cytokine. (D) Network plot of the top 5 enriched GO terms and their associated downregulated DEGs.
**Figure S10:** Unaltered expression of HIF/VEGF pathway components in P17 Zeb2CKO retinas. (A) Relative RNA expression levels of *Hif1a, Epas1(Hif2a), Vegfa, Slc2a1(Glut1)*, and *Ldha* were determined by qRT‐PCR assay in P17 OIR control and Zeb2CKO retinas. Data are presented as mean ± SEM (*n* = 3). ns, no significance. (B) Western blot analysis of HIF2a and VEGFA protein levels in 3 each P17 OIR control and Zeb2CKO retinas. GAPDH served as the internal protein control. (C, D) Quantification of HIF2a (C) and VEGFA (D) protein levels in P17 OIR control and Zeb2CKO retinas. Data are presented as mean ± SEM (*n* = 3). ns, no significance. (E) Representative confocal images of HIF2a immunofluorescence and DAPI labeling of retinal sections from P17 control and Zeb2CKO animals under the normoxic and OIR conditions. GCL, ganglion cell layer; INL, inner nuclear layer; OIR, oxygen‐induced retinopathy; ONL, outer nuclear layer. Scale bar: E, 50 μm.
**Figure S11:** Assessment of the purity of the primary astrocytes (pACs) by immunofluorescence. (A–C) Representative confocal images of pACs immunostained for astrocyte‐specific protein markers GFAP (A), S100b (B) or Sox9 (C) and counterstained with DAPI. pACs were isolated from P2 pups of control mice. Scale bar: 20 μm. (D) Quantification of the proportions of marker‐positive pACs (GFAP^+^/DAPI^+^, S100b^+^/DAPI^+^ or Sox9^+^/DAPI^+^). Data are presented as mean ± SEM (*n* = 20).

## Data Availability

The data that support the findings of this study are openly available in SRA database at https://www.ncbi.nlm.nih.gov/sra/PRJNA1344440, reference number PRJNA1344440.
