# Supplementary material for: Zeb2 Controls Retinal Physiological and Pathological Angiogenesis by Regulating Astrocyte Proliferation and Differentiation
Source: Cell Prolif. 2026 May 26;59(10):e70236. doi: 10.1111/cpr.70236 (PMC13325830; doi:10.1111/cpr.70236)
Supplement: Supplementary file 1 [file CPR-59-e70236-s002.pdf]

**Table S2. List of genes differentially expressed in P17 OIR Zeb2 CKO retinas as determined by RNA-seq analysis**

| gene | baseMean | log2FoldChange | lfcSE | stat | pvalue | CKO1 | CKO2 | CKO3 | Control1 | Control2 | Control3 |
|---|---|---|---|---|---|---|---|---|---|---|---|
| Gm2026 | 247525118 | -6819875442 | 077934313 | -87507994 | 212E-18 | 0 | 290159236 | 100813846 | 473067778 | 466153721 | 532946233 |
| Gm18835 | 238171114 | -4760713788 | 16688738 | -28526506 | 000433563 | 0 | 0 | 0 | 7692159 | 308031093 | 351779692 |
| Gm30294 | 216243332 | -4651177374 | 170007321 | -27358689 | 000622158 | 0 | 0 | 0 | 2564053 | 513385155 | 527669538 |
| Crygn | 199691167 | -4508796249 | 174950705 | -2577181 | 000996098 | 0 | 0 | 0 | 64101325 | 205354062 | 351779692 |
| Gm36533 | 188070843 | -4438039151 | 192420677 | -23064253 | 002108688 | 0 | 0 | 0 | 5128106 | 0 | 615614461 |
| Gm3776 | 171614438 | -4306717045 | 180989263 | -23795428 | 001733413 | 0 | 0 | 0 | 38460795 | 205354062 | 439724615 |
| Rpl35a-ps2 | 144194141 | -4075020951 | 205279529 | -19851083 | 004713242 | 0 | 0 | 0 | 0 | 513385155 | 351779692 |
| Gm4131 | 146553733 | -4059810627 | 192956895 | -21039987 | 003537856 | 0 | 0 | 0 | 5128106 | 102677031 | 263834769 |
| Capn11 | 283392848 | -3863689743 | 053449502 | -7228673 | 488E-13 | 229832049 | 507778662 | 302441537 | 56409166 | 513385155 | 518875046 |
| Dcstamp | 256211667 | -3826738423 | 160702628 | -23812544 | 001725379 | 0 | 0 | 100813846 | 5128106 | 308031093 | 615614461 |
| Fmr1nb | 254868434 | -3784915734 | 163120302 | -23203217 | 002032348 | 114916024 | 0 | 0 | 7692159 | 205354062 | 439724615 |
| Mip | 637873379 | -37776044 | 118507302 | -31876554 | 000143431 | 0 | 217619427 | 0 | 192303975 | 13348014 | 351779692 |
| Crygs | 115572767 | -3775934618 | 027469626 | -13745854 | 539E-43 | 919328196 | 137825637 | 241953229 | 192303975 | 227943009 | 226018452 |
| Gm30601 | 246464882 | -3757180773 | 161498657 | -23264471 | 001999471 | 0 | 0 | 100813846 | 5128106 | 513385155 | 351779692 |
| Cryba1 | 187087872 | -3698923097 | 021483762 | -17217297 | 197E-66 | 160882434 | 319175159 | 302441537 | 32948081 | 335753892 | 379042618 |
| Fcrla | 222840786 | -3636344336 | 166269286 | -21870211 | 002874098 | 0 | 072539809 | 0 | 38460795 | 616062187 | 263834769 |
| Gm32092 | 220843504 | -36005636 | 166872459 | -2157674 | 003095319 | 0 | 0 | 100813846 | 2564053 | 616062187 | 351779692 |
| LOC11549034 | 529724971 | -34938852 | 118656996 | -29445253 | 000323451 | 0 | 217619427 | 0 | 15384318 | 718739218 | 703559384 |
| Gm34194 | 200817245 | -348301062 | 169289476 | -20574289 | 003964499 | 0 | 072539809 | 0 | 38460795 | 308031093 | 439724615 |
| Gja3 | 199018326 | -3474032208 | 170338473 | -20394877 | 004140138 | 0 | 072539809 | 0 | 2564053 | 513385155 | 351779692 |
| Oaz3 | 199018326 | -3474032208 | 170338473 | -20394877 | 004140138 | 0 | 072539809 | 0 | 2564053 | 513385155 | 351779692 |
| Cryaa | 591511486 | -3402987547 | 014053752 | -24214086 | 158E-129 | 735462557 | 103731927 | 127025445 | 112049116 | 116127722 | 962996907 |
| Crygd | 219531919 | -3341905027 | 018384726 | -18177616 | 776E-74 | 333256471 | 384460987 | 46374369 | 376915791 | 391199488 | 430930123 |
| E230016K23F | 513519043 | -3317233982 | 112351005 | -29525628 | 000315148 | 114916024 | 072539809 | 100813846 | 7692159 | 123212437 | 791504307 |
| Hao1 | 353562476 | -3307794266 | 136548094 | -2422439 | 001541671 | 114916024 | 0 | 100813846 | 5128106 | 513385155 | 87944923 |
| Crygb | 285134264 | -3293896566 | 016358182 | -2013608 | 356E-90 | 413697688 | 500524681 | 665371381 | 517938706 | 525706399 | 509201104 |
| Crybb2 | 138168213 | -328551273 | 022593022 | -14542157 | 655E-48 | 183865639 | 275651274 | 302441537 | 243585035 | 239237482 | 269990914 |
| S100a8 | 304982663 | -3186054047 | 141586438 | -22502537 | 002443285 | 0 | 072539809 | 100813846 | 5128106 | 616062187 | 527669538 |
| Crygc | 172175307 | -3160574483 | 019848032 | -15923868 | 433E-57 | 287290061 | 319175159 | 433499536 | 314096492 | 308031093 | 306927781 |
| Vmn2r2 | 281160203 | -3066584617 | 14463078 | -21202849 | 003398202 | 0 | 072539809 | 100813846 | 38460795 | 513385155 | 615614461 |

| | | | | | | | | | | | |
|---|---|---|---|---|---|---|---|---|---|---|---|
| Mmp3 | 407377146 | -3037886437 | 125617463 | -24183631 | 001559051 | 0 | 145079618 | 100813846 | 10256212 | 205354062 | 967394153 |
| Rpl31-ps9 | 443736069 | -3022973328 | 121046951 | -2497356 | 001251233 | 0 | 0 | 302441537 | 10256212 | 718739218 | 615614461 |
| Crygf | 103127693 | -3014156839 | 026723876 | -11278891 | 167E-29 | 114916024 | 217619427 | 342767075 | 178201683 | 203300522 | 169733701 |
| Gm51974 | 268853079 | -2950728604 | 145617859 | -20263508 | 004272885 | 114916024 | 072539809 | 0 | 38460795 | 513385155 | 527669538 |
| Gm4737 | 169522458 | -2949871087 | 019200999 | -15363113 | 289E-53 | 367731278 | 442492834 | 342767075 | 297430148 | 308031093 | 29637439 |
| Cryba2 | 348598309 | -2892892212 | 041768244 | -69260566 | 433E-12 | 574580122 | 797937898 | 11089523 | 48717007 | 62632989 | 729942861 |
| Gbp10 | 665655882 | -2815657348 | 097989638 | -28734236 | 000406049 | 0 | 435238853 | 0 | 115382385 | 143747844 | 967394153 |
| Cryga | 188542713 | -2779243833 | 019044194 | -14593654 | 308E-48 | 333256471 | 602080414 | 473825074 | 314096492 | 33062004 | 345623547 |
| Cryge | 280479908 | -2771846576 | 016227773 | -17080881 | 206E-65 | 505630508 | 819699841 | 79642938 | 473067778 | 468207262 | 529428436 |
| Dmp1 | 446422497 | -2767557558 | 113266771 | -24433976 | 00145497 | 0 | 217619427 | 100813846 | 5128106 | 616062187 | 123122892 |
| Cyp2b27-ps | 114431423 | -2720875296 | 070129809 | -387977 | 000010456 | 229832049 | 435238853 | 201627691 | 243585035 | 215621765 | 140711877 |
| Crybb1 | 550226866 | -2550124157 | 031675952 | -8050663 | 823E-16 | 172374037 | 116063694 | 201627691 | 935879345 | 995967202 | 87944923 |
| Cryba4 | 59131106 | -2533333242 | 030255473 | -83731404 | 561E-17 | 229832049 | 16684156 | 131057999 | 101280093 | 995967202 | 101136661 |
| Ifi209 | 516694181 | -2504324897 | 098514322 | -25420922 | 001101911 | 114916024 | 145079618 | 201627691 | 10256212 | 821416249 | 791504307 |
| Mgst2 | 416565915 | -2413934841 | 121111702 | -19931475 | 00462453 | 0 | 0 | 403255382 | 10256212 | 718739218 | 351779692 |
| Gm4951 | 472847846 | -2398204201 | 104949324 | -22851069 | 002230656 | 0 | 145079618 | 302441537 | 5128106 | 821416249 | 105533908 |
| Foxd2 | 440817814 | -2386040079 | 107838149 | -22126122 | 002692439 | 0 | 290159236 | 100813846 | 64101325 | 821416249 | 791504307 |
| Cd200r1 | 428875385 | -2224958658 | 107405485 | -20715503 | 00383074 | 0 | 145079618 | 302441537 | 5128106 | 821416249 | 791504307 |
| Asb14 | 51347023 | -2209909875 | 09680869 | -22827598 | 002244452 | 114916024 | 217619427 | 201627691 | 115382385 | 513385155 | 87944923 |
| Ccl7 | 186718196 | -2183891997 | 052797632 | -41363446 | 353E-05 | 574580122 | 65285828 | 806510765 | 23076477 | 246424875 | 439724615 |
| Clec4n | 133802359 | -2167470512 | 06048688 | -3583373 | 000033919 | 344748073 | 507778662 | 604883073 | 192303975 | 174550953 | 290218246 |
| Clec4d | 557519658 | -2157087776 | 095713847 | -22536841 | 002421605 | 229832049 | 362699044 | 0 | 64101325 | 123212437 | 87944923 |
| Zfp990 | 847452502 | -2099660728 | 075426854 | -27837045 | 00053742 | 114916024 | 507778662 | 302441537 | 115382385 | 13348014 | 167095354 |
| Mmp10 | 140625984 | -2068187977 | 05847355 | -35369633 | 000040476 | 229832049 | 65285828 | 705696919 | 243585035 | 195086359 | 246245784 |
| Iigp1 | 403291724 | -2058516622 | 033917148 | -60692504 | 129E-09 | 137899229 | 16684156 | 161302153 | 628192985 | 657132999 | 668381415 |
| Epsti1 | 512339319 | -2018210418 | 095246699 | -21189295 | 003409642 | 114916024 | 362699044 | 100813846 | 89741855 | 718739218 | 87944923 |
| LOC10086232 | 927541433 | -1936990709 | 097099941 | -19948423 | 004606008 | 689496147 | 362699044 | 100813846 | 2564053 | 184818656 | 0 |
| Gm37013 | 125458278 | -1921836102 | 074133922 | -25923842 | 000953133 | 0 | 435238853 | 11089523 | 346147155 | 102677031 | 149506369 |
| H2-Aa | 170978666 | -1906568767 | 054927735 | -34710493 | 000051843 | 229832049 | 123317675 | 604883073 | 269225565 | 277227984 | 272629261 |
| Mrc1 | 534752363 | -1886904813 | 029039335 | -64977549 | 815E-11 | 229832049 | 239381369 | 211709076 | 858957755 | 841951655 | 826682276 |
| Gm3317 | 942720764 | -1856256074 | 081764424 | -22702491 | 002319248 | 0 | 943017516 | 201627691 | 166663445 | 205354062 | 791504307 |
| Gm16174 | 616853023 | -1848837632 | 093555878 | -19761854 | 004813378 | 0 | 290159236 | 504069228 | 38460795 | 16428325 | 87944923 |
| 1500035N22I | 68960244 | -1823045456 | 027862756 | -65429474 | 603E-11 | 321764868 | 319175159 | 272197383 | 103844146 | 831683952 | 135435181 |

| | | | | | | | | | | | |
|---|---|---|---|---|---|---|---|---|---|---|---|
| Gm36538 | 670238216 | -1778759725 | 082550378 | -21547566 | 003118089 | 114916024 | 362699044 | 403255382 | 141022915 | 102677031 | 703559384 |
| Kcnj15 | 658057218 | -1766527311 | 081726542 | -21615099 | 003065597 | 114916024 | 362699044 | 403255382 | 89741855 | 102677031 | 1143284 |
| Cxcl10 | 328636168 | -1758701729 | 037667364 | -46690332 | 303E-06 | 114916024 | 188603503 | 141139384 | 423068745 | 523652859 | 580436492 |
| Ms4a7 | 821312666 | -173503421 | 075042685 | -23120631 | 002077421 | 114916024 | 580318471 | 403255382 | 141022915 | 92409328 | 149506369 |
| Oasl1 | 690311821 | -1710724893 | 082094516 | -2083848 | 0037174 | 459664098 | 217619427 | 302441537 | 166663445 | 616062187 | 87944923 |
| Gbp4 | 149699424 | -1692408892 | 056478073 | -29965769 | 000273029 | 459664098 | 108809713 | 504069228 | 269225565 | 256692578 | 167095354 |
| Gm16464 | 801609121 | -1656731476 | 073983967 | -22393115 | 002513566 | 344748073 | 580318471 | 201627691 | 141022915 | 112944734 | 1143284 |
| Crybb3 | 191013329 | -1655348972 | 016725123 | -98973799 | 427E-23 | 103424422 | 855969745 | 877080456 | 320506625 | 294683079 | 254160827 |
| Galnt12 | 12168317 | -1650168766 | 062391696 | -26448532 | 000817264 | 459664098 | 870477707 | 403255382 | 115382385 | 256692578 | 184684338 |
| Tmprss11e | 244315014 | -1647282012 | 047116947 | -34961561 | 000047201 | 804412171 | 203111465 | 604883073 | 346147155 | 441511234 | 334190707 |
| Taco1os | 746046741 | -1644325135 | 082670056 | -19890214 | 004669884 | 0 | 362699044 | 705696919 | 12820265 | 718739218 | 140711877 |
| Ch25h | 212780903 | -159858318 | 046789687 | -34165289 | 000063425 | 689496147 | 15958758 | 806510765 | 30768636 | 308031093 | 351779692 |
| Ccdc194 | 874701148 | -1595335925 | 072706544 | -21942123 | 002822015 | 689496147 | 435238853 | 201627691 | 7692159 | 174550953 | 140711877 |
| Pgf | 132427713 | -1583997831 | 0190123 | -83314371 | 799E-17 | 586071725 | 616588376 | 786347995 | 202560187 | 189952508 | 203152772 |
| Lrr1 | 856017825 | -1575750953 | 071617855 | -22002208 | 002779123 | 229832049 | 435238853 | 604883073 | 166663445 | 123212437 | 967394153 |
| Ccl2 | 697267188 | -1566124117 | 02600106 | -60233087 | 171E-09 | 287290061 | 391714968 | 373011229 | 910238815 | 101650261 | 120484544 |
| LOC10816864 | 668202942 | -1564133951 | 02577493 | -60684315 | 129E-09 | 344748073 | 333683121 | 33268569 | 111536305 | 985699498 | 897038215 |
| Timp1 | 15905427 | -1547241846 | 017389603 | -8897511 | 571E-19 | 850378581 | 848715764 | 735941073 | 220508558 | 22999655 | 260316972 |
| S100a3 | 945528102 | -1545623541 | 066585445 | -23212634 | 002027263 | 344748073 | 580318471 | 504069228 | 12820265 | 16428325 | 131917384 |
| Bcl3 | 166796084 | -150214497 | 019585517 | -76696724 | 172E-14 | 116065185 | 819699841 | 645208612 | 237174902 | 274147673 | 226897901 |
| Gm36841 | 163976487 | -1483422083 | 051346792 | -28890258 | 000386437 | 919328196 | 943017516 | 705696919 | 30768636 | 225889468 | 193478831 |
| Tnfrsf9 | 365667968 | -1475766355 | 035711064 | -41325186 | 359E-05 | 218340446 | 188603503 | 171383537 | 705114575 | 46204664 | 448519107 |
| 9330188P03F | 936249395 | -1458964605 | 022214316 | -65676773 | 511E-11 | 51712211 | 457000796 | 534313382 | 117946438 | 141694303 | 151265268 |
| Gm20482 | 187602677 | -144916921 | 061126816 | -23707585 | 001775162 | 114916024 | 181349522 | 0 | 269225565 | 287495687 | 272629261 |
| Gm19951 | 820241323 | -1445836692 | 07081487 | -20417134 | 004117997 | 459664098 | 362699044 | 504069228 | 141022915 | 112944734 | 105533908 |
| Angpt4 | 178239039 | -1431166401 | 050569097 | -28301206 | 000465305 | 103424422 | 580318471 | 131057999 | 30768636 | 266960281 | 202273323 |
| Hbb-bt | 510071818 | -1405418048 | 013620462 | -10318431 | 582E-25 | 209147165 | 293060828 | 334701967 | 687166204 | 747488786 | 788865959 |
| 1700030C10F | 3009158 | -1397919918 | 045103356 | -309937 | 000193933 | 459664098 | 268397293 | 171383537 | 346147155 | 472314343 | 501286061 |
| Lilr4b | 140863686 | -138670057 | 057016189 | -24321172 | 001501085 | 344748073 | 870477707 | 11089523 | 15384318 | 195086359 | 263834769 |
| Gm4070 | 774751269 | -1382550023 | 062578189 | -22093161 | 002715266 | 149390832 | 420730892 | 715778304 | 119228464 | 139640762 | 773915322 |
| Fgr | 321447444 | -1364333699 | 038213876 | -35702573 | 000035663 | 919328196 | 217619427 | 22179046 | 499990335 | 431243531 | 466108092 |
| Pf4 | 192437679 | -1363031477 | 047111645 | -28931944 | 000381345 | 137899229 | 116063694 | 705696919 | 243585035 | 287495687 | 299012738 |
| Ccbe1 | 111129303 | -1362670073 | 061971176 | -21988772 | 002788665 | 459664098 | 870477707 | 504069228 | 166663445 | 123212437 | 193478831 |

| | | | | | | | | | | | |
|---|---|---|---|---|---|---|---|---|---|---|---|
| Fam90a1b | 116547095 | -136164328 | 059098992 | -23040042 | 002122241 | 689496147 | 580318471 | 705696919 | 141022915 | 184818656 | 175889846 |
| A730094K22F | 203091785 | -1358155315 | 045682463 | -29730343 | 000294871 | 804412171 | 108809713 | 151220768 | 30768636 | 29776339 | 272629261 |
| Gm12866 | 107275412 | -1353968866 | 065020436 | -20823743 | 003730829 | 344748073 | 101555732 | 403255382 | 115382385 | 184818656 | 167095354 |
| Rsad2 | 246974331 | -1353462462 | 041524435 | -32594362 | 000111634 | 137899229 | 174095541 | 100813846 | 346147155 | 379905015 | 3429852 |
| Ifitm1 | 566372422 | -1352427589 | 029086364 | -46496964 | 332E-06 | 264306856 | 319175159 | 373011229 | 782036165 | 657132999 | 100257212 |
| Ifi204 | 30680662 | -1339330397 | 04126291 | -32458457 | 000117102 | 241323651 | 137825637 | 151220768 | 35896742 | 318298796 | 633203446 |
| Gm34292 | 155192422 | -1302787658 | 054776701 | -23783609 | 00173898 | 574580122 | 145079618 | 604883073 | 166663445 | 246424875 | 255040277 |
| Ifi211 | 289163184 | -130130516 | 040550238 | -32091184 | 000133143 | 137899229 | 16684156 | 191546307 | 5128106 | 46204664 | 263834769 |
| Enpp6 | 131871106 | -1296909308 | 05915919 | -21922364 | 002836244 | 344748073 | 725398089 | 120976615 | 192303975 | 13348014 | 237451292 |
| Msr1 | 350338415 | -129035699 | 034577809 | -37317488 | 000019016 | 229832049 | 210365446 | 171383537 | 474349805 | 46204664 | 554053015 |
| Tgm2 | 631640869 | -127851278 | 010066613 | -12700525 | 587E-37 | 396460284 | 32642914 | 387125167 | 902546656 | 894316941 | 882967027 |
| Gdf1 | 246724399 | -1278108404 | 045420573 | -28139416 | 000489381 | 689496147 | 130571656 | 231871845 | 28204583 | 441511234 | 325396215 |
| Ccl11 | 269467295 | -1276684259 | 043409006 | -29410585 | 000327093 | 149390832 | 116063694 | 211709076 | 38460795 | 236157172 | 518875046 |
| Kbtbd13 | 161583014 | -1264301879 | 053582074 | -23595613 | 001829656 | 919328196 | 130571656 | 604883073 | 141022915 | 308031093 | 237451292 |
| Isg15 | 305849216 | -1256160721 | 037512659 | -33486315 | 000081212 | 172374037 | 232127388 | 131057999 | 43588901 | 441511234 | 42213563 |
| Sp100 | 181525673 | -1256011843 | 049382566 | -25434317 | 001097695 | 114916024 | 870477707 | 120976615 | 320506625 | 16428325 | 281423754 |
| Ifi27l2a | 165460459 | -1244076609 | 053601927 | -2320955 | 002028927 | 459664098 | 116063694 | 131057999 | 141022915 | 277227984 | 281423754 |
| Gm43305 | 177256802 | -124175747 | 016006091 | -77580308 | 863E-15 | 976786208 | 110985908 | 106862676 | 242303008 | 239237482 | 266473117 |
| F13a1 | 393831938 | -1235919369 | 032711355 | -37782579 | 000015793 | 183865639 | 253889331 | 262115998 | 53845113 | 605794483 | 518875046 |
| Mfap4 | 119707811 | -1230824338 | 020052524 | -6138002 | 836E-10 | 884853388 | 660112261 | 614964458 | 162817365 | 159149398 | 180287092 |
| Rtp4 | 474672008 | -1218711148 | 029697794 | -41037093 | 407E-05 | 287290061 | 261143312 | 312522921 | 58973219 | 667400702 | 729942861 |
| Gm20742 | 452385019 | -1218107728 | 010804557 | -11274018 | 176E-29 | 266605177 | 264044904 | 286311321 | 624346905 | 603740943 | 669260864 |
| Gm12663 | 766980487 | -1209086649 | 024952758 | -48455031 | 126E-06 | 459664098 | 471508758 | 453662305 | 129484676 | 831683952 | 109051705 |
| Slit3 | 141713228 | -1199399537 | 060794348 | -197288 | 004850924 | 103424422 | 725398089 | 806510765 | 30768636 | 215621765 | 703559384 |
| Esm1 | 136490013 | -1190512879 | 018432049 | -64589284 | 105E-10 | 746954159 | 81244586 | 937568764 | 183329789 | 176604493 | 209308917 |
| Ccl28 | 540112565 | -118397845 | 02961781 | -39975219 | 640E-05 | 413697688 | 253889331 | 33268569 | 705114575 | 89329017 | 641997938 |
| Dnaja1-ps | 281091856 | -1173330579 | 038750241 | -3027931 | 000246234 | 206848844 | 181349522 | 131057999 | 346147155 | 451778937 | 369368677 |
| Gm21378 | 172315121 | -1164188249 | 050845282 | -22896682 | 002204056 | 160882434 | 101555732 | 604883073 | 20512424 | 215621765 | 290218246 |
| Gm40781 | 241313334 | -115694666 | 040838488 | -28329811 | 000461161 | 160882434 | 137825637 | 151220768 | 320506625 | 369637312 | 30780723 |
| Spidr | 612169624 | -1153891409 | 009654611 | -11951713 | 636E-33 | 361985477 | 399694347 | 376035644 | 810240748 | 836817803 | 888243722 |
| Serping1 | 702963501 | -1148968094 | 010246284 | -1121351 | 350E-29 | 37462624 | 475861146 | 455678582 | 101408296 | 972351484 | 92518059 |
| Mmp13 | 16449072 | -1142991136 | 050575448 | -22599724 | 002382297 | 574580122 | 116063694 | 131057999 | 23076477 | 205354062 | 246245784 |
| Mmp12 | 804605722 | -1142894911 | 024056039 | -47509689 | 202E-06 | 379222881 | 616588376 | 493987843 | 112818332 | 105757342 | 115207849 |

| | | | | | | | | | | | |
|---|---|---|---|---|---|---|---|---|---|---|---|
| Ms4a6d | 417573217 | -1135147916 | 031642475 | -35874182 | 000033397 | 229832049 | 304667197 | 241953229 | 64101325 | 533920562 | 554053015 |
| Tlr13 | 375904477 | -1131989348 | 033242469 | -34052505 | 000066103 | 183865639 | 24663535 | 272197383 | 551271395 | 482582046 | 518875046 |
| Ogn | 12627554 | -1120971722 | 018633814 | -60157933 | 179E-09 | 792920569 | 761667993 | 836754918 | 173073577 | 158122628 | 187322686 |
| Ifi207 | 183126242 | -1118977855 | 047138518 | -23738079 | 001760571 | 137899229 | 108809713 | 100813846 | 28204583 | 205354062 | 263834769 |
| Hbb-bs | 572669841 | -1115256812 | 01033168 | -10794534 | 365E-27 | 28211884 | 390771951 | 411824559 | 789856526 | 745845954 | 815601216 |
| Ifit1 | 582948178 | -1114018657 | 027252168 | -40878166 | 435E-05 | 356239676 | 369953025 | 383092613 | 653833515 | 811148546 | 923421691 |
| Bvht | 145560812 | -111256731 | 055048009 | -20210855 | 004327092 | 689496147 | 580318471 | 151220768 | 20512424 | 205354062 | 184684338 |
| Dio3 | 481813603 | -1111336427 | 030906955 | -35957487 | 000032346 | 402206086 | 261143312 | 262115998 | 576911925 | 605794483 | 782709815 |
| Mlkl | 247151075 | -1111209888 | 043002583 | -25840538 | 000976466 | 919328196 | 145079618 | 231871845 | 320506625 | 29776339 | 395752153 |
| Lyz2 | 321340458 | -1108165232 | 012279318 | -90246481 | 180E-19 | 18616396 | 213992436 | 209692799 | 424350771 | 443564774 | 450278006 |
| Pla1a | 445258677 | -109963134 | 033303375 | -33018615 | 000096045 | 229832049 | 232127388 | 393173998 | 46152954 | 739274624 | 615614461 |
| Nlrc5 | 364816352 | -1095751551 | 033449758 | -32758131 | 000105358 | 252815254 | 232127388 | 211709076 | 56409166 | 46204664 | 466108092 |
| Alas2 | 60562296 | -1087933528 | 026783106 | -4062014 | 487E-05 | 413697688 | 377207006 | 373011229 | 782036165 | 975431795 | 712353876 |
| Nox4 | 112404112 | -1082831584 | 020002172 | -54135699 | 618E-08 | 827395376 | 681874204 | 665371381 | 142304941 | 145801384 | 168854252 |
| C430049B03F | 340512071 | -1080198158 | 034316536 | -31477483 | 000164533 | 218340446 | 217619427 | 22179046 | 41024848 | 482582046 | 492491569 |
| Hba-a1 | 228449802 | -1078328956 | 025640706 | -42055354 | 260E-05 | 989426971 | 163432189 | 178037251 | 304224888 | 29776339 | 328298398 |
| Gm41608 | 680626541 | -1076936593 | 01149096 | -93720332 | 711E-21 | 360836317 | 454099204 | 497012259 | 874342073 | 886102778 | 101136661 |
| Gm39378 | 206260451 | -1070831515 | 04839878 | -22125176 | 002693092 | 689496147 | 145079618 | 181464922 | 33332689 | 174550953 | 334190707 |
| Xist | 333309536 | -1070081126 | 005149642 | -20779718 | 660E-96 | 222672781 | 211424527 | 211164681 | 441440185 | 44497145 | 468183592 |
| Ccr1 | 186846322 | -1068818407 | 049273689 | -21691463 | 003007158 | 689496147 | 145079618 | 141139384 | 33332689 | 256692578 | 175889846 |
| Hba-a2 | 156664614 | -1066988101 | 028215715 | -37815385 | 000015586 | 6561705 | 124550852 | 113415576 | 205637051 | 199296117 | 231471037 |
| Gm4792 | 100483857 | -1065512441 | 027492897 | -38755917 | 000010637 | 427487611 | 753688614 | 767193365 | 125638597 | 130810538 | 151617047 |
| A2m | 202666777 | -1057074588 | 006380269 | -16567868 | 119E-61 | 133072756 | 132602771 | 128940908 | 277302332 | 276817276 | 267264621 |
| Gm21028 | 146902967 | -1049751351 | 051796376 | -20266888 | 004269425 | 103424422 | 943017516 | 90732461 | 17948371 | 184818656 | 2286568 |
| Zfp981 | 338064243 | -1037269825 | 034752355 | -29847469 | 000283813 | 241323651 | 195857484 | 231871845 | 397428215 | 513385155 | 448519107 |
| Bmp2 | 338919428 | -1036375718 | 035201299 | -29441406 | 000323853 | 218340446 | 232127388 | 211709076 | 53845113 | 472314343 | 360574184 |
| Fgf2os | 501606428 | -1027849076 | 010838146 | -94836245 | 246E-21 | 314869907 | 334408519 | 341758936 | 624346905 | 651999147 | 74225515 |
| Vdr | 216446063 | -1027035559 | 043164911 | -23793297 | 001734415 | 137899229 | 130571656 | 161302153 | 269225565 | 256692578 | 3429852 |
| Bfsp2 | 44443488 | -1026877555 | 030821874 | -33316519 | 000086332 | 229832049 | 282905255 | 362929844 | 628192985 | 564723671 | 598025476 |
| Cldn2 | 600991087 | -1024541031 | 026386389 | -38828392 | 000010324 | 367731278 | 442492834 | 373011229 | 833317225 | 718739218 | 870654738 |
| Fgf23 | 482247241 | -1024534712 | 029094347 | -35214219 | 000042924 | 287290061 | 340937102 | 322604306 | 653833515 | 585259077 | 703559384 |
| Gpihbp1 | 25754841 | -1022331772 | 041646362 | -24547925 | 001409659 | 137899229 | 203111465 | 161302153 | 43588901 | 369637312 | 237451292 |
| Lilrb4a | 839771217 | -1018161349 | 022612339 | -45026804 | 671E-06 | 4711557 | 602080414 | 584720304 | 116664411 | 102677031 | 118725646 |

| | | | | | | | | | | | |
|---|---|---|---|---|---|---|---|---|---|---|---|
| Baat | 161204617 | -1014586216 | 051384572 | -19744958 | 00483254 | 574580122 | 108809713 | 151220768 | 243585035 | 195086359 | 211067815 |
| Hmox1 | 195140565 | -1008506349 | 008041268 | -12541633 | 442E-36 | 114226528 | 14058215 | 133376718 | 263584648 | 252688174 | 266385172 |
| Casp12 | 368223507 | -1006666955 | 0346345 | -29065439 | 000365446 | 183865639 | 224873408 | 322604306 | 551271395 | 513385155 | 413341138 |
| Edn2 | 797772031 | -0988085534 | 008378351 | -11793317 | 423E-32 | 532061193 | 53897078 | 532297105 | 107946631 | 107400175 | 102983505 |
| Tfpi2 | 290465564 | -0979332664 | 039832784 | -24586096 | 001394762 | 206848844 | 210365446 | 171383537 | 243585035 | 523652859 | 386957661 |
| Csf2rb | 187410562 | -0978924573 | 046694751 | -20964339 | 003604372 | 804412171 | 152333599 | 141139384 | 2564053 | 256692578 | 237451292 |
| Antxr2 | 854950403 | -0975358842 | 008114653 | -12019724 | 280E-33 | 579176763 | 566535907 | 584720304 | 112433724 | 113252765 | 114240455 |
| Pcolce | 126433491 | -0972514072 | 007483191 | -12995982 | 129E-38 | 825097056 | 866125318 | 866999072 | 17563763 | 162332386 | 164808786 |
| Gm46054 | 605398814 | -0971711961 | 02641872 | -3678119 | 000023496 | 390714483 | 362699044 | 473825074 | 884598285 | 790613139 | 729942861 |
| Serpina3n | 266806788 | -0969747308 | 00678983 | -14282351 | 282E-46 | 166628235 | 185194132 | 18902596 | 346916371 | 348280489 | 364795541 |
| Eif4ebp1 | 137275257 | -0967319416 | 017867265 | -54139199 | 617E-08 | 861870183 | 103731927 | 877080456 | 17563763 | 190979278 | 179407643 |
| Ccl12 | 385686843 | -0958183791 | 035198212 | -27222513 | 000648388 | 160882434 | 239381369 | 383092613 | 551271395 | 513385155 | 466108092 |
| Mlf1 | 720174523 | -0956560888 | 011791262 | -81124558 | 496E-16 | 390714483 | 519385032 | 557500566 | 87177802 | 976458566 | 100521047 |
| Lcat | 968974968 | -0953973774 | 022184235 | -43002329 | 171E-05 | 540105315 | 710890127 | 725859688 | 115382385 | 117051815 | 151265268 |
| Gm17501 | 183296038 | -0952714414 | 047167111 | -20198702 | 004339686 | 804412171 | 130571656 | 161302153 | 243585035 | 246424875 | 237451292 |
| Igfbp3 | 709383038 | -0948912044 | 00912314 | -10401156 | 245E-25 | 449321656 | 496897691 | 504069228 | 964083928 | 897397252 | 944528473 |
| Depp1 | 799590124 | -0948501875 | 025943807 | -36559857 | 00002562 | 333256471 | 703636146 | 584720304 | 99998067 | 106784112 | 110810603 |
| Tagln | 237054999 | -0946912749 | 014995186 | -63147783 | 271E-10 | 132153428 | 179898726 | 171383537 | 328198784 | 290575998 | 32011952 |
| Btla | 209878194 | -0946423539 | 044402805 | -21314499 | 00330521 | 114916024 | 145079618 | 171383537 | 217944505 | 266960281 | 3429852 |
| Egr2 | 678604309 | -0946369095 | 024779181 | -38192105 | 000013388 | 413697688 | 471508758 | 504069228 | 833317225 | 995967202 | 853065753 |
| Kcne4 | 331994706 | -0944371883 | 035610433 | -26519528 | 000800277 | 183865639 | 195857484 | 302441537 | 43588901 | 451778937 | 42213563 |
| Ackr3 | 166209092 | -093125282 | 016269094 | -57240607 | 104E-08 | 105722743 | 114612898 | 121984753 | 22691869 | 224862698 | 203152772 |
| Ncapg | 285778486 | -0928756851 | 038981213 | -23825756 | 0017192 | 114916024 | 24663535 | 22179046 | 371787685 | 390172718 | 369368677 |
| Cd72 | 296695423 | -0926606732 | 036434547 | -25432091 | 001098395 | 229832049 | 203111465 | 181464922 | 397428215 | 390172718 | 378163169 |
| Bcl2a1b | 332289383 | -0922273516 | 035357128 | -26084515 | 000909529 | 252815254 | 195857484 | 241953229 | 5128106 | 359369609 | 430930123 |
| Kif14 | 185057501 | -0916385133 | 015842357 | -57843988 | 728E-09 | 134451749 | 134198646 | 115935922 | 241020982 | 221782387 | 26295532 |
| Tnfaip2 | 358606399 | -0915268128 | 035212548 | -2599267 | 000934231 | 310273266 | 188603503 | 252034614 | 48717007 | 544188265 | 369368677 |
| Bub1b | 531941192 | -0914541539 | 010825888 | -84477276 | 297E-17 | 366582118 | 356170462 | 38510889 | 685884177 | 62838343 | 769518076 |
| Cela1 | 601168808 | -0914194236 | 027126046 | -33701713 | 000075121 | 333256471 | 500524681 | 403255382 | 884598285 | 790613139 | 694764892 |
| Gm13821 | 368348451 | -0912785685 | 011840725 | -77088668 | 127E-14 | 287290061 | 238655971 | 242961368 | 475631831 | 489769438 | 475782033 |
| Mndal | 375220292 | -0910883989 | 032975104 | -27623385 | 000573889 | 218340446 | 275651274 | 282278768 | 551271395 | 492849749 | 430930123 |
| Rps27a-ps3 | 215678675 | -0909113401 | 045129438 | -20144576 | 004396151 | 804412171 | 210365446 | 151220768 | 294866095 | 266960281 | 290218246 |
| Gm42788 | 224539625 | -0908355343 | 045024916 | -20174504 | 004364853 | 919328196 | 181349522 | 191546307 | 30768636 | 205354062 | 369368677 |

| | | | | | | | | | | | |
|---|---|---|---|---|---|---|---|---|---|---|---|
| Litaf | 314719227 | -090575529 | 012097225 | -74872982 | 703E-14 | 23327953 | 213992436 | 210700937 | 41024848 | 413788435 | 406305544 |
| Slc16a4 | 480482758 | -0905563617 | 030069688 | -30115498 | 000259918 | 367731278 | 391714968 | 241953229 | 58973219 | 605794483 | 685970399 |
| 2900057B20F | 14940951 | -090278663 | 01755683 | -51420821 | 272E-07 | 850378581 | 1138875 | 111903369 | 199996134 | 193032818 | 192599381 |
| Lad1 | 394699431 | -0898928754 | 011755454 | -76469081 | 206E-14 | 294185023 | 270573487 | 262115998 | 521784785 | 553429198 | 466108092 |
| Trim30a | 538985416 | -089587518 | 028504171 | -31429617 | 000167248 | 459664098 | 391714968 | 282278768 | 679474045 | 646865296 | 773915322 |
| Pbk | 259529615 | -0894697785 | 040369971 | -22162458 | 002667467 | 229832049 | 130571656 | 191546307 | 320506625 | 29776339 | 386957661 |
| Calca | 376236175 | -0894391899 | 033208851 | -26932335 | 000707627 | 241323651 | 253889331 | 292360152 | 576911925 | 400440421 | 492491569 |
| Srgn | 141786691 | -0885146203 | 018116185 | -48859415 | 103E-06 | 896344991 | 104457325 | 104846399 | 158971286 | 187898967 | 204911671 |
| Spp1 | 70089236 | -0884507973 | 012676543 | -69775173 | 300E-12 | 37462624 | 544773965 | 555484289 | 869213967 | 872754764 | 988500934 |
| Adgrl4 | 94220873 | -0884165884 | 008399917 | -10525888 | 656E-26 | 710181031 | 621666162 | 657306273 | 125895002 | 121261574 | 119253316 |
| Lcp2 | 104058865 | -0879049317 | 020169051 | -43584069 | 131E-05 | 666512942 | 725398089 | 806510765 | 135894809 | 125265978 | 143350224 |
| Nlrp5-ps | 317867927 | -0877243145 | 035814321 | -244942 | 001430865 | 218340446 | 261143312 | 191546307 | 346147155 | 441511234 | 448519107 |
| 5730507C01F | 291602095 | -0876940045 | 037899542 | -23138539 | 002067574 | 229832049 | 152333599 | 241953229 | 320506625 | 400440421 | 404546646 |
| Cebpd | 148020514 | -0874544709 | 007458317 | -11725765 | 940E-32 | 104458666 | 101338113 | 107568373 | 204226821 | 187693613 | 182837495 |
| Slc13a4 | 155384889 | -0865070632 | 017117292 | -50537822 | 433E-07 | 109170223 | 108809713 | 111903369 | 22691869 | 177631264 | 197876077 |
| Socs3 | 446251297 | -0861454339 | 010604655 | -81233602 | 453E-16 | 299930824 | 321351353 | 327644998 | 605116508 | 56061659 | 562847507 |
| Optc | 813472675 | -0856066482 | 011020206 | -77681529 | 796E-15 | 463111579 | 645604299 | 624037704 | 103972349 | 104422541 | 106413357 |
| Ube2l6 | 22762722 | -0855651464 | 014438781 | -59260643 | 310E-09 | 165479075 | 166116162 | 155253322 | 258969353 | 312138175 | 30780723 |
| Abi3bp | 104733093 | -0853179952 | 020653443 | -41309332 | 361E-05 | 827395376 | 631096337 | 79642938 | 125638597 | 130399829 | 146868021 |
| Slc16a12 | 635137766 | -0850219703 | 026374001 | -32237039 | 000126544 | 402206086 | 384460987 | 57463892 | 87177802 | 821416249 | 756326338 |
| Cxcl1 | 263342633 | -0848657 | 040603927 | -2090086 | 003661008 | 183865639 | 195857484 | 181464922 | 35896742 | 431243531 | 2286568 |
| Kcne3 | 383512276 | -084687632 | 032787707 | -2582908 | 000979714 | 241323651 | 311921178 | 262115998 | 53845113 | 533920562 | 413341138 |
| B2m | 147352423 | -0846860579 | 011804566 | -71740088 | 728E-13 | 814754613 | 112436704 | 121783125 | 18140675 | 176296462 | 210716035 |
| Klhl6 | 683732879 | -0840928707 | 024396891 | -34468683 | 000056712 | 51712211 | 435238853 | 524231997 | 858957755 | 852219358 | 914627199 |
| Gm13136 | 280490535 | -0838020079 | 038407013 | -21819455 | 002911356 | 149390832 | 217619427 | 231871845 | 38460795 | 400440421 | 299012738 |
| Fgf2 | 331814276 | -0833189557 | 006732317 | -1237597 | 353E-35 | 259940047 | 231329451 | 224915689 | 419991881 | 425185586 | 429523004 |
| Angpt2 | 591059956 | -0832434639 | 009409465 | -88467799 | 901E-19 | 417145169 | 430886465 | 427450705 | 723062946 | 784452518 | 763361932 |
| Bmp4 | 526129334 | -0831424383 | 027352018 | -30397186 | 000236799 | 390714483 | 391714968 | 352848459 | 66665378 | 677668405 | 677175907 |
| Gbp3 | 924853392 | -0829853233 | 022853904 | -36311223 | 000028219 | 540105315 | 65285828 | 806510765 | 110254279 | 105757342 | 138952978 |
| Tfpi | 240053312 | -0828233869 | 014335434 | -57775292 | 758E-09 | 160882434 | 158862181 | 199611414 | 310250413 | 315218485 | 295494941 |
| Lox | 551275808 | -0827953233 | 027050708 | -30607452 | 000220787 | 390714483 | 413476911 | 383092613 | 79485643 | 657132999 | 668381415 |
| Ggta1 | 121235092 | -0827295979 | 020984787 | -39423605 | 807E-05 | 632038135 | 884985669 | 109887092 | 155125206 | 142721073 | 167974803 |
| Adgre1 | 688530384 | -0825290821 | 024914649 | -33124722 | 000092475 | 586071725 | 435238853 | 473825074 | 961519875 | 883022467 | 791504307 |

| | | | | | | | | | | | |
|---|---|---|---|---|---|---|---|---|---|---|---|
| Gpx3 | 210702863 | -0823962661 | 014903189 | -55287673 | 322E-08 | 145943351 | 152333599 | 158277738 | 267943538 | 243344564 | 29637439 |
| Ctla2b | 47032846 | -0823421331 | 03349428 | -24583939 | 0013956 | 172374037 | 377207006 | 46374369 | 525630865 | 667400702 | 615614461 |
| Rab38 | 635470267 | -0817548424 | 026664657 | -30660377 | 000216916 | 459664098 | 435238853 | 493987843 | 64101325 | 780345436 | 100257212 |
| Lenep | 403710484 | -0815971863 | 034077494 | -23944597 | 001664487 | 172374037 | 391714968 | 302441537 | 499990335 | 554455968 | 501286061 |
| Zc3hav1 | 723299841 | -081344608 | 023624614 | -34432143 | 000057484 | 4711557 | 536794586 | 564557535 | 884598285 | 92409328 | 958599661 |
| Depdc1a | 284399871 | -0810730821 | 039882772 | -20327845 | 004207429 | 149390832 | 290159236 | 171383537 | 346147155 | 441511234 | 30780723 |
| Myct1 | 585968665 | -0808337085 | 026107369 | -30962028 | 000196016 | 436680893 | 435238853 | 403255382 | 79485643 | 75981003 | 685970399 |
| Il13ra1 | 126429376 | -0807424418 | 018297749 | -44126981 | 102E-05 | 965294605 | 964779458 | 826673534 | 162817365 | 152988776 | 167095354 |
| P2rx7 | 467292667 | -0807242401 | 030605599 | -26375645 | 000835037 | 241323651 | 340937102 | 433499536 | 628192985 | 544188265 | 615614461 |
| Ltbp1 | 271658518 | -0801910808 | 01312812 | -61083448 | 101E-09 | 212594645 | 191505095 | 190538168 | 365377552 | 323432648 | 346502997 |
| Rarres2 | 164838827 | -0800871932 | 016464826 | -48641384 | 115E-06 | 126407627 | 118239889 | 115935922 | 229482743 | 187898967 | 211067815 |
| Gm42048 | 755336719 | -0799455971 | 008549376 | -93510448 | 868E-21 | 567685161 | 537519984 | 550443597 | 94485353 | 945655456 | 985862587 |
| Apold1 | 148046097 | -0798152449 | 0174111 | -45841585 | 456E-06 | 101126102 | 118965287 | 103838261 | 170509524 | 188925737 | 204911671 |
| Cep55 | 328651471 | -0797626665 | 035883309 | -22228348 | 002622694 | 172374037 | 290159236 | 252034614 | 423068745 | 359369609 | 474902584 |
| Accsl | 26369658 | -0796910164 | 040494695 | -19679372 | 004907527 | 241323651 | 123317675 | 22179046 | 294866095 | 349101906 | 351779692 |
| Crhbp | 42825361 | -0796054975 | 011282849 | -70554429 | 172E-12 | 288439221 | 324252946 | 323612444 | 592296243 | 530840251 | 510080553 |
| Nupr1 | 483364176 | -0795922371 | 029651754 | -26842337 | 000726963 | 222937087 | 426534076 | 409304213 | 656397568 | 542134724 | 642877387 |
| Fcgr2b | 546571767 | -0795619688 | 027849363 | -28568685 | 000427843 | 448172495 | 355445064 | 403255382 | 576911925 | 800880842 | 694764892 |
| Gm17330 | 357531922 | -0791951608 | 035135516 | -22539917 | 002419669 | 206848844 | 348191083 | 22179046 | 48717007 | 379905015 | 501286061 |
| Nudt6 | 421003836 | -0791317754 | 012506467 | -63272688 | 250E-10 | 364283798 | 279278264 | 285303183 | 496144255 | 526733169 | 574280347 |
| Trim30d | 302914294 | -0790470158 | 035950187 | -21987929 | 002789265 | 241323651 | 224873408 | 201627691 | 346147155 | 390172718 | 413341138 |
| Olfml2a | 331509971 | -0789626181 | 039125818 | -20181717 | 004357339 | 356239676 | 181349522 | 201627691 | 28204583 | 554455968 | 413341138 |
| Niban1 | 256433954 | -078814433 | 013434593 | -58665291 | 445E-09 | 179268998 | 203836863 | 179448645 | 326916757 | 337807432 | 311325027 |
| Zfp951 | 143256724 | -078787113 | 017957728 | -43873652 | 115E-05 | 101126102 | 108809713 | 105854538 | 157689259 | 177631264 | 208429467 |
| Ctla2a | 479447701 | -0782499592 | 011832186 | -66133138 | 376E-11 | 299930824 | 389538774 | 365954259 | 555117474 | 647892066 | 618252809 |
| Prss56 | 101527034 | -0782497051 | 008568263 | -91325048 | 669E-20 | 79636805 | 761667993 | 680493458 | 133971769 | 12865432 | 122683168 |
| Gm5954 | 355575434 | -0779942296 | 0342108 | -22798131 | 002261878 | 333256471 | 224873408 | 231871845 | 48717007 | 390172718 | 466108092 |
| Zic4 | 497030645 | -0774491916 | 030272264 | -25584208 | 001051488 | 241323651 | 457000796 | 393173998 | 576911925 | 636597593 | 677175907 |
| Tm4sf1 | 521355859 | -0774353598 | 012504046 | -61928244 | 591E-10 | 310273266 | 430886465 | 409304213 | 699986469 | 59347324 | 684211501 |
| Ggt5 | 423003536 | -0773444144 | 033692053 | -22956278 | 002169717 | 195357242 | 435238853 | 292360152 | 576911925 | 554455968 | 483697076 |
| 4930523C07F | 685330919 | -0772061323 | 025472005 | -3031019 | 00024373 | 494138905 | 580318471 | 443580921 | 74357537 | 821416249 | 10289556 |
| Emp3 | 79082245 | -0769688626 | 025807754 | -29823929 | 000286005 | 367731278 | 602080414 | 776266611 | 103844146 | 975431795 | 984983137 |
| Gstm2 | 313211784 | -0766954669 | 03883546 | -19748824 | 004828149 | 229832049 | 195857484 | 272197383 | 46152954 | 236157172 | 483697076 |

| | | | | | | | | | | | |
|---|---|---|---|---|---|---|---|---|---|---|---|
| H2-Q7 | 124949056 | -0766950152 | 018352947 | -41788936 | 293E-05 | 953803003 | 943017516 | 877080456 | 164099392 | 143747844 | 164457006 |
| Rbp1 | 179652261 | -0766279667 | 010600427 | -72287625 | 487E-13 | 104688498 | 150302484 | 14365973 | 218457316 | 226608208 | 23419733 |
| Ccr5 | 526942591 | -0764616542 | 029365525 | -26037898 | 000921993 | 275798459 | 478762739 | 403255382 | 7692159 | 636597593 | 598025476 |
| Fbln1 | 38436369 | -0763065916 | 011413285 | -66857694 | 230E-11 | 257411895 | 298138615 | 297400844 | 494862229 | 488742668 | 469625889 |
| S100a11 | 486772578 | -0761587311 | 015113044 | -50392715 | 467E-07 | 248218613 | 422907086 | 409304213 | 593578269 | 616062187 | 630565098 |
| Ctsc | 13863187 | -0760711782 | 007838129 | -97052721 | 286E-22 | 942311401 | 108084315 | 106156979 | 170124917 | 171059934 | 182133936 |
| Gm15983 | 980824494 | -0760607983 | 021042073 | -36147008 | 000030069 | 60905493 | 819699841 | 746022457 | 123074544 | 116025045 | 131917384 |
| Tyrobp | 140863149 | -0758650182 | 021985013 | -34507607 | 000055901 | 632038135 | 124043073 | 125009169 | 176919657 | 16428325 | 191719932 |
| Icam1 | 525122988 | -0758482247 | 028185966 | -26909925 | 000712398 | 298781664 | 449746815 | 413336767 | 679474045 | 667400702 | 641997938 |
| Sdc1 | 66450313 | -0756204188 | 02496534 | -30290162 | 000245352 | 425189291 | 558556529 | 493987843 | 782036165 | 821416249 | 905832707 |
| Bub1 | 41818876 | -0751849654 | 030959484 | -24284954 | 001516162 | 367731278 | 297413216 | 272197383 | 551271395 | 492849749 | 527669538 |
| Gm13707 | 476522403 | -0751320775 | 031960717 | -23507632 | 001873495 | 448172495 | 224873408 | 403255382 | 653833515 | 513385155 | 615614461 |
| Lgals1 | 470070414 | -0750221852 | 014685747 | -51085033 | 325E-07 | 251666094 | 409124522 | 388133305 | 546143289 | 590392929 | 634962344 |
| Ptprc | 619515605 | -07469337 | 025291625 | -29532847 | 000314412 | 459664098 | 471508758 | 453662305 | 82049696 | 790613139 | 721148369 |
| Tmem267 | 763302914 | -0746890494 | 009240103 | -80831405 | 631E-16 | 510227149 | 606432802 | 590769135 | 935879345 | 955923159 | 980585891 |
| Gadd45b | 611981816 | -0745567812 | 009727532 | -76645118 | 180E-14 | 435531733 | 444669029 | 492979705 | 728191052 | 764943882 | 805575495 |
| Usp18 | 315879151 | -0744294012 | 036815627 | -20216796 | 004320946 | 321764868 | 181349522 | 211709076 | 397428215 | 431243531 | 351779692 |
| Myl9 | 404366621 | -0743912903 | 014657929 | -5075157 | 387E-07 | 220638767 | 333683121 | 350832183 | 471785752 | 509278074 | 539981827 |
| Lpxn | 442177373 | -0743313653 | 032413096 | -2293251 | 002183356 | 195357242 | 384460987 | 403255382 | 602552455 | 513385155 | 554053015 |
| Phldb2 | 151658362 | -0740957318 | 016595571 | -44647894 | 801E-06 | 109170223 | 114612898 | 116944061 | 17948371 | 1971399 | 192599381 |
| Icam2 | 910019733 | -0738933358 | 021131591 | -34968183 | 000047084 | 643529737 | 739906051 | 655289996 | 117946438 | 109864423 | 1143284 |
| Aoc3 | 580480102 | -073861564 | 026553043 | -2781661 | 000540815 | 436680893 | 377207006 | 493987843 | 782036165 | 698203811 | 694764892 |
| Zfp968-ps | 688309493 | -0737747286 | 031726369 | -23253442 | 002005356 | 344748073 | 493270701 | 715778304 | 551271395 | 811148546 | 121363994 |
| Ecscr | 198617562 | -0737327542 | 017199718 | -42868582 | 181E-05 | 108021063 | 165390764 | 171383537 | 255123273 | 240264253 | 25152248 |
| Tnnt2 | 947216276 | -0734778683 | 02200437 | -33392397 | 000084008 | 689496147 | 696382165 | 756103842 | 987160405 | 112944734 | 142470775 |
| Acta2 | 514732232 | -0733958197 | 011205541 | -65499576 | 576E-11 | 333256471 | 383010191 | 442572782 | 632039064 | 651999147 | 645515735 |
| H2-M3 | 389215234 | -0732596084 | 033704684 | -21735735 | 002973718 | 218340446 | 355445064 | 292360152 | 602552455 | 46204664 | 404546646 |
| Gm8251 | 624887799 | -0732436163 | 02607826 | -28086083 | 000497561 | 448172495 | 48601672 | 473825074 | 807676695 | 636597593 | 897038215 |
| Mgp | 1221531 | -0728085024 | 011034479 | -65982727 | 416E-11 | 722821794 | 99452078 | 103737447 | 156150828 | 156890504 | 144405564 |
| Pqlc3 | 312241383 | -0726985729 | 036757289 | -19778002 | 004795125 | 310273266 | 188603503 | 211709076 | 397428215 | 431243531 | 334190707 |
| Lrrc2 | 167223485 | -0725661767 | 006771286 | -1071675 | 849E-27 | 121351322 | 127815143 | 128840095 | 206406266 | 205046031 | 213882053 |
| Clec14a | 217527653 | -0722804384 | 014207545 | -50874687 | 363E-07 | 167777396 | 157411385 | 168359122 | 253841247 | 283388606 | 27438816 |
| Adgrf5 | 350335098 | -0718603116 | 011580775 | -62051384 | 546E-10 | 250516933 | 267671895 | 276229937 | 425632798 | 42816322 | 453795803 |

| | | | | | | | | | | | |
|---|---|---|---|---|---|---|---|---|---|---|---|
| Areg | 365943766 | -0714822193 | 033144627 | -21566759 | 003103092 | 310273266 | 290159236 | 231871845 | 41024848 | 513385155 | 439724615 |
| Tmem45a | 324710962 | -0714025678 | 035735039 | -19981108 | 004570465 | 252815254 | 203111465 | 282278768 | 48717007 | 379905015 | 3429852 |
| Samd9l | 167477952 | -0712351721 | 016829109 | -42328547 | 231E-05 | 116065185 | 11751449 | 148196353 | 192303975 | 207407603 | 223380104 |
| Serpine1 | 198631719 | -0710495605 | 015537806 | -45726893 | 482E-06 | 132153428 | 169743153 | 148196353 | 257687326 | 228969779 | 255040277 |
| Anxa2 | 137709904 | -071005993 | 007941027 | -89416637 | 383E-19 | 946908042 | 108084315 | 110491975 | 166791648 | 168903716 | 177296965 |
| Bpifb6 | 823103786 | -0709360295 | 022424822 | -31632818 | 000156001 | 666512942 | 565810509 | 645208612 | 111536305 | 934360983 | 101136661 |
| Ifit3 | 229998563 | -0708626406 | 013869974 | -5109068 | 324E-07 | 170075716 | 177722532 | 17642423 | 267943538 | 284415376 | 303409984 |
| Tlr2 | 589388763 | -0708290439 | 026770435 | -26457936 | 000814995 | 367731278 | 420730892 | 554476151 | 705114575 | 749542327 | 738737353 |
| Fxyd5 | 166527042 | -0705970229 | 018801111 | -37549389 | 000017338 | 942311401 | 14435422 | 140131245 | 187175869 | 194059589 | 239210191 |
| Col3a1 | 259145984 | -0703933078 | 013965657 | -5040458 | 464E-07 | 216042126 | 171919347 | 205660245 | 311532439 | 341914514 | 30780723 |
| Gng11 | 362719075 | -0702697642 | 012410844 | -56619652 | 150E-08 | 263157696 | 29596242 | 269172968 | 392300109 | 476421424 | 47929983 |
| Cfi | 785336621 | -0702610048 | 023181056 | -30309665 | 000243772 | 56308852 | 674620223 | 544394766 | 108972252 | 934360983 | 905832707 |
| Gbp2 | 127360336 | -070237349 | 017862339 | -39321472 | 842E-05 | 976786208 | 993795382 | 937568764 | 15384318 | 161202939 | 158300861 |
| Psmb8 | 770618602 | -0701771312 | 026521805 | -26460164 | 000814459 | 402206086 | 616588376 | 735941073 | 101280093 | 739274624 | 111690052 |
| Agpat2 | 42190765 | -0700839314 | 031923146 | -21953955 | 002813524 | 229832049 | 348191083 | 383092613 | 499990335 | 472314343 | 598025476 |
| Tmem252 | 831600913 | -0699625193 | 024898608 | -28098968 | 000495574 | 425189291 | 718144108 | 746022457 | 112818332 | 872754764 | 109931154 |
| Hpgds | 42493075 | -0696819959 | 031140417 | -22376706 | 002524254 | 287290061 | 348191083 | 33268569 | 525630865 | 616062187 | 439724615 |
| Scube1 | 98931116 | -0694846109 | 021327685 | -32579537 | 000112219 | 850378581 | 674620223 | 746022457 | 12820265 | 134506911 | 103775009 |
| Btc | 474561144 | -0694658469 | 010898698 | -63737746 | 184E-10 | 337853112 | 353994267 | 396198413 | 547425315 | 625303119 | 586592636 |
| Cdk1 | 126985698 | -0693862482 | 018940262 | -3663426 | 000024886 | 930819798 | 855969745 | 112911507 | 166663445 | 142721073 | 160939209 |
| Cd248 | 887853009 | -0693702668 | 021963489 | -31584357 | 000158618 | 574580122 | 783429936 | 665371381 | 114100358 | 103703801 | 112569501 |
| Clec1a | 439733973 | -0690746433 | 032138753 | -21492634 | 003161353 | 218340446 | 391714968 | 393173998 | 53845113 | 472314343 | 624408953 |
| Ifitm3 | 338311768 | -0689436719 | 012855834 | -53628316 | 819E-08 | 224086248 | 260417914 | 292360152 | 387172003 | 426109679 | 439724615 |
| Itgb2 | 876243351 | -0689342491 | 021974291 | -31370408 | 000170662 | 574580122 | 761667993 | 665371381 | 107690226 | 114998275 | 10289556 |
| Fhad1 | 182071926 | -0689059801 | 017847389 | -38608437 | 0000113 | 170075716 | 111711306 | 138114968 | 237174902 | 239237482 | 196117178 |
| Col5a2 | 179958996 | -0688936298 | 015534354 | -44349207 | 921E-06 | 131004268 | 145079618 | 13710683 | 20512424 | 23102332 | 230415698 |
| H2-Q2 | 117025371 | -0688419782 | 018820993 | -36577229 | 000025447 | 919328196 | 863223726 | 90732461 | 15384318 | 13245337 | 146868021 |
| Cd109 | 108158662 | -0683681622 | 022096384 | -30940882 | 000197419 | 106871903 | 65285828 | 786347995 | 123074544 | 151962006 | 123122892 |
| Xlr | 350459882 | -0681526395 | 033283445 | -20476438 | 004059491 | 252815254 | 290159236 | 262115998 | 448709275 | 400440421 | 448519107 |
| Cryzl2 | 170209466 | -0680940186 | 015803979 | -43086628 | 164E-05 | 128705947 | 13274785 | 131057999 | 194868028 | 222809157 | 211067815 |
| Prtg | 535753943 | -0680233229 | 010418079 | -65293536 | 661E-11 | 451619976 | 408399124 | 376035644 | 683320124 | 623249579 | 671899212 |
| Itga1 | 214950052 | -067779202 | 014587836 | -4646282 | 338E-06 | 185014799 | 154509793 | 158277738 | 251277194 | 274147673 | 266473117 |
| Gja1 | 610215748 | -0676675831 | 009578056 | -70648558 | 161E-12 | 465409899 | 438140446 | 507093643 | 752549555 | 750569097 | 747531845 |

| | | | | | | | | | | | |
|---|---|---|---|---|---|---|---|---|---|---|---|
| Skap2 | 1286788 | -0674583645 | 019527553 | -34545222 | 000055127 | 804412171 | 116789092 | 987975687 | 156407233 | 143747844 | 175889846 |
| Nid1 | 867799873 | -0674094782 | 008499892 | -79306275 | 218E-15 | 615949891 | 686226592 | 701664365 | 106408199 | 108529622 | 105358018 |
| Anxa4 | 186300455 | -0672525333 | 015937266 | -42198288 | 244E-05 | 126407627 | 138551035 | 165334707 | 241020982 | 23102332 | 215465061 |
| Cd53 | 105709467 | -0672048588 | 019747183 | -34032631 | 000066586 | 815903774 | 841461783 | 786347995 | 134612782 | 118078586 | 13719408 |
| Arpc1b | 421411021 | -067031926 | 011028663 | -60779737 | 122E-09 | 339002272 | 316998965 | 31957989 | 537169103 | 539054413 | 476661483 |
| Bst2 | 826132433 | -06691755 | 023034991 | -29050391 | 000367207 | 655021339 | 747160032 | 504069228 | 10256212 | 995967202 | 10289556 |
| Sycp2 | 449103563 | -0667654417 | 029928729 | -22308145 | 002569342 | 321764868 | 384460987 | 33268569 | 53845113 | 492849749 | 624408953 |
| Ednra | 20076064 | -066726112 | 015448005 | -43193998 | 156E-05 | 134451749 | 166116162 | 164326568 | 22691869 | 245398104 | 267352566 |
| Trim34a | 818084159 | -0666788148 | 027501949 | -24245123 | 001532896 | 356239676 | 848715764 | 675452765 | 103844146 | 872754764 | 111690052 |
| Atf3 | 403617158 | -0664456117 | 011627668 | -57144399 | 110E-08 | 286140901 | 351092675 | 296392706 | 506400467 | 491822979 | 489853221 |
| Lin7b | 13985571 | -0663798826 | 018067463 | -36740013 | 000023878 | 976786208 | 106633519 | 120976615 | 152561153 | 170443872 | 190840483 |
| Erg | 548164279 | -0662116005 | 027092612 | -24438988 | 00145295 | 436680893 | 471508758 | 362929844 | 64101325 | 708471515 | 668381415 |
| Arl6 | 892277386 | -0661925758 | 007001606 | -94539134 | 326E-21 | 636290027 | 719667444 | 717189697 | 105318477 | 104227454 | 118505784 |
| C4b | 493143424 | -0660626404 | 010499834 | -62917796 | 314E-10 | 413697688 | 358346656 | 376035644 | 612808667 | 62632989 | 571641999 |
| Colec12 | 603951231 | -0658987584 | 025617627 | -2572399 | 001009964 | 436680893 | 471508758 | 493987843 | 79485643 | 687936108 | 738737353 |
| Gpr37l1 | 624641057 | -0657673761 | 026458147 | -24857136 | 00129292 | 367731278 | 48601672 | 594801689 | 79485643 | 800880842 | 703559384 |
| Piwil4 | 112776986 | -0657072613 | 020028288 | -32807227 | 000103541 | 769937364 | 100830334 | 836754918 | 12820265 | 155042317 | 131917384 |
| Hmgb1-ps4 | 761470123 | -0655667131 | 02335377 | -2807543 | 00049921 | 60905493 | 623842357 | 544394766 | 807676695 | 954896389 | 10289556 |
| E2f6 | 146398014 | -0655213185 | 007337589 | -89295437 | 428E-19 | 110089551 | 111203527 | 119867662 | 182816979 | 170957257 | 183453109 |
| Blvrb | 171174048 | -0653864321 | 018268879 | -35791158 | 000034476 | 98827781 | 161763774 | 136098692 | 20512424 | 223835928 | 201393874 |
| Fcer1g | 116028194 | -0653835595 | 02663093 | -24551737 | 001408165 | 448172495 | 121866879 | 101821984 | 151279127 | 124239208 | 152144717 |
| Robo4 | 137443673 | -0652563747 | 017347809 | -37616494 | 00001688 | 102275262 | 103731927 | 114927784 | 162817365 | 169417101 | 1714926 |
| Cd84 | 650676162 | -0650080342 | 025769648 | -2522659 | 001164713 | 482647303 | 507778662 | 534313382 | 61537272 | 89329017 | 870654738 |
| Zfp97 | 779608588 | -0649969729 | 009895712 | -65681957 | 509E-11 | 547000276 | 617313774 | 656298135 | 912802868 | 899450792 | 104478569 |
| Hvcn1 | 390319916 | -0649919719 | 032824525 | -19799821 | 004770554 | 367731278 | 282905255 | 262115998 | 56409166 | 390172718 | 474902584 |
| Map3k8 | 7760728 | -06498298 | 02412628 | -26934522 | 000707163 | 60905493 | 594826433 | 604883073 | 114100358 | 739274624 | 967394153 |
| Irgm1 | 137344167 | -0648705349 | 017800774 | -36442536 | 000026817 | 106871903 | 110985908 | 102830122 | 174355604 | 147854925 | 181166541 |
| Tubb6 | 407831265 | -0648704812 | 010987877 | -5903823 | 355E-09 | 302229144 | 332957723 | 315547337 | 517938706 | 505170993 | 473143686 |
| Muc2 | 523820764 | -0644743181 | 01082988 | -59533733 | 263E-09 | 37462624 | 443218232 | 404263521 | 680756071 | 656106229 | 583954289 |
| S100a6 | 766576971 | -064380342 | 010817893 | -59512829 | 266E-09 | 502183027 | 663739251 | 625045843 | 916648947 | 101239553 | 87944923 |
| Higd1b | 944537972 | -0641057071 | 027114793 | -23642337 | 001806741 | 448172495 | 826953821 | 937568764 | 84613749 | 113971505 | 146868021 |
| Anxa3 | 298522411 | -0640697009 | 012652868 | -50636506 | 411E-07 | 206848844 | 244459156 | 246993922 | 369223632 | 360396379 | 363212532 |
| Abracl | 126211091 | -06400455 | 019915076 | -32138742 | 000130957 | 838886979 | 928509554 | 119968476 | 134612782 | 150935236 | 175010397 |

| | | | | | | | | | | | |
|---|---|---|---|---|---|---|---|---|---|---|---|
| Angptl4 | 101967433 | -0639776026 | 021971438 | -29118532 | 000359291 | 655021339 | 739906051 | 998057071 | 112818332 | 142721073 | 116966748 |
| Kdelr3 | 198791711 | -0636232156 | 016442798 | -38693666 | 000010912 | 121810986 | 161763774 | 18247306 | 229482743 | 253612267 | 243607437 |
| Snhg1 | 318089326 | -0635674587 | 032094399 | -19806402 | 004763163 | 150539992 | 309744984 | 286311321 | 342301075 | 392226259 | 427412326 |
| Ugt1a6a | 460557625 | -0633777096 | 03129939 | -20248864 | 004287903 | 344748073 | 297413216 | 443580921 | 64101325 | 420975827 | 615614461 |
| Arhgdib | 33990811 | -0632975923 | 013054043 | -48488881 | 124E-06 | 228682889 | 286532245 | 282278768 | 433324957 | 370664082 | 437965716 |
| Il1r1 | 224213721 | -062870352 | 015271651 | -41168014 | 384E-05 | 205699684 | 158862181 | 165334707 | 289737989 | 243344564 | 282303203 |
| Fignl1 | 724946172 | -0628659763 | 024167327 | -26012797 | 000928767 | 540105315 | 587572452 | 584720304 | 71793484 | 934360983 | 984983137 |
| Clec7a | 645161236 | -062511279 | 025825474 | -24205278 | 001549799 | 425189291 | 478762739 | 614964458 | 84613749 | 811148546 | 694764892 |
| LOC1081673 | 117435178 | -0624496544 | 019544137 | -31953139 | 000139679 | 746954159 | 105182723 | 957731533 | 147433047 | 141694303 | 139832428 |
| Tagln2 | 713621491 | -0624128441 | 00864332 | -72209344 | 516E-13 | 576878443 | 549126353 | 559516843 | 874342073 | 875835075 | 846030159 |
| S100a4 | 634245446 | -062374776 | 026266133 | -23747225 | 001756215 | 436680893 | 500524681 | 564557535 | 64101325 | 739274624 | 923421691 |
| Slc11a1 | 495576346 | -0622226343 | 028689693 | -21688149 | 003009674 | 367731278 | 442492834 | 352848459 | 66665378 | 616062187 | 527669538 |
| Tpm4 | 224899728 | -0621412778 | 006283733 | -98892303 | 464E-23 | 171339792 | 180333965 | 1798519 | 265892296 | 27425035 | 277730067 |
| Rnf128 | 111448271 | -062109256 | 020109863 | -30884973 | 000201172 | 953803003 | 935763535 | 746022457 | 134612782 | 120132126 | 150385818 |
| Gm41263 | 41545156 | -0620640788 | 011403191 | -5442694 | 525E-08 | 287290061 | 333683121 | 359905429 | 506400467 | 504144223 | 501286061 |
| Gipr | 313709824 | -0620374197 | 012098403 | -51277364 | 293E-07 | 242472812 | 259692516 | 238928814 | 38076187 | 365530231 | 394872704 |
| Nmi | 952982695 | -0618224103 | 021169151 | -2920401 | 000349581 | 689496147 | 81244586 | 746022457 | 112818332 | 130399829 | 103775009 |
| Lyn | 288857351 | -0615095896 | 014071457 | -43712311 | 124E-05 | 260859376 | 192955892 | 232879983 | 375633764 | 323432648 | 347382446 |
| Agtpbp1 | 122947473 | -0610909562 | 004861953 | -12565106 | 328E-36 | 963685781 | 965432317 | 989991963 | 145920256 | 150575866 | 149277712 |
| Mcub | 512065559 | -0610113597 | 028942258 | -21080373 | 003502776 | 310273266 | 398968949 | 504069228 | 653833515 | 554455968 | 65079243 |
| St3gal6 | 851674368 | -0610039362 | 024386538 | -25015415 | 001236539 | 482647303 | 797937898 | 735941073 | 858957755 | 104730572 | 118725646 |
| Hebp1 | 927718862 | -0609593903 | 023379377 | -26074001 | 000912327 | 60905493 | 681874204 | 90732461 | 134612782 | 913825577 | 110810603 |
| Gm3604 | 841027281 | -0609233699 | 024129324 | -25248685 | 001157416 | 574580122 | 805191879 | 614964458 | 79485643 | 107810883 | 117846197 |
| Ddx4 | 466671052 | -0608848769 | 030988627 | -19647491 | 004944328 | 298781664 | 40622293 | 393173998 | 71793484 | 544188265 | 439724615 |
| Tlr4 | 630436543 | -0607804777 | 025732096 | -23620492 | 001817423 | 413697688 | 544048567 | 534313382 | 74357537 | 852219358 | 694764892 |
| Glipr2 | 129682922 | -0607009821 | 018169978 | -33407295 | 000083559 | 113766864 | 97928742 | 977894302 | 1499971 | 148881695 | 169733701 |
| Ptprb | 339008992 | -0606660932 | 011901653 | -50972831 | 345E-07 | 278096779 | 271298885 | 257075306 | 394864162 | 440484463 | 392234357 |
| Gm52861 | 816038104 | -0604990817 | 022734325 | -26611338 | 00077878 | 632038135 | 747160032 | 554476151 | 10256212 | 934360983 | 100257212 |
| Lpar6 | 281767372 | -0604301486 | 012651607 | -477648 | 178E-06 | 210296325 | 235754379 | 223806737 | 337172969 | 332673581 | 350900243 |
| Tm6sf1 | 824396955 | -060405571 | 02513229 | -24035044 | 001623877 | 413697688 | 739906051 | 79642938 | 10256212 | 104730572 | 923421691 |
| Shcbp1 | 521587225 | -06026182 | 027375741 | -22012854 | 002771582 | 436680893 | 40622293 | 403255382 | 576911925 | 646865296 | 659586922 |
| Ets1 | 304657775 | -0601411359 | 012405606 | -48478998 | 125E-06 | 23327953 | 25824172 | 232879983 | 374351738 | 380931785 | 348261895 |
| Des | 490844223 | -0601235624 | 030420814 | -19763956 | 004810999 | 4711557 | 413476911 | 282278768 | 66665378 | 636597593 | 474902584 |

| | | | | | | | | | | | |
|---|---|---|---|---|---|---|---|---|---|---|---|
| Gm10030 | 131997029 | -0598853651 | 018238675 | -32834273 | 000102553 | 896344991 | 114612898 | 109887092 | 155125206 | 152988776 | 169733701 |
| Olfml2b | 89640045 | -0597649644 | 021499436 | -27798387 | 000543859 | 655021339 | 718144108 | 766185226 | 99998067 | 120132126 | 103775009 |
| Tnnt1 | 23289356 | -0597030319 | 014965025 | -39895044 | 662E-05 | 155136633 | 192955892 | 207676522 | 2564053 | 284415376 | 300771637 |
| Gm19378 | 91663359 | -0595698421 | 020928538 | -28463452 | 000442242 | 678004544 | 761667993 | 746022457 | 108972252 | 116025045 | 106413357 |
| Uhrf1 | 443750651 | -0592508863 | 029788334 | -19890635 | 00466942 | 310273266 | 348191083 | 403255382 | 5128106 | 533920562 | 554053015 |
| Dnlz | 382247491 | -0591889117 | 011950468 | -4952853 | 731E-07 | 287290061 | 290884634 | 335710106 | 491016149 | 468207262 | 420376732 |
| Gm6548 | 632310167 | -059144722 | 026287966 | -22498782 | 002445668 | 379222881 | 558556529 | 564557535 | 858957755 | 729006921 | 703559384 |
| Mgst1 | 247037347 | -0590227077 | 01391223 | -42425051 | 221E-05 | 173523197 | 195857484 | 22179046 | 294866095 | 293656309 | 302530535 |
| Cd44 | 74568158 | -0589832184 | 009889784 | -5964055 | 246E-09 | 679153705 | 542597771 | 567581951 | 919213 | 873781535 | 891761519 |
| Zfp966 | 191404792 | -0589508411 | 021860454 | -26966888 | 000700327 | 148241672 | 163939968 | 146180076 | 17563763 | 336780662 | 177648744 |
| Col6a3 | 966143312 | -0585663008 | 020793305 | -28165942 | 000485358 | 884853388 | 754414013 | 68553415 | 110254279 | 119105356 | 117846197 |
| Mis18bp1 | 459870764 | -0585159651 | 029188563 | -20047566 | 004498907 | 379222881 | 333683121 | 393173998 | 576911925 | 513385155 | 562847507 |
| Grm4 | 240212221 | 0584991227 | 014082638 | 415398884 | 327E-05 | 311422426 | 27347508 | 279254352 | 210252346 | 190979278 | 175889846 |
| Ttc34 | 671763867 | 0585938231 | 025161723 | 232868883 | 001987556 | 930819798 | 689128185 | 806510765 | 499990335 | 585259077 | 518875046 |
| Dnah11 | 147510419 | 0589374923 | 017313663 | 340410303 | 000066382 | 199953883 | 163939968 | 168359122 | 119228464 | 120132126 | 113448951 |
| Mal | 671177257 | 059105699 | 026043058 | 226953755 | 002323566 | 873361786 | 906747611 | 635127227 | 53845113 | 616062187 | 4573136 |
| Adat3 | 631120993 | 0595760546 | 025186973 | 236535192 | 001801295 | 792920569 | 768921974 | 715778304 | 53845113 | 451778937 | 518875046 |
| Gal3st3 | 383218372 | 0599911745 | 011341489 | 52895327 | 123E-07 | 475752341 | 452648408 | 457694859 | 289737989 | 33062004 | 292856594 |
| Gna14 | 736066591 | 0601046154 | 024709569 | 243244294 | 001499735 | 105722743 | 855969745 | 746022457 | 679474045 | 523652859 | 554053015 |
| Wnk4 | 211624364 | 0602358526 | 014829492 | 406189585 | 487E-05 | 279245939 | 259692516 | 226831153 | 169227498 | 163256479 | 1714926 |
| Igfn1 | 667679226 | 0607745608 | 009292886 | 653990188 | 616E-11 | 803263011 | 773274363 | 84179561 | 53845113 | 552402427 | 496888815 |
| Pou3f3 | 879279269 | 0608913773 | 024890856 | 244633519 | 001443168 | 136750069 | 754414013 | 107870815 | 64101325 | 739274624 | 694764892 |
| Rab3b | 275354782 | 0619410979 | 014898784 | 415745983 | 322E-05 | 267754337 | 359797452 | 37200309 | 221790584 | 201246981 | 229536249 |
| Pcdhga8 | 138624725 | 0620431304 | 018135331 | 34211193 | 000062364 | 18731312 | 160312978 | 157269599 | 108972252 | 965164092 | 121363994 |
| Wdr46 | 142319077 | 0623237048 | 018863406 | 330394759 | 000095334 | 206848844 | 14798121 | 16331843 | 120510491 | 114998275 | 100257212 |
| A930017K11F | 86148598 | 0635494782 | 021828723 | 29112779 | 000359954 | 111468544 | 106633519 | 967812917 | 61537272 | 718739218 | 685970399 |
| H19 | 432216029 | 0643575047 | 014035123 | 458546056 | 453E-06 | 399907765 | 596277229 | 582704027 | 343583102 | 335753892 | 335070157 |
| Slc13a5 | 113687948 | 0644768589 | 020910732 | 30834338 | 000204627 | 164329915 | 1138875 | 138114968 | 101280093 | 800880842 | 844271261 |
| Glb1l3 | 29270965 | 064981978 | 013020528 | 499073312 | 602E-07 | 319466548 | 384460987 | 367970536 | 223072611 | 223835928 | 237451292 |
| Asb2 | 563115144 | 0651789709 | 028248128 | 230737312 | 002103403 | 838886979 | 565810509 | 665371381 | 474349805 | 359369609 | 474902584 |
| Prickle4 | 544472806 | 0654797987 | 030605112 | 213950528 | 003239477 | 689496147 | 819699841 | 493987843 | 30768636 | 410708124 | 545258523 |
| Gm40078 | 385453316 | 0658125184 | 032499633 | 202502343 | 004286496 | 4711557 | 457000796 | 493987843 | 23076477 | 308031093 | 351779692 |
| Cilp2 | 432155608 | 0658540958 | 03041743 | 216501182 | 003038678 | 482647303 | 500524681 | 604883073 | 33332689 | 328566499 | 3429852 |

| | | | | | | | | | | | |
|---|---|---|---|---|---|---|---|---|---|---|---|
| Rps2-ps10 | 164563216 | 0662249698 | 017413033 | 380318403 | 000014285 | 23557785 | 192955892 | 17642423 | 134612782 | 13348014 | 1143284 |
| 9630028B13F | 707860168 | 0670563218 | 024093281 | 278319589 | 000538263 | 919328196 | 870477707 | 816592149 | 551271395 | 605794483 | 483697076 |
| Krt1 | 460774656 | 0676283449 | 030029413 | 225207016 | 002431784 | 586071725 | 500524681 | 614964458 | 397428215 | 287495687 | 378163169 |
| Adat2 | 513413952 | 0685084941 | 028989675 | 236320327 | 001811773 | 666512942 | 500524681 | 735941073 | 397428215 | 410708124 | 369368677 |
| 3010001F23F | 402225784 | 0692335573 | 033080959 | 209285218 | 003636235 | 379222881 | 478762739 | 635127227 | 269225565 | 308031093 | 3429852 |
| Perp | 814547134 | 0704213561 | 024248808 | 290411621 | 000368291 | 781428966 | 986541401 | 126017307 | 602552455 | 605794483 | 65079243 |
| Uty | 709792782 | 072517241 | 009180043 | 78994443 | 280E-15 | 917029875 | 877731688 | 858933964 | 556399501 | 492849749 | 555811913 |
| Kdm5d | 628155664 | 0735145129 | 009241485 | 795483748 | 179E-15 | 788323928 | 789233121 | 775258472 | 499990335 | 451778937 | 464349193 |
| Gm13584 | 446063357 | 0741906036 | 033571098 | 220995464 | 002710831 | 60905493 | 398968949 | 675452765 | 243585035 | 441511234 | 30780723 |
| Nptxr | 378027563 | 0754149383 | 014483972 | 520678565 | 192E-07 | 568834321 | 375030812 | 481890182 | 293584068 | 270040592 | 278785406 |
| Ppp1ccb | 128028161 | 0757570168 | 022732959 | 333247491 | 000086077 | 222937087 | 13274785 | 128033584 | 99998067 | 841951655 | 100257212 |
| Gm14137 | 500229569 | 0761944992 | 029756227 | 25606237 | 001044845 | 735462557 | 587572452 | 564557535 | 371787685 | 451778937 | 290218246 |
| Ntn3 | 436962072 | 0768308191 | 035943586 | 213753908 | 003255417 | 861870183 | 391714968 | 403255382 | 346147155 | 328566499 | 290218246 |
| Gm6999 | 707587245 | 0771358324 | 024860905 | 310269605 | 000191766 | 815903774 | 950271497 | 917405995 | 43588901 | 492849749 | 633203446 |
| Dock8 | 235147348 | 0782374526 | 014510867 | 539164551 | 698E-08 | 291886702 | 309744984 | 288327598 | 20127816 | 156069087 | 163577557 |
| Asprv1 | 730511627 | 0782882731 | 025017374 | 312935617 | 00017519 | 114916024 | 819699841 | 806510765 | 576911925 | 503117452 | 527669538 |
| Ung | 256829786 | 0792059761 | 039414917 | 200954316 | 004447956 | 287290061 | 362699044 | 322604306 | 217944505 | 174550953 | 175889846 |
| Col1a2 | 221450772 | 0799712588 | 015669076 | 510376364 | 333E-07 | 313720747 | 263319506 | 266148552 | 174355604 | 176604493 | 134555732 |
| Vwa5b1 | 617730933 | 0807969136 | 026156342 | 308899897 | 000200832 | 769937364 | 696382165 | 897243226 | 41024848 | 492849749 | 439724615 |
| Ndufa12-ps | 875028026 | 0809769316 | 022797352 | 355203233 | 000038227 | 999769413 | 125493869 | 107870815 | 64101325 | 739274624 | 53646403 |
| Ywhaq-ps3 | 28680068 | 0813845589 | 038068952 | 213781977 | 003253137 | 275798459 | 384460987 | 433499536 | 217944505 | 215621765 | 193478831 |
| Pou6f2 | 269332198 | 0817199461 | 03964707 | 206118502 | 003928539 | 413697688 | 282905255 | 33268569 | 243585035 | 184818656 | 158300861 |
| Ccdc63 | 398843261 | 0818701649 | 032959394 | 248397059 | 001299265 | 643529737 | 457000796 | 433499536 | 2564053 | 277227984 | 325396215 |
| Gm46911 | 345775368 | 082235126 | 038269676 | 214883259 | 003164767 | 620546532 | 398968949 | 302441537 | 33332689 | 225889468 | 193478831 |
| Gm4202 | 367314052 | 0826808901 | 036072518 | 229207428 | 002190136 | 632038135 | 442492834 | 33268569 | 33332689 | 225889468 | 237451292 |
| Igsf1 | 510080139 | 0839876532 | 02918563 | 287770571 | 000400579 | 689496147 | 710890127 | 554476151 | 448709275 | 349101906 | 30780723 |
| Itpka | 25865936 | 0841026848 | 041840042 | 201010038 | 004442057 | 402206086 | 275651274 | 322604306 | 192303975 | 112944734 | 246245784 |
| Gm39876 | 264753329 | 0857946531 | 041884597 | 204835809 | 004052492 | 321764868 | 420730892 | 272197383 | 28204583 | 13348014 | 158300861 |
| Gm34640 | 88356892 | 0859913893 | 023626107 | 363967663 | 000027298 | 110319383 | 107358917 | 12400103 | 730755105 | 441511234 | 712353876 |
| Gm30273 | 425632907 | 08638788 | 032993965 | 261829336 | 000883708 | 574580122 | 464254777 | 614964458 | 30768636 | 205354062 | 386957661 |
| Rbx1-ps | 26211546 | 0879445272 | 040776462 | 215674736 | 003102535 | 344748073 | 333683121 | 342767075 | 192303975 | 112944734 | 246245784 |
| Wdr72 | 271927627 | 0886356187 | 039623661 | 223693666 | 002529048 | 321764868 | 333683121 | 403255382 | 166663445 | 256692578 | 149506369 |
| Lcn2 | 770109971 | 0896812753 | 014081441 | 636875674 | 191E-10 | 717075993 | 114395279 | 114322901 | 544861262 | 511331615 | 560209159 |

| | | | | | | | | | | | |
|---|---|---|---|---|---|---|---|---|---|---|---|
| Impdh2-ps | 25354193 | 0901137936 | 041694652 | 216127945 | 003067376 | 413697688 | 355445064 | 22179046 | 166663445 | 205354062 | 158300861 |
| Tmem150b | 473975606 | 0912081082 | 029728261 | 306806062 | 000215453 | 51712211 | 689128185 | 645208612 | 35896742 | 308031093 | 325396215 |
| Prokr1 | 211307374 | 0926612766 | 044736874 | 207125057 | 003833539 | 310273266 | 311921178 | 211709076 | 10256212 | 16428325 | 167095354 |
| Fam107a | 139724956 | 0933844281 | 017784906 | 52507687 | 151E-07 | 172374037 | 179898726 | 198603276 | 87177802 | 102677031 | 976188645 |
| 4930526A20I | 231824858 | 0954903496 | 043223096 | 220924362 | 00271577 | 287290061 | 348191083 | 282278768 | 115382385 | 225889468 | 131917384 |
| Gm13112 | 270825396 | 0966114151 | 01371811 | 704261833 | 189E-12 | 337853112 | 372854618 | 361921706 | 196150054 | 196113129 | 16005976 |
| Chil1 | 447117208 | 0971893137 | 0112926 | 860646055 | 754E-18 | 534359514 | 597728025 | 643192335 | 316660545 | 292629539 | 298133289 |
| 1700001K19I | 389338288 | 0979108269 | 036455606 | 268575504 | 000723661 | 769937364 | 420730892 | 362929844 | 269225565 | 266960281 | 246245784 |
| Gm30266 | 446222376 | 0979832033 | 031615886 | 309917624 | 00019406 | 746954159 | 478762739 | 554476151 | 30768636 | 308031093 | 281423754 |
| Gm12405 | 233134499 | 1002301994 | 042615599 | 235196038 | 001867476 | 367731278 | 304667197 | 262115998 | 166663445 | 112944734 | 184684338 |
| Gm21596 | 506518568 | 1008475332 | 039520568 | 255177336 | 001071762 | 976786208 | 565810509 | 504069228 | 15384318 | 266960281 | 571641999 |
| Il17rb | 16853532 | 1010126808 | 05113492 | 197541484 | 004822109 | 275798459 | 261143312 | 141139384 | 89741855 | 102677031 | 140711877 |
| Olfm4 | 290414852 | 1024490425 | 013835191 | 740496064 | 131E-13 | 333256471 | 417103901 | 417369321 | 180765736 | 202273751 | 191719932 |
| LOC1154904 | 179695811 | 1039432074 | 051719478 | 200974972 | 004445768 | 379222881 | 15958758 | 191546307 | 89741855 | 143747844 | 1143284 |
| Gm14760 | 616514545 | 1057760201 | 033147674 | 319105405 | 000141755 | 131004268 | 638350318 | 554476151 | 41024848 | 451778937 | 334190707 |
| Mid1-ps1 | 841472043 | 1079991647 | 026114112 | 413566292 | 354E-05 | 953803003 | 137100239 | 109887092 | 43588901 | 749542327 | 439724615 |
| Scube2 | 200001337 | 1081899 | 047157872 | 229420656 | 002177864 | 310273266 | 210365446 | 292360152 | 15384318 | 154015547 | 791504307 |
| Gm31796 | 180596014 | 1082280279 | 049591912 | 218237255 | 002908205 | 321764868 | 24663535 | 171383537 | 7692159 | 143747844 | 123122892 |
| Mybphl | 276312513 | 1085046498 | 039648262 | 273668109 | 000620624 | 321764868 | 384460987 | 413336767 | 243585035 | 154015547 | 140711877 |
| Pax7 | 22496816 | 109272218 | 045760467 | 238791745 | 001694415 | 402206086 | 333683121 | 181464922 | 15384318 | 16428325 | 1143284 |
| Gm40099 | 132213408 | 1112486285 | 055153444 | 201707491 | 00436877 | 206848844 | 174095541 | 161302153 | 89741855 | 821416249 | 791504307 |
| Prr15l | 235134616 | 1122567468 | 048878471 | 229665014 | 002163874 | 528613713 | 224873408 | 211709076 | 20512424 | 143747844 | 967394153 |
| F2rl1 | 759525242 | 1124397021 | 024423708 | 460371134 | 415E-06 | 110319383 | 102281131 | 100813846 | 423068745 | 410708124 | 589230984 |
| Gm17250 | 136691156 | 115244354 | 055887288 | 206208528 | 003919962 | 126407627 | 217619427 | 22179046 | 7692159 | 718739218 | 105533908 |
| Bdkrb2 | 21664663 | 115324628 | 043744198 | 263634115 | 000838054 | 241323651 | 311921178 | 342767075 | 12820265 | 143747844 | 131917384 |
| Gm2000 | 32221932 | 1170605647 | 048743096 | 240158245 | 001632433 | 838886979 | 203111465 | 302441537 | 217944505 | 13348014 | 237451292 |
| Cldn10 | 555271711 | 1189882002 | 028724757 | 41423571 | 344E-05 | 861870183 | 834207802 | 614964458 | 397428215 | 29776339 | 325396215 |
| Steap4 | 956563981 | 123451869 | 023557495 | 524044969 | 160E-07 | 124109306 | 127670064 | 152228907 | 499990335 | 451778937 | 747531845 |
| Gm10039 | 161831969 | 1257771802 | 05701262 | 220612877 | 002737499 | 298781664 | 145079618 | 241953229 | 7692159 | 16428325 | 439724615 |
| Myh7 | 136054877 | 1283075427 | 057008185 | 225068631 | 002440541 | 229832049 | 217619427 | 131057999 | 89741855 | 513385155 | 967394153 |
| Aqp5 | 190281865 | 1287404126 | 049436395 | 260416263 | 00092099 | 241323651 | 232127388 | 342767075 | 5128106 | 13348014 | 140711877 |
| Cited4 | 275412163 | 131506228 | 044010264 | 298808085 | 000280735 | 586071725 | 282905255 | 312522921 | 141022915 | 215621765 | 1143284 |
| Gm30483 | 157453333 | 131803759 | 05364692 | 245687466 | 001401516 | 321764868 | 203111465 | 151220768 | 89741855 | 821416249 | 967394153 |

| | | | | | | | | | | | |
|---|---|---|---|---|---|---|---|---|---|---|---|
| Krt6b | 175723821 | 1338946787 | 050907203 | 263017158 | 000853418 | 252815254 | 217619427 | 282278768 | 115382385 | 13348014 | 527669538 |
| Meltf | 132613642 | 1373037014 | 057284861 | 239685841 | 001653631 | 160882434 | 15958758 | 252034614 | 89741855 | 718739218 | 615614461 |
| Prss27 | 11957424 | 138818732 | 060874115 | 2280423 | 002258261 | 195357242 | 123317675 | 201627691 | 7692159 | 410708124 | 791504307 |
| Gm35365 | 126912034 | 1389433985 | 064199728 | 216423657 | 00304462 | 264306856 | 870477707 | 201627691 | 89741855 | 308031093 | 87944923 |
| A530076I17R | 113159413 | 1404858278 | 062309421 | 225464826 | 002415542 | 195357242 | 108809713 | 191546307 | 5128106 | 616062187 | 703559384 |
| Traf1 | 136941926 | 1423729397 | 056176215 | 253439892 | 001126404 | 172374037 | 210365446 | 211709076 | 10256212 | 718739218 | 527669538 |
| Gm46738 | 109350648 | 1457653885 | 064216552 | 226990368 | 002321343 | 229832049 | 123317675 | 131057999 | 38460795 | 718739218 | 615614461 |
| Gm52523 | 927543675 | 1493069188 | 069149329 | 215919548 | 0030835 | 172374037 | 137825637 | 100813846 | 38460795 | 718739218 | 351779692 |
| Gm39323 | 115256177 | 1519272836 | 064287555 | 236324565 | 001811566 | 149390832 | 101555732 | 262115998 | 64101325 | 616062187 | 527669538 |
| 5730409K12F | 833567541 | 1529726132 | 078040505 | 196016944 | 004997599 | 218340446 | 725398089 | 806510765 | 64101325 | 205354062 | 439724615 |
| Amd-ps4 | 168943657 | 1534951856 | 072253544 | 212439665 | 003363699 | 597563327 | 108809713 | 504069228 | 89741855 | 616062187 | 105533908 |
| LOC10524691 | 765150071 | 15542368 | 076703711 | 202628633 | 004273545 | 149390832 | 725398089 | 120976615 | 38460795 | 513385155 | 263834769 |
| Gm52619 | 782190956 | 1598531154 | 07869848 | 203120969 | 004223373 | 114916024 | 174095541 | 604883073 | 64101325 | 205354062 | 351779692 |
| Abcc2 | 753157873 | 1695148645 | 078075786 | 217115797 | 002991923 | 149390832 | 943017516 | 100813846 | 38460795 | 513385155 | 175889846 |
| Krt6a | 141617026 | 1747227564 | 0562032 | 310876885 | 000187869 | 206848844 | 24663535 | 201627691 | 64101325 | 513385155 | 791504307 |
| Xlr4b | 102194342 | 178283678 | 069800537 | 255418777 | 001064358 | 183865639 | 797937898 | 211709076 | 5128106 | 513385155 | 351779692 |
| BC055402 | 78463433 | 1783694252 | 025761977 | 692374752 | 440E-12 | 999769413 | 130571656 | 133074276 | 41024848 | 379905015 | 281423754 |
| Gm30938 | 857417183 | 1835231985 | 072374118 | 253575729 | 001122045 | 149390832 | 123317675 | 131057999 | 2564053 | 410708124 | 439724615 |
| Gm9824 | 141832683 | 1835899219 | 075192189 | 244160895 | 001462198 | 459664098 | 101555732 | 11089523 | 0 | 821416249 | 967394153 |
| Krt15 | 149112185 | 1836167777 | 056421725 | 325436307 | 000113647 | 172374037 | 224873408 | 302441537 | 5128106 | 821416249 | 615614461 |
| Atp13a4 | 888659822 | 1900317196 | 071650392 | 265220768 | 000799673 | 160882434 | 130571656 | 131057999 | 2564053 | 410708124 | 439724615 |
| Vis1 | 55776736 | 1995493771 | 099264497 | 201027944 | 004440162 | 459664098 | 116063694 | 100813846 | 5128106 | 205354062 | 0 |
| Krt12 | 433770265 | 2065526524 | 033808673 | 610945761 | 100E-09 | 60905493 | 768921974 | 725859688 | 141022915 | 16428325 | 193478831 |
| Actb-ps1 | 611932538 | 217833609 | 100445587 | 216867277 | 003010754 | 137899229 | 870477707 | 806510765 | 0 | 0 | 615614461 |
| Gm17803 | 581816985 | 2187576092 | 098146775 | 22288823 | 002582174 | 183865639 | 435238853 | 604883073 | 12820265 | 308031093 | 175889846 |
| Gm39786 | 394789856 | 2219363526 | 110612709 | 200642724 | 004481068 | 689496147 | 65285828 | 604883073 | 12820265 | 205354062 | 087944923 |
| Ldoc1 | 814590562 | 2246399049 | 081319349 | 276244102 | 000573709 | 218340446 | 943017516 | 90732461 | 38460795 | 205354062 | 263834769 |
| Gm31399 | 416304503 | 2307043295 | 109277639 | 2111176 | 003475719 | 689496147 | 580318471 | 806510765 | 12820265 | 205354062 | 087944923 |
| Trp63 | 166463169 | 2327682552 | 057133984 | 407407709 | 462E-05 | 356239676 | 275651274 | 201627691 | 64101325 | 308031093 | 703559384 |
| Anxa8 | 432780271 | 2358793885 | 109737199 | 214949344 | 003159531 | 103424422 | 435238853 | 705696919 | 12820265 | 205354062 | 087944923 |
| Gm10433 | 356057382 | 2573919699 | 125653658 | 204842401 | 004051847 | 804412171 | 725398089 | 302441537 | 12820265 | 0 | 175889846 |
| Mb | 173816268 | 2593653187 | 056920406 | 455663156 | 520E-06 | 287290061 | 261143312 | 342767075 | 7692159 | 308031093 | 439724615 |
| Gm14824 | 368383869 | 263532882 | 125609087 | 209803994 | 003590162 | 103424422 | 580318471 | 302441537 | 0 | 205354062 | 087944923 |

| | | | | | | | | | | | |
|---|---|---|---|---|---|---|---|---|---|---|---|
| Krt5 | 189996177 | 2654936836 | 054427223 | 487795758 | 107E-06 | 356239676 | 304667197 | 322604306 | 64101325 | 308031093 | 615614461 |
| Gm10269 | 266884652 | 2917415093 | 141049646 | 206836045 | 003860614 | 128705947 | 870477707 | 403255382 | 7692159 | 308031093 | 791504307 |
| Gm32768 | 279279312 | 2920367947 | 148094932 | 197195671 | 004861455 | 459664098 | 435238853 | 604883073 | 0 | 0 | 175889846 |
| Gm50287 | 298840284 | 2990780431 | 143176228 | 208888059 | 003671847 | 574580122 | 725398089 | 302441537 | 0 | 102677031 | 087944923 |
| Rps18-ps3 | 314581212 | 3089103811 | 153974669 | 200624156 | 004483048 | 126407627 | 145079618 | 302441537 | 0 | 0 | 175889846 |
| Gm17875 | 367560087 | 32409151 | 148627753 | 21805585 | 002921609 | 919328196 | 072539809 | 100813846 | 0 | 205354062 | 0 |
| Gm39425 | 566337374 | 3321105669 | 113931915 | 291499152 | 000355698 | 804412171 | 137825637 | 90732461 | 0 | 308031093 | 0 |
| Gm3985 | 383598275 | 3354750572 | 151795056 | 221005259 | 002710151 | 0 | 108809713 | 100813846 | 0 | 205354062 | 0 |
| LOC1008619 | 425694404 | 3492122523 | 150618371 | 231852363 | 002042088 | 114916024 | 217619427 | 201627691 | 0 | 205354062 | 0 |
| Gm9522 | 318639544 | 3512838058 | 048859525 | 71896689 | 649E-13 | 60905493 | 638350318 | 514150612 | 2564053 | 718739218 | 527669538 |
| Gm36736 | 242792251 | 3585133353 | 161975924 | 221337422 | 002687186 | 574580122 | 290159236 | 504069228 | 0 | 0 | 087944923 |
| Cldn4 | 245865842 | 3618468464 | 175696315 | 205950162 | 003944621 | 574580122 | 797937898 | 0 | 0 | 102677031 | 0 |
| Alb | 16196624 | 38348482 | 171248612 | 223934556 | 002513344 | 873361786 | 145079618 | 201627691 | 2564053 | 205354062 | 175889846 |
| Dhrs9 | 137387063 | 3859123057 | 195990883 | 196903193 | 004894942 | 114916024 | 507778662 | 201627691 | 0 | 0 | 0 |
| Gm16073 | 146485203 | 3908530397 | 189216065 | 206564406 | 00388621 | 459664098 | 217619427 | 201627691 | 0 | 0 | 0 |
| Odf3l1 | 158260245 | 4004899829 | 19053699 | 210190148 | 003556191 | 574580122 | 072539809 | 302441537 | 0 | 0 | 0 |
| Gm15191 | 156236421 | 4015697865 | 189568056 | 211834101 | 00341462 | 114916024 | 217619427 | 604883073 | 0 | 0 | 0 |
| Gm7511 | 162960971 | 4042680966 | 195547107 | 206736936 | 003869936 | 804412171 | 072539809 | 100813846 | 0 | 0 | 0 |
| Gm10536 | 172700576 | 4133290868 | 184590223 | 223917107 | 002514479 | 689496147 | 145079618 | 201627691 | 0 | 0 | 0 |
| Gm17980 | 189502884 | 4268116607 | 179025964 | 238407688 | 001712203 | 689496147 | 145079618 | 302441537 | 0 | 0 | 0 |
| Mgat4e | 18949127 | 4272484648 | 195492723 | 218549549 | 002885254 | 919328196 | 217619427 | 0 | 0 | 0 | 0 |
| Krt17 | 19420361 | 4300183006 | 184655056 | 232876538 | 00198715 | 919328196 | 145079618 | 100813846 | 0 | 0 | 0 |
| Gsx1 | 192494712 | 4327276068 | 174005659 | 248685938 | 001288763 | 344748073 | 507778662 | 302441537 | 0 | 0 | 0 |
| Acox2 | 213694433 | 445336453 | 17081964 | 26070565 | 000913243 | 459664098 | 217619427 | 604883073 | 0 | 0 | 0 |
| 1700047F07 | 264078129 | 4755769226 | 163403511 | 291044495 | 000360915 | 919328196 | 362699044 | 302441537 | 0 | 0 | 0 |
