# Supplementary material for: Zeb2 Controls Retinal Physiological and Pathological Angiogenesis by Regulating Astrocyte Proliferation and Differentiation
Source: Cell Prolif. 2026 May 26;59(10):e70236. doi: 10.1111/cpr.70236 (PMC13325830; doi:10.1111/cpr.70236)
Supplement: Supplementary file 3 [file CPR-59-e70236-s001.docx]

**Supplementary Figures S1-S11**

**Figure S1. Zeb2 is transiently expressed in astrocyte progenitor cells in developing mouse retinas.** **(A-D)** Double-immunofluorescence staining of wild-type whole-mount retinas for Pax2 (green) and Zeb2 (red) with DAPI counterlabeling (blue) at developmental stages E15.5, E17.5 and P0. The image shown in (B) is from the central region of a representative E17.5 retina and that in (C) is from the intermediate to peripheral region. Note that the Zeb2-immunoreactive cells in (D) are retinal ganglion cells and amacrine cells located within the retinal ganglion cell layer. Abbreviation: ONH, optic nerve head. The insets in (B,C) show corresponding outlined regions at a higher magnification. Scale bar, 100 μm.

**Figure S2. The retinal and eyeball sizes of control and Zeb2CKO mice.** **(A)** PCR analysis of genomic DNA from Zeb2^fl/fl^, Zeb2^+/+^ and Zeb2^+/fl^ mice. The wild-type and floxed (fl) alleles yield a product of 197 bp and 339 bp, respectively. **(B)** Retinal morphology of P16 control and Zeb2CKO mice. **(C)** The morphology of eyeballs (upper) and retinas (lower) of P25 control and Zeb2CKO animals. Scale bar: B,C, 1 mm.

**Figure S3. The astrocytes and vasculature are normal in Zeb2CKO retinas after P12.** **(A)** P12 control and Zeb2CKO flat-mount retinas were triple-immunostained for IB4, Pax2 and GFAP. Shown are representative confocal images in the central, intermediate and peripheral regions. **(B-D)** Quantification of Pax2^+^ cells (B), GFAP^+^ area (C) and GFAP^+^/Pax2+ area ratio (D) in P12 control and Zeb2CKO retinas in the central, intermediate and peripheral regions. Data are presented as mean ± SEM (n = 6). **(E)** IB4 immunofluorescence (vitreal surface vasculature, blue; IPL vasculature, green; OPL vasculature, red) images of P16 control and Zeb2CKO flat-mount retinas in the central, intermediate and peripheral regions. **(F-H)** Quantification of IB4^+^ vascular area of P16 control and Zeb2CKO retinas in the central (F), intermediate (G) and peripheral (H) regions. Data are presented as mean ± SEM (n = 7). Abbreviations: IPL, inner plexiform layer; OPL, outer plexiform layer. Scale bar: E, 200 μm; A, 100 μm.

**Figure S4. Increased endothelial cells in P0 Zeb2CKO retinas.** **(A)** ERG immunofluorescence images of P0 control and Zeb2CKO flat-mount retinas around the ONH (optic nerve head) area. **(B)** Quantification of all ERG^+^ cells in the region beyond 150 μm from the ONH (denoted in A) in P0 control and Zeb2CKO retinas. Data are presented as mean ± SEM (n = 5-6). **p < 0.01. Scale bar: A, 100 μm.

**Figure S5. Altered transcriptome profiles detected by RNA-seq analysis between E17.5 Zeb2CKO and control retinas.** (**A**) Principal component (PC) analysis of RNA-seq data showing that the Zeb2CKO retinal samples (red) are discriminated from the control samples (yellow). (**B**) Scatter plot of global gene expression profiles in CKO and control retinas. Gene expression levels are depicted in log10 scale. The diagonal line represents equal expression in the two genotypes. (**C**) Volcano plot (significance vs fold change) of significantly downregulated (green) and upregulated (red) genes (fold change ≥1.5 and p < 0.05) between the CKO and control retinas. (**D**) Heatmap of differentially expressed genes (DEGs) reveals a large group of significantly upregulated genes as well as a smaller cluster of significantly downregulated ones in CKO retinas. (**E**) Top 16 enriched GO terms plus 4 representative GO terms associated with glial cell differentiation, FGF receptor signaling, or GDNF receptor signaling for the DEGs between the CKO and control retinas. (**F**) GSEA of the RNA-seq data identifies enriched gene sets associated with endothelium development, endothelial cell differentiation, establishment of endothelial barrier, vasculogenesis, and epithelial cell development. (**G**) Network plot of 4 representative enriched angiogenesis-related GO terms or gene sets (nodes) and their associated DEGs. Node size represents the gene-set size. (**H**) Expression heatmap of a set of DEGs involved in angiogenesis, gliogenesis or FGF signaling. (**I**) qRT-PCR assay of the RNA expression levels of the indicated genes involved in angiogenesis, gliogenesis or FGF signaling in P0 control and Zeb2CKO retinas. Data are presented as mean ± SEM (n = 3). *p < 0.05; **p < 0.01; ***p < 0.001.

**Figure S6. Upregulation of *Vegfa* expression in P0 Zeb2CKO retinas.** **(A)** Relative RNA expression levels of *Hif1a*, *Epas1 (Hif2a), Vegfa, Angpt1, Angpt2,* and *Fgf2* were determined by qRT-PCR assay in P0 control and Zeb2CKO retinas. Data are presented as mean ± SEM (n =3). **p < 0.01. **(B)** Western blot analysis of HIF2a and VEGFA protein levels in 3 each P0 control and Zeb2CKO retinas. GAPDH served as the internal protein control. **(C,D)** Quantification of HIF2a and VEGFA protein levels in P0 control and Zeb2CKO retinas. Data are presented as mean ± SEM (n =3). *p < 0.01; ns, no significance. **(E)** Representative confocal images of HIF2a immunofluorescence and DAPI labeling of P0 control and Zeb2CKO retinal sections. Abbreviations: GCL, ganglion cell layer; NBL, neuroblastic layer. Scale bar: D, 50 μm.

**Figure S7. Direct regulation of downstream gene expression by Zeb2.** **(A)** Schematic of the *Vegfa* promoter region with the positions of the 4 putative Zeb2 binding motifs [5’-CACCT(G)-3’] indicated. The horizontal arrows indicate the positions of PCR primers used to amplify the precipitated DNA fragments (sites 1-4) in the chromatin immunoprecipitation (ChIP) assay. The negative control fragments (sites 3 and 4) without binding motifs are located within the intron region. Indicated also is the transcription start site (TSS). **(B)** In the ChIP assay, chromatin DNA was prepared from adult mouse retinas, immunoprecipitated by an anti-Zeb2 antibody, and quantified by qRT-PCR. Data are presented as mean ± SEM (n = 3). **p < 0.01; ****p < 0.0001; ns, no significance. **(C)** Schematic of the luciferase assay. A 2-kb *Vegfa* promoter (P) fragment was inserted upstream of Luc (luciferase) in the pGL3-Basic vector. The open reading frame of the Zeb2 transcription factor (TF) gene was inserted into the pCI expression vector. **(D)** Relative luciferase activities after cotransfection of the *Vegfa* reporter plasmid with the control (pCI) plasmid or the indicated increasing amount of Zeb2 expression plasmid in 293T cells. Histograms represent the mean ± SEM of triplicate assays in a single experiment. ***p < 0.001; ****p < 0.0001. **(E)** CUT&Tag analysis of E17.5 mouse retinas were conducted to map genomic sites bound by Zeb2. Shown are heatmaps of the Zeb2 and H3K27me3 CUT&Tag signals around the Zeb2 CUT&Tag peak region. Each row represents a 1-kb region centered on the Zeb2 peak summit, sorted by Zeb2 or H3K27me3 signal enrichment. IgG serves as the negative control. **(F)** A top-ranked Zeb2-binding motif (P = 1e-67) identified by de novo motif search in a 300-bp window centered at the peak summit. **(G)** Genome browser views of Zeb2, H3K27me3, and IgG CUT&Tag signals at the *Vegfa, Cdkn1b, Cyp1b1,* and *Nkx6-1* loci. The y axis represents the number of normalized reads.

**Figure S8. Upregulation of Zeb2 expression in retinal astrocytes after hypoxic injury.** **(A)** Pax2 and Zeb2 double-immunofluorescence staining of flat-mount retinas from wild-type mice at P12 under the normoxia condition, or 8 (P12-8h) and 24 (P13-24h) hours post vaso-obliteration under the OIR condition. The insets show the corresponding outlined regions at a higher magnification. **(B)** Pax2, Zeb2 and ERG triple-immunofluorescence staining of flat-mount retinas from wild-type mice at 8 (P12-8h) and 24 (P13-24h) hours post vaso-obliteration under the OIR condition. Abbreviations: AVA, avascular area; OIR, oxygen-induced retinopathy; ONH, optic nerve head; VA, vascular area. Scale bar: A, B, 100 μm.

**Figure S9. GO enrichment, KEGG pathway enrichment and gene set enrichment analyses (GSEA)** **of the downregulated DEGs between P17 OIR Zeb2CKO and control retinas.** (**A,B**) Top 20 enriched GO terms (A) and KEGG pathways (B) for the downregulated DEGs. (**C**) GSEA of the downregulated genes identifies enriched gene sets associated with inflammatory response, regulation of inflammatory response, positive regulation of inflammatory response, cell chemotaxis, and response to cytokine. (**D**) Network plot of the top 5 enriched GO terms and their associated downregulated DEGs.

**Figure S10. Unaltered expression of HIF/VEGF pathway components in P17 Zeb2CKO retinas.** **(A)** Relative RNA expression levels of *Hif1a, Epas1(Hif2a), Vegfa,* *Slc2a1(Glut1),* and *Ldha* were determined by qRT-PCR assay in P17 OIR control and Zeb2CKO retinas. Data are presented as mean ± SEM (n =3). ns, no significance. **(B)** Western blot analysis of HIF2a and VEGFA protein levels in 3 each P17 OIR control and Zeb2CKO retinas. GAPDH served as the internal protein control. **(C,D)** Quantification of HIF2a (C) and VEGFA (D) protein levels in P17 OIR control and Zeb2CKO retinas. Data are presented as mean ± SEM (n =3). ns, no significance. **(E)** Representative confocal images of HIF2a immunofluorescence and DAPI labeling of retinal sections from P17 control and Zeb2CKO animals under the normoxic and OIR conditions. Abbreviations: GCL, ganglion cell layer; INL, inner nuclear layer; OIR, oxygen-induced retinopathy; ONL, outer nuclear layer. Scale bar: E, 50 μm.

**Figure S11. Assessment of the purity of the primary astrocytes (pACs) by immunofluorescence.** **(A-C)** Representative confocal images of pACs immunostained for astrocyte-specific protein markers GFAP (A), S100b (B) or Sox9 (C) and counterstained with DAPI. pACs were isolated from P2 pups of control mice. Scale bar: 20 μm. **(D)** Quantification of the proportions of marker-positive pACs (GFAP^+^/DAPI^+^, S100b^+^/DAPI^+^ or Sox9^+^/DAPI^+^). Data are presented as mean ± SEM (n = 20).
