# Supplementary material for: Zeb2 Controls Retinal Physiological and Pathological Angiogenesis by Regulating Astrocyte Proliferation and Differentiation
Source: Cell Prolif. 2026 May 26:e70236. Online ahead of print. doi: 10.1111/cpr.70236 (PMC13325830; doi:10.1111/cpr.70236)
Supplement: Supplementary file 1 — Table S1: List of genes differentially expressed in E17.5 Zeb2CKO retinas as determined by RNA‐seq analysis [file CPR-9999-e70236-s001.pdf]

Table S1. List of genes differentially expressed in E17.5 *Zeb2* CKO retinas as determined by RNA-seq analysis

gene	baseMean	log2FoldChang	lfcSE	stat	pvalue	CKO1	CKO2	CKO3	Control1	Control2	Control3
Iqcm	409698288	-5519231471	147368909	-37451804	000018026	0	0	0	111110479	592709112	754375826
Gm52437	140575422	-5401422126	112046797	-48206841	143E-06	0	097185374	096922715	290596638	256840615	276604469
Mb	72893648	-5350822622	209284414	-25567229	001056633	098611842	0	0	170939199	385260923	251458609
Myh4	271652615	-5336330719	230922059	-23108796	002083951	394447367	0	0	170939199	142250187	150875165
Tnni2	973006364	-4245905404	206743225	-20537096	004000381	098611842	0	193845431	085469599	533438201	125729304
Fhad1os2	15643838	-4084332264	194821258	-2096451	00360422	0	0	0	085469599	098784852	754375826
Gm51820	141571976	-3999106985	192373427	-2078825	003763343	0	0	0	427347997	296354556	125729304
Cbfa2t2-ps1	136308527	-3919121691	195298694	-20067322	004477819	0	0	0	341878398	098784852	377187913
Gm46843	224537646	-3537266593	170607535	-20733355	003814107	098611842	0	0	170939199	197569704	88010513
Ces5a	203013616	-343783649	175248456	-19616929	004979825	098611842	0	0	769226395	098784852	251458609
Tnnc2	110869202	-3374164704	167861689	-20100862	004442206	197223683	291556122	096922715	085469599	572952142	251458609
Atp2a1	367109657	-3279426719	153963391	-21300042	003317127	11833421	777482992	096922715	427347997	177812734	176021026
Olfr1393	290856565	-2981528063	144803249	-20590201	003949231	098611842	0	096922715	256408798	790278816	502917217
DXBay18	675327732	-2560990519	097665647	-2622202	000873636	59167105	0	0	153845279	790278816	113156374
a	623947933	-2292451506	028902753	-79316027	216E-15	285974341	174933673	174460888	107691695	987848521	104355323
Zfp965	322946412	-2069629609	100127618	-20669918	003873493	276113157	194370748	775381723	350425358	849549728	364614982
Serpina3n	490851233	-2018707904	098315065	-20533048	004004303	197223683	291556122	096922715	111110479	49392426	754375826
Tnk2os	977588668	-1998864388	068589604	-29142381	000356558	394447367	388741496	387690862	128204399	177812734	163448096
Xlr4a	819904247	-1843183927	077501914	-23782431	001739535	098611842	291556122	678459008	145298319	889063668	150875165
Gm31946	522318832	-1840507581	093772546	-19627361	004967685	098611842	194370748	387690862	854695994	592709112	100583443
Gm16299	103878224	-1469710373	064410688	-22817803	002250232	59167105	48592687	581536292	136751359	207448189	113156374
Cys1	154962016	-1421108474	056630541	-25094383	001209233	138056578	388741496	775381723	230767918	306233041	138302235
Hymai	107448782	-1416961088	065711327	-21563422	003105695	788894733	583112244	387690862	213673999	790278816	176021026
Mrip-ps	173710553	-1282934142	049417868	-25960936	000942904	986118416	777482992	12599953	196580079	266719101	276604469
Gm30541	13094232	-1229439656	056994638	-21571146	003099674	986118416	48592687	872304439	145298319	217326675	188593956
Xntrpc	266147574	-1183707415	04683829	-25272217	001149689	276113157	777482992	135691802	512817597	217326675	377187913
Gm14421	247497399	-1175845455	045687828	-25736515	001006316	207084867	97185374	155076345	529911516	256840615	238885678
Slc26a7	183146901	-1171366319	051138871	-22905596	00219889	11833421	106903911	116307258	401707117	217326675	138302235
Gm51579	277615835	-1143793515	038801654	-29477958	000320048	157778947	174933673	184153159	367519278	316111527	465198426
Col3a1	161657599	-1127960264	051053238	-22093804	002714819	986118416	97185374	106614987	153845279	237083645	276604469

Sh3d21	139522142	-1126144921	054481624	-20670179	003873247	59167105	97185374	106614987	170939199	22720516	176021026
Gm48551	239783405	-1084185573	046674619	-2322859	002018673	108473026	106903911	242306788	188033119	365503953	427479634
A830035O19Rik	152061499	-1076081324	054251726	-19834969	004731195	138056578	388741496	116307258	205127039	187691219	226312748
Srrm4os	321337668	-0980439848	039747018	-24667004	001363644	167640131	165215136	319844961	547005436	464288805	264031539
Trim56	686377313	-0859966848	024654613	-34880566	000048655	404308551	505363945	552459478	820508154	96809155	867532199
Gpld1	46758636	-0813683084	03006644	-27062834	000680409	424030919	310993197	281075875	521364556	651980024	616073591
D830031N03Rik	152819732	-0766964162	018962102	-40447212	524E-05	867784206	109819473	143445619	204272343	210411735	162190802
Ccm2l	310022506	-0752419396	035604582	-21132657	003457804	266251972	22352636	203537702	384613197	405017893	377187913
Gm3257	462347147	-0733727902	030451858	-24094685	001597578	404308551	252681972	387690862	606834156	632223053	490344287
Rbbp9	10045789	-0720566109	007128936	-10107624	511E-24	766214009	758045917	755027953	130341139	129309371	115168043
Ahcy	185190634	-0685008717	005158826	-13278384	309E-40	142099664	14432028	13956871	224785046	229279642	231090461
LOC115486128	217328154	-0655507014	014306361	-4581927	461E-06	187362499	174933673	144414846	278630894	255852767	262774246
Crip1	728680418	-0629197985	027040832	-23268441	001997357	700144076	690016155	329537232	982900393	839671242	829813408
Trank1	64078423	0589896401	008127324	725818771	392E-13	760297299	784285968	765689452	505980029	525535413	502917217
1500002C15Rik	127672964	0592481268	020508962	288888962	000386605	162709539	172989966	123091849	863242954	889063668	132015769
Krt90	712503918	0619050414	024218553	255609987	001058527	769172365	101072789	804458538	538458476	523559716	628646521
Ntrk1	584646705	0621064714	027031187	229758581	002158537	779033549	621986393	717228094	435894957	375382438	5783548
Prima1	536605627	0635284748	027703239	229317864	002183772	660699339	612267856	678459008	333331438	444531834	490344287
Sst	399380946	0636755884	032314377	197050337	004878071	522642761	553956632	37799859	290596638	286476071	364614982
Gm17202	5832209	0670380091	026528769	252699286	001150438	729727628	67057908	75599718	487176717	503802745	352042052
Cldn1	385526274	0671790038	032961889	203808112	004154182	364863814	495645407	562151749	290596638	335868497	264031539
Muc2	498763731	0681229274	030540867	223054986	002571096	532503945	553956632	765689452	444441917	444531834	251458609
1700048O20Rik	410232708	0681733073	010716456	636155323	200E-10	530531708	495645407	489459713	332476742	277585434	335697242
Gm38745	607615391	0684952001	025831757	265158889	00080114	818478286	767764454	659074465	418801037	503802745	477771356
Plekha2	625897719	0691537722	027005618	256071795	001044561	690282891	621986393	100799624	512817597	444531834	477771356
Gm26721	514251272	0693517121	028017737	247527886	001331322	640976971	573393706	688151279	384613197	345746982	452625495
Car15	471964596	0695591156	030395133	22884952	00221087	621254602	660860543	474921305	376066237	434653349	264031539
Gm39529	626251314	0703608714	025452068	276444613	000570195	759311181	855231291	717228094	538458476	434653349	452625495
Cd34	322861108	07501554	036457585	205761134	003962746	35500263	398460033	455536762	162392239	276597586	2891774
Zfp968-ps	425246991	0751818113	036179905	207799914	003770944	82833947	379022958	387690862	247861838	306233041	402333774
Piezo2	299790584	0757311404	038195126	198274358	004739608	35500263	408178571	358614047	145298319	217326675	314323261
Rps27a-ps3	351371799	0759722213	034511118	220138392	002770886	394447367	563675169	368306318	273502718	256840615	251458609
Gm36513	271608856	0789297221	038527531	204865766	00404956	404308551	320711734	310152689	230767918	187691219	176021026

Gls2	346357791	0792369005	034960688	22664571	002342341	325419077	456771258	533074935	239314878	24696213	276604469
Gm3317	572110436	0796010691	027762013	286726579	000414035	59167105	690016155	901381253	470082797	414896379	364614982
Myh7b	453565457	0808431772	029797006	271313088	000666508	621254602	534519557	571844021	290596638	325990012	377187913
Gm46178	503328645	0814348664	032530251	250335811	00123021	788894733	573393706	571844021	547005436	237083645	30175033
Tm6sf2	303125795	0823081174	036154712	2276553	002281293	414169735	369304421	37799859	196580079	24696213	213739817
Erdr1	422866312	0823821241	005737541	143584375	943E-47	543647083	589429293	487908949	29016929	315617602	310425652
Lbp	297341837	0835629879	037339747	223790985	002522693	345141446	466489795	329537232	179486159	237083645	226312748
Jak3	345051551	0844092679	034237188	24654264	001368503	453614472	456771258	416767676	256408798	197569704	2891774
Gm15564	962840026	0875757902	024339325	35981191	000032053	159751183	106903911	108553441	905977754	553195171	565781869
Gm3788	114388319	0907063259	022798965	397852816	693E-05	164681776	172989966	111461123	863242954	96809155	540636008
Gm15520	233192238	0912359313	041881133	217844947	002937259	325419077	310993197	281075875	170939199	197569704	113156374
Gdf1	573589064	091991238	032150765	28612457	00042198	453614472	89410544	891688982	290596638	345746982	565781869
Gramd2	204545365	0928841187	045199354	205498774	003988019	285974341	301274659	213229974	119657439	118541822	188593956
Gipr	101162847	0935974217	020350485	459927239	424E-06	142001052	130228401	12599953	717944635	602587598	766948756
Gm19378	985393476	0956992451	021059215	4544293	551E-06	135098223	116622449	13956871	717944635	74088639	540636008
C3ar1	191531372	0962537816	045612566	211024701	003483709	246529604	281837585	232614517	136751359	138298793	113156374
0610009E02Rik	298291137	0975411356	048916747	199402333	00461495	432905985	44705272	306275781	25811819	889063668	256487781
Grik2	548764752	0982229651	009568829	102648886	101E-24	749449996	683213179	754058726	398288333	332904951	374673327
Gm27021	243443863	0985106217	047137381	2089862	00366302	216946052	544238094	213229974	222220958	138298793	125729304
Gm36266	41683783	0992691503	03232499	307097232	000213363	522642761	573393706	571844021	358972318	197569704	276604469
Gm5763	226066147	0992760756	042689969	232551296	002004455	276113157	340148809	290768146	188033119	148177278	113156374
H2-Q6	494675355	1041478448	029049212	358522099	000033679	719866444	660860543	610613107	290596638	296354556	389760843
Gm10443	213831371	1107003897	045752038	241957287	001553875	335280262	359585884	184153159	162392239	128420308	113156374
Gm12397	168816035	1107948315	050150531	220924541	002715758	207084867	310993197	174460888	111110479	108663337	100583443
1700003F12Rik	248432531	112557313	043697374	257583702	000999978	335280262	466489795	222922245	145298319	207448189	113156374
Hbb-bs	11772754	1140545509	014571684	782713583	499E-15	152355295	19096926	14276716	788029707	745825633	668879899
Xlr3b	219119813	1146510402	014371348	797775143	149E-15	313585656	295443537	298521963	143588927	149165127	114413667
Tdrd1	16503172	115854736	053002113	218585127	002882649	138056578	252681972	290768146	111110479	592709112	138302235
Plcg2	145652358	1163442623	054814196	212252062	003379404	256390788	145778061	203537702	769226395	128420308	628646521
Hba-a2	501720727	1202165829	011168121	107642621	508E-27	730713746	775539284	594136245	312818734	323026466	274089883
Cdh5	168307235	1204618336	049261598	244534972	001447117	23666842	242963435	222922245	854695994	108663337	113156374
Gm15710	168714936	1210708952	057251681	211471336	003445439	325419077	116622449	271383603	145298319	128420308	251458609
Zfp69	122579734	1229561676	018495038	664806255	297E-11	166654012	172018112	177368569	735038555	780400331	678938243

Hbb-bt	282421105	1247091479	012985074	960403802	769E-22	439808814	396516326	355706365	167520415	163982854	170991854
Otog	194809159	1258526297	048391376	260072436	000930272	197223683	379022958	25199906	136751359	128420308	754375826
1700030C10Rik	229488871	1258813237	045735211	275239406	000591613	23666842	340148809	387690862	119657439	790278816	213739817
Gm8364	10831218	1258867043	064081498	196447816	004947467	108473026	22352636	12599953	769226395	395139408	754375826
Prr33	116569557	1267000659	060712716	208687857	003689911	138056578	184652211	174460888	102563519	49392426	502917217
Gm52512	146611419	1269298029	053423896	237589941	001750623	177501315	242963435	203537702	940165594	987848521	628646521
Hba-a1	773265618	129484257	019177142	675200997	146E-11	997951837	12857625	10138116	456407661	523559716	362100396
D130051D11Rik	120921415	1297346542	05937269	21850897	002888227	147917762	136059524	232614517	769226395	691493964	628646521
Sox1	18629262	1298532097	050948135	254873332	001081149	23666842	310993197	25199906	102563519	177812734	377187913
Crybb1	152905108	131639919	053986696	243837704	001475338	216946052	204089285	232614517	111110479	395139408	113156374
Gm4737	111625283	1427374273	006583062	216825291	300E-104	160835914	164534838	163024007	617090508	605551143	59092773
Nkx6-1	908783062	1429773695	068598379	208426747	003713584	108473026	155496598	135691802	683756795	395139408	377187913
Gm1859	825003696	1449913852	072653696	19956505	004597198	128195394	106903911	12599953	512817597	197569704	628646521
Usp18	171882181	1483730162	052013452	285258931	000433646	216946052	26240051	281075875	598287196	148177278	628646521
Xlr3a	300167296	1498719675	039952073	375129394	000017592	493059208	340148809	50399812	222220958	128420308	113156374
Igfbp7	275599036	1528599384	041078634	372115438	000019831	443753287	349867346	426459948	111110479	108663337	213739817
Rplp2-ps1	170278535	154828936	05269014	293848027	000329826	305696709	252681972	203537702	119657439	395139408	100583443
Gm38651	767896503	1612757792	079347728	203251919	004210112	128195394	136059524	872304439	769226395	197569704	125729304
Gdnf	134378155	1639144747	058840459	278574432	00053405	167640131	165215136	281075875	854695994	691493964	377187913
E130112N10Rik	657661815	1646620106	083400729	197434737	004834226	986118416	106903911	969227154	598287196	197569704	125729304
Wnt4	824937478	1763197008	073479326	239958244	001641378	147917762	116622449	116307258	341878398	296354556	502917217
Aplnr	723258285	189564597	081163021	233560303	001951194	887506575	777482992	174460888	256408798	296354556	377187913
Gm33272	500165673	1924715692	01001497	192183878	260E-82	813547693	785257822	778289405	210255215	221278069	192365836
LOC108169009	113325838	1990501039	070175941	283644368	00045619	216946052	184652211	145384073	341878398	987848521	0
Gm14527	583966678	2024012049	100751184	200892136	004454547	59167105	174933673	484613577	085469599	592709112	0
Gm40260	518981036	2091155728	095214955	219624714	002807426	690282891	874668366	969227154	256408798	197569704	125729304
Tceal7	593763221	210830167	091496365	230424636	002120882	788894733	116622449	969227154	341878398	296354556	0
Eif3s6-ps2	481410015	2170291626	103089017	21052598	00352687	690282891	874668366	775381723	170939199	0	377187913
Gm46767	63155629	2179201201	08767997	248540368	001294046	11833421	874668366	106614987	341878398	197569704	125729304
Fgf17	121200181	2315745709	064333912	359957237	000031874	177501315	213807823	213229974	427347997	296354556	502917217
Gm9029	579168077	2324717056	099503521	233631637	001947476	788894733	388741496	174460888	256408798	296354556	0
Gm52788	538301088	2449557078	099146172	24706522	001348669	108473026	116622449	484613577	170939199	197569704	125729304
Tie1	889132271	245376677	076878544	319174458	000141416	167640131	116622449	164768616	170939199	296354556	377187913

Cd93	147394848	2512762475	060738272	413703321	352E-05	285974341	242963435	222922245	256408798	691493964	377187913
Agtr2	363707863	2579836199	127161893	20287809	004248061	986118416	194370748	678459008	0	197569704	125729304
Wbscr25	334935465	2620751953	131116931	199878988	004563109	394447367	680297618	678459008	256408798	0	0
Gm45351	195592503	2666588361	108349082	24611084	001385085	424030919	340148809	25199906	119657439	0	377187913
Rpl17-ps9	351458325	2697834988	129596125	208172505	003736759	493059208	874668366	484613577	256408798	0	0
Gm11832	274836319	2895991739	146103091	198215638	004746174	59167105	291556122	581536292	085469599	098784852	0
Robo4	297700056	2905024709	142414893	203983211	004136705	59167105	291556122	678459008	0	098784852	125729304
Gm39332	308499674	3039053967	146961576	206792419	003864715	197223683	874668366	581536292	0	197569704	0
Gm10136	32981366	3077372322	138417936	22232468	002619916	493059208	48592687	775381723	0	098784852	125729304
Gm41292	469763213	3123029756	121404281	257242145	001009899	157778947	680297618	290768146	170939199	098784852	0
Sox18	363441284	3233939445	137468582	235249348	001864802	986118416	291556122	678459008	0	098784852	125729304
Gm51990	210722423	3411417251	173022921	197165626	004864886	098611842	291556122	775381723	0	098784852	0
Gm41838	209479107	3417217691	169066667	202122497	004325648	394447367	583112244	193845431	085469599	0	0
C130080G10Rik	226152158	3534870708	166130771	212776398	003335666	59167105	48592687	193845431	085469599	0	0
Gm2832	241311189	3636042788	164685437	220787147	002725323	197223683	777482992	387690862	085469599	0	0
Actb-ps1	256989486	3732721975	173047196	21570543	003100143	0	97185374	484613577	085469599	0	0
Cym	265125904	3739730789	158692636	235658747	001844372	59167105	388741496	484613577	0	0	125729304
Cryge	260766492	3740371102	161498871	231603546	002055633	59167105	680297618	193845431	0	098784852	0
Gimap4	292748766	391849816	155029273	252758598	001148497	493059208	583112244	581536292	0	098784852	0
9430018G01Rik	146034668	3960861239	189826984	208656386	003692758	197223683	194370748	484613577	0	0	0
Cryga	146359965	3963461933	187525992	211355338	003455343	295835525	291556122	290768146	0	0	0
Gm33513	146403742	3963810788	188382334	210413085	003536703	295835525	388741496	193845431	0	0	0
Gm34196	146641487	3965697373	188324835	210577505	00352239	394447367	291556122	193845431	0	0	0
Gm7335	942144613	4065674355	0315942	128684201	678E-38	181445789	177849234	175430115	153845279	889063668	628646521
Gm2546	162319783	4113296437	183378127	224306819	002489242	197223683	48592687	290768146	0	0	0
Dmkn	178711314	4251846678	177112846	240064274	001636631	295835525	388741496	387690862	0	0	0
LOC108168367	178842643	4252695125	182780746	232666472	001998312	295835525	680297618	096922715	0	0	0
Lamtor3-ps	179555877	4257403178	181655901	234366358	001909539	59167105	388741496	096922715	0	0	0
Rpl32-ps	195234174	4379027015	174815348	250494426	001224707	394447367	583112244	193845431	0	0	0
Gm4792	419919772	5118092188	060821866	84148885	393E-17	808617101	874668366	765689452	256408798	197569704	251458609
