# Supplementary material for: Zeb2 Controls Retinal Physiological and Pathological Angiogenesis by Regulating Astrocyte Proliferation and Differentiation
Source: Cell Prolif. 2026 May 26:e70236. Online ahead of print. doi: 10.1111/cpr.70236 (PMC13325830; doi:10.1111/cpr.70236)
Supplement: Supplementary file 2 — Table S2: List of genes differentially expressed in P17 OIR Zeb2CKO retinas as determined by RNA‐seq analysis [file CPR-9999-e70236-s002.pdf]

Table S2. List of genes differentially expressed in P17 OIR Zeb2 CKO retinas as determined by RNA-seq analysis

gene	baseMean	log2FoldChange	lfcSE	stat	pvalue	CKO1	CKO2	CKO3	Control1	Control2	Control3
Gm2026	247525118	-6819875442	077934313	-87507994	212E-18	0	290159236	100813846	473067778	466153721	532946233
Gm18835	238171114	-4760713788	16688738	-28526506	000433563	0	0	0	7692159	308031093	351779692
Gm30294	216243332	-4651177374	170007321	-27358689	000622158	0	0	0	2564053	513385155	527669538
Crygn	199691167	-4508796249	174950705	-2577181	000996098	0	0	0	64101325	205354062	351779692
Gm36533	188070843	-4438039151	192420677	-23064253	002108688	0	0	0	5128106	0	615614461
Gm3776	171614438	-4306717045	180989263	-23795428	001733413	0	0	0	38460795	205354062	439724615
Rpl35a-ps2	144194141	-4075020951	205279529	-19851083	004713242	0	0	0	0	513385155	351779692
Gm4131	146553733	-4059810627	192956895	-21039987	003537856	0	0	0	5128106	102677031	263834769
Capn11	283392848	-3863689743	053449502	-7228673	488E-13	229832049	507778662	302441537	56409166	513385155	518875046
Dcstamp	256211667	-3826738423	160702628	-23812544	001725379	0	0	100813846	5128106	308031093	615614461
Fmr1nb	254868434	-3784915734	163120302	-23203217	002032348	114916024	0	0	7692159	205354062	439724615
Mip	637873379	-37776044	118507302	-31876554	000143431	0	217619427	0	192303975	13348014	351779692
Crygs	115572767	-3775934618	027469626	-13745854	539E-43	919328196	137825637	241953229	192303975	227943009	226018452
Gm30601	246464882	-3757180773	161498657	-23264471	001999471	0	0	100813846	5128106	513385155	351779692
Cryba1	187087872	-3698923097	021483762	-17217297	197E-66	160882434	319175159	302441537	32948081	335753892	379042618
Fcrla	222840786	-3636344336	166269286	-21870211	002874098	0	072539809	0	38460795	616062187	263834769
Gm32092	220843504	-36005636	166872459	-2157674	003095319	0	0	100813846	2564053	616062187	351779692
LOC1154903	529724971	-34938852	118656996	-29445253	000323451	0	217619427	0	15384318	718739218	703559384
Gm34194	200817245	-348301062	169289476	-20574289	003964499	0	072539809	0	38460795	308031093	439724615
Gja3	199018326	-3474032208	170338473	-20394877	004140138	0	072539809	0	2564053	513385155	351779692
Oaz3	199018326	-3474032208	170338473	-20394877	004140138	0	072539809	0	2564053	513385155	351779692
Cryaa	591511486	-3402987547	014053752	-24214086	158E-129	735462557	103731927	127025445	112049116	116127722	962996907
Crygd	219531919	-3341905027	018384726	-18177616	776E-74	333256471	384460987	46374369	376915791	391199488	430930123
E230016K23F	513519043	-3317233982	112351005	-29525628	000315148	114916024	072539809	100813846	7692159	123212437	791504307
Hao1	353562476	-3307794266	136548094	-2422439	001541671	114916024	0	100813846	5128106	513385155	87944923
Crygb	285134264	-3293896566	016358182	-2013608	356E-90	413697688	500524681	665371381	517938706	525706399	509201104
Crybb2	138168213	-328551273	022593022	-14542157	655E-48	183865639	275651274	302441537	243585035	239237482	269990914
S100a8	304982663	-3186054047	141586438	-22502537	002443285	0	072539809	100813846	5128106	616062187	527669538
Crygc	172175307	-3160574483	019848032	-15923868	433E-57	287290061	319175159	433499536	314096492	308031093	306927781
Vmn2r2	281160203	-3066584617	14463078	-21202849	003398202	0	072539809	100813846	38460795	513385155	615614461

Mmp3	407377146	-3037886437	125617463	-24183631	001559051	0	145079618	100813846	10256212	205354062	967394153
Rpl31-ps9	443736069	-3022973328	121046951	-2497356	001251233	0	0	302441537	10256212	718739218	615614461
Crygf	103127693	-3014156839	026723876	-11278891	167E-29	114916024	217619427	342767075	178201683	203300522	169733701
Gm51974	268853079	-2950728604	145617859	-20263508	004272885	114916024	072539809	0	38460795	513385155	527669538
Gm4737	169522458	-2949871087	019200999	-15363113	289E-53	367731278	442492834	342767075	297430148	308031093	29637439
Cryba2	348598309	-2892892212	041768244	-69260566	433E-12	574580122	797937898	11089523	48717007	62632989	729942861
Gbp10	665655882	-2815657348	097989638	-28734236	000406049	0	435238853	0	115382385	143747844	967394153
Cryga	188542713	-2779243833	019044194	-14593654	308E-48	333256471	602080414	473825074	314096492	33062004	345623547
Cryge	280479908	-2771846576	016227773	-17080881	206E-65	505630508	819699841	79642938	473067778	468207262	529428436
Dmp1	446422497	-2767557558	113266771	-24433976	00145497	0	217619427	100813846	5128106	616062187	123122892
Cyp2b27-ps	114431423	-2720875296	070129809	-387977	000010456	229832049	435238853	201627691	243585035	215621765	140711877
Crybb1	550226866	-2550124157	031675952	-8050663	823E-16	172374037	116063694	201627691	935879345	995967202	87944923
Cryba4	59131106	-2533333242	030255473	-83731404	561E-17	229832049	16684156	131057999	101280093	995967202	101136661
Ifi209	516694181	-2504324897	098514322	-25420922	001101911	114916024	145079618	201627691	10256212	821416249	791504307
Mgst2	416565915	-2413934841	121111702	-19931475	00462453	0	0	403255382	10256212	718739218	351779692
Gm4951	472847846	-2398204201	104949324	-22851069	002230656	0	145079618	302441537	5128106	821416249	105533908
Foxd2	440817814	-2386040079	107838149	-22126122	002692439	0	290159236	100813846	64101325	821416249	791504307
Cd200r1	428875385	-2224958658	107405485	-20715503	00383074	0	145079618	302441537	5128106	821416249	791504307
Asb14	51347023	-2209909875	09680869	-22827598	002244452	114916024	217619427	201627691	115382385	513385155	87944923
Ccl7	186718196	-2183891997	052797632	-41363446	353E-05	574580122	65285828	806510765	23076477	246424875	439724615
Clec4n	133802359	-2167470512	06048688	-3583373	000033919	344748073	507778662	604883073	192303975	174550953	290218246
Clec4d	557519658	-2157087776	095713847	-22536841	002421605	229832049	362699044	0	64101325	123212437	87944923
Zfp990	847452502	-2099660728	075426854	-27837045	00053742	114916024	507778662	302441537	115382385	13348014	167095354
Mmp10	140625984	-2068187977	05847355	-35369633	000040476	229832049	65285828	705696919	243585035	195086359	246245784
Iigp1	403291724	-2058516622	033917148	-60692504	129E-09	137899229	16684156	161302153	628192985	657132999	668381415
Epsti1	512339319	-2018210418	095246699	-21189295	003409642	114916024	362699044	100813846	89741855	718739218	87944923
LOC10086232	927541433	-1936990709	097099941	-19948423	004606008	689496147	362699044	100813846	2564053	184818656	0
Gm37013	125458278	-1921836102	074133922	-25923842	000953133	0	435238853	11089523	346147155	102677031	149506369
H2-Aa	170978666	-1906568767	054927735	-34710493	000051843	229832049	123317675	604883073	269225565	277227984	272629261
Mrc1	534752363	-1886904813	029039335	-64977549	815E-11	229832049	239381369	211709076	858957755	841951655	826682276
Gm3317	942720764	-1856256074	081764424	-22702491	002319248	0	943017516	201627691	166663445	205354062	791504307
Gm16174	616853023	-1848837632	093555878	-19761854	004813378	0	290159236	504069228	38460795	16428325	87944923
1500035N22I	68960244	-1823045456	027862756	-65429474	603E-11	321764868	319175159	272197383	103844146	831683952	135435181

Gm36538	670238216	-1778759725	082550378	-21547566	003118089	114916024	362699044	403255382	141022915	102677031	703559384
Kcnj15	658057218	-1766527311	081726542	-21615099	003065597	114916024	362699044	403255382	89741855	102677031	1143284
Cxcl10	328636168	-1758701729	037667364	-46690332	303E-06	114916024	188603503	141139384	423068745	523652859	580436492
Ms4a7	821312666	-173503421	075042685	-23120631	002077421	114916024	580318471	403255382	141022915	92409328	149506369
Oasl1	690311821	-1710724893	082094516	-2083848	0037174	459664098	217619427	302441537	166663445	616062187	87944923
Gbp4	149699424	-1692408892	056478073	-29965769	000273029	459664098	108809713	504069228	269225565	256692578	167095354
Gm16464	801609121	-1656731476	073983967	-22393115	002513566	344748073	580318471	201627691	141022915	112944734	1143284
Crybb3	191013329	-1655348972	016725123	-98973799	427E-23	103424422	855969745	877080456	320506625	294683079	254160827
Galnt12	12168317	-1650168766	062391696	-26448532	000817264	459664098	870477707	403255382	115382385	256692578	184684338
Tmprss11e	244315014	-1647282012	047116947	-34961561	000047201	804412171	203111465	604883073	346147155	441511234	334190707
Taco1os	746046741	-1644325135	082670056	-19890214	004669884	0	362699044	705696919	12820265	718739218	140711877
Ch25h	212780903	-159858318	046789687	-34165289	000063425	689496147	15958758	806510765	30768636	308031093	351779692
Ccdc194	874701148	-1595335925	072706544	-21942123	002822015	689496147	435238853	201627691	7692159	174550953	140711877
Pgf	132427713	-1583997831	0190123	-83314371	799E-17	586071725	616588376	786347995	202560187	189952508	203152772
Lrr1	856017825	-1575750953	071617855	-22002208	002779123	229832049	435238853	604883073	166663445	123212437	967394153
Ccl2	697267188	-1566124117	02600106	-60233087	171E-09	287290061	391714968	373011229	910238815	101650261	120484544
LOC1081686	668202942	-1564133951	02577493	-60684315	129E-09	344748073	333683121	33268569	111536305	985699498	897038215
Timp1	15905427	-1547241846	017389603	-8897511	571E-19	850378581	848715764	735941073	220508558	22999655	260316972
S100a3	945528102	-1545623541	066585445	-23212634	002027263	344748073	580318471	504069228	12820265	16428325	131917384
Bcl3	166796084	-150214497	019585517	-76696724	172E-14	116065185	819699841	645208612	237174902	274147673	226897901
Gm36841	163976487	-1483422083	051346792	-28890258	000386437	919328196	943017516	705696919	30768636	225889468	193478831
Tnfrsf9	365667968	-1475766355	035711064	-41325186	359E-05	218340446	188603503	171383537	705114575	46204664	448519107
9330188P03F	936249395	-1458964605	022214316	-65676773	511E-11	51712211	457000796	534313382	117946438	141694303	151265268
Gm20482	187602677	-144916921	061126816	-23707585	001775162	114916024	181349522	0	269225565	287495687	272629261
Gm19951	820241323	-1445836692	07081487	-20417134	004117997	459664098	362699044	504069228	141022915	112944734	105533908
Angpt4	178239039	-1431166401	050569097	-28301206	000465305	103424422	580318471	131057999	30768636	266960281	202273323
Hbb-bt	510071818	-1405418048	013620462	-10318431	582E-25	209147165	293060828	334701967	687166204	747488786	788865959
1700030C10F	3009158	-1397919918	045103356	-309937	000193933	459664098	268397293	171383537	346147155	472314343	501286061
Lilr4b	140863686	-138670057	057016189	-24321172	001501085	344748073	870477707	11089523	15384318	195086359	263834769
Gm4070	774751269	-1382550023	062578189	-22093161	002715266	149390832	420730892	715778304	119228464	139640762	773915322
Fgr	321447444	-1364333699	038213876	-35702573	000035663	919328196	217619427	22179046	499990335	431243531	466108092
Pf4	192437679	-1363031477	047111645	-28931944	000381345	137899229	116063694	705696919	243585035	287495687	299012738
Ccbe1	111129303	-1362670073	061971176	-21988772	002788665	459664098	870477707	504069228	166663445	123212437	193478831

Fam90a1b	116547095	-136164328	059098992	-23040042	002122241	689496147	580318471	705696919	141022915	184818656	175889846
A730094K22F	203091785	-1358155315	045682463	-29730343	000294871	804412171	108809713	151220768	30768636	29776339	272629261
Gm12866	107275412	-1353968866	065020436	-20823743	003730829	344748073	101555732	403255382	115382385	184818656	167095354
Rsad2	246974331	-1353462462	041524435	-32594362	000111634	137899229	174095541	100813846	346147155	379905015	3429852
Ifitm1	566372422	-1352427589	029086364	-46496964	332E-06	264306856	319175159	373011229	782036165	657132999	100257212
Ifi204	30680662	-1339330397	04126291	-32458457	000117102	241323651	137825637	151220768	35896742	318298796	633203446
Gm34292	155192422	-1302787658	054776701	-23783609	00173898	574580122	145079618	604883073	166663445	246424875	255040277
Ifi211	289163184	-130130516	040550238	-32091184	000133143	137899229	16684156	191546307	5128106	46204664	263834769
Enpp6	131871106	-1296909308	05915919	-21922364	002836244	344748073	725398089	120976615	192303975	13348014	237451292
Msr1	350338415	-129035699	034577809	-37317488	000019016	229832049	210365446	171383537	474349805	46204664	554053015
Tgm2	631640869	-127851278	010066613	-12700525	587E-37	396460284	32642914	387125167	902546656	894316941	882967027
Gdf1	246724399	-1278108404	045420573	-28139416	000489381	689496147	130571656	231871845	28204583	441511234	325396215
Ccl11	269467295	-1276684259	043409006	-29410585	000327093	149390832	116063694	211709076	38460795	236157172	518875046
Kbtbd13	161583014	-1264301879	053582074	-23595613	001829656	919328196	130571656	604883073	141022915	308031093	237451292
Isg15	305849216	-1256160721	037512659	-33486315	000081212	172374037	232127388	131057999	43588901	441511234	42213563
Sp100	181525673	-1256011843	049382566	-25434317	001097695	114916024	870477707	120976615	320506625	16428325	281423754
Ifi27l2a	165460459	-1244076609	053601927	-2320955	002028927	459664098	116063694	131057999	141022915	277227984	281423754
Gm43305	177256802	-124175747	016006091	-77580308	863E-15	976786208	110985908	106862676	242303008	239237482	266473117
F13a1	393831938	-1235919369	032711355	-37782579	000015793	183865639	253889331	262115998	53845113	605794483	518875046
Mfap4	119707811	-1230824338	020052524	-6138002	836E-10	884853388	660112261	614964458	162817365	159149398	180287092
Rtp4	474672008	-1218711148	029697794	-41037093	407E-05	287290061	261143312	312522921	58973219	667400702	729942861
Gm20742	452385019	-1218107728	010804557	-11274018	176E-29	266605177	264044904	286311321	624346905	603740943	669260864
Gm12663	766980487	-1209086649	024952758	-48455031	126E-06	459664098	471508758	453662305	129484676	831683952	109051705
Slit3	141713228	-1199399537	060794348	-197288	004850924	103424422	725398089	806510765	30768636	215621765	703559384
Esm1	136490013	-1190512879	018432049	-64589284	105E-10	746954159	81244586	937568764	183329789	176604493	209308917
Ccl28	540112565	-118397845	02961781	-39975219	640E-05	413697688	253889331	33268569	705114575	89329017	641997938
Dnaja1-ps	281091856	-1173330579	038750241	-3027931	000246234	206848844	181349522	131057999	346147155	451778937	369368677
Gm21378	172315121	-1164188249	050845282	-22896682	002204056	160882434	101555732	604883073	20512424	215621765	290218246
Gm40781	241313334	-115694666	040838488	-28329811	000461161	160882434	137825637	151220768	320506625	369637312	30780723
Spidr	612169624	-1153891409	009654611	-11951713	636E-33	361985477	399694347	376035644	810240748	836817803	888243722
Serping1	702963501	-1148968094	010246284	-1121351	350E-29	37462624	475861146	455678582	101408296	972351484	92518059
Mmp13	16449072	-1142991136	050575448	-22599724	002382297	574580122	116063694	131057999	23076477	205354062	246245784
Mmp12	804605722	-1142894911	024056039	-47509689	202E-06	379222881	616588376	493987843	112818332	105757342	115207849

Ms4a6d	417573217	-1135147916	031642475	-35874182	000033397	229832049	304667197	241953229	64101325	533920562	554053015
Tlr13	375904477	-1131989348	033242469	-34052505	000066103	183865639	24663535	272197383	551271395	482582046	518875046
Ogn	12627554	-1120971722	018633814	-60157933	179E-09	792920569	761667993	836754918	173073577	158122628	187322686
Ifi207	183126242	-1118977855	047138518	-23738079	001760571	137899229	108809713	100813846	28204583	205354062	263834769
Hbb-bs	572669841	-1115256812	01033168	-10794534	365E-27	28211884	390771951	411824559	789856526	745845954	815601216
Ifit1	582948178	-1114018657	027252168	-40878166	435E-05	356239676	369953025	383092613	653833515	811148546	923421691
Bvht	145560812	-111256731	055048009	-20210855	004327092	689496147	580318471	151220768	20512424	205354062	184684338
Dio3	481813603	-1111336427	030906955	-35957487	000032346	402206086	261143312	262115998	576911925	605794483	782709815
Mlkl	247151075	-1111209888	043002583	-25840538	000976466	919328196	145079618	231871845	320506625	29776339	395752153
Lyz2	321340458	-1108165232	012279318	-90246481	180E-19	18616396	213992436	209692799	424350771	443564774	450278006
Pla1a	445258677	-109963134	033303375	-33018615	000096045	229832049	232127388	393173998	46152954	739274624	615614461
Nlrc5	364816352	-1095751551	033449758	-32758131	000105358	252815254	232127388	211709076	56409166	46204664	466108092
Alas2	60562296	-1087933528	026783106	-4062014	487E-05	413697688	377207006	373011229	782036165	975431795	712353876
Nox4	112404112	-1082831584	020002172	-54135699	618E-08	827395376	681874204	665371381	142304941	145801384	168854252
C430049B03I	340512071	-1080198158	034316536	-31477483	000164533	218340446	217619427	22179046	41024848	482582046	492491569
Hba-a1	228449802	-1078328956	025640706	-42055354	260E-05	989426971	163432189	178037251	304224888	29776339	328298398
Gm41608	680626541	-1076936593	01149096	-93720332	711E-21	360836317	454099204	497012259	874342073	886102778	101136661
Gm39378	206260451	-1070831515	04839878	-22125176	002693092	689496147	145079618	181464922	33332689	174550953	334190707
Xist	333309536	-1070081126	005149642	-20779718	660E-96	222672781	211424527	211164681	441440185	44497145	468183592
Ccr1	186846322	-1068818407	049273689	-21691463	003007158	689496147	145079618	141139384	33332689	256692578	175889846
Hba-a2	156664614	-1066988101	028215715	-37815385	000015586	6561705	124550852	113415576	205637051	199296117	231471037
Gm4792	100483857	-1065512441	027492897	-38755917	000010637	427487611	753688614	767193365	125638597	130810538	151617047
A2m	202666777	-1057074588	006380269	-16567868	119E-61	133072756	132602771	128940908	277302332	276817276	267264621
Gm21028	146902967	-1049751351	051796376	-20266888	004269425	103424422	943017516	90732461	17948371	184818656	2286568
Zfp981	338064243	-1037269825	034752355	-29847469	000283813	241323651	195857484	231871845	397428215	513385155	448519107
Bmp2	338919428	-1036375718	035201299	-29441406	000323853	218340446	232127388	211709076	53845113	472314343	360574184
Fgf2os	501606428	-1027849076	010838146	-94836245	246E-21	314869907	334408519	341758936	624346905	651999147	74225515
Vdr	216446063	-1027035559	043164911	-23793297	001734415	137899229	130571656	161302153	269225565	256692578	3429852
Bfsp2	44443488	-1026877555	030821874	-33316519	000086332	229832049	282905255	362929844	628192985	564723671	598025476
Cldn2	600991087	-1024541031	026386389	-38828392	000010324	367731278	442492834	373011229	833317225	718739218	870654738
Fgf23	482247241	-1024534712	029094347	-35214219	000042924	287290061	340937102	322604306	653833515	585259077	703559384
Gpihbp1	25754841	-1022331772	041646362	-24547925	001409659	137899229	203111465	161302153	43588901	369637312	237451292
Lilrb4a	839771217	-1018161349	022612339	-45026804	671E-06	4711557	602080414	584720304	116664411	102677031	118725646

Baat	161204617	-1014586216	051384572	-19744958	00483254	574580122	108809713	151220768	243585035	195086359	211067815
Hmox1	195140565	-1008506349	008041268	-12541633	442E-36	114226528	14058215	133376718	263584648	252688174	266385172
Casp12	368223507	-1006666955	0346345	-29065439	000365446	183865639	224873408	322604306	551271395	513385155	413341138
Edn2	797772031	-0988085534	008378351	-11793317	423E-32	532061193	53897078	532297105	107946631	107400175	102983505
Tfpi2	290465564	-0979332664	039832784	-24586096	001394762	206848844	210365446	171383537	243585035	523652859	386957661
Csf2rb	187410562	-0978924573	046694751	-20964339	003604372	804412171	152333599	141139384	2564053	256692578	237451292
Antxr2	854950403	-0975358842	008114653	-12019724	280E-33	579176763	566535907	584720304	112433724	113252765	114240455
Pcolce	126433491	-0972514072	007483191	-12995982	129E-38	825097056	866125318	866999072	17563763	162332386	164808786
Gm46054	605398814	-0971711961	02641872	-3678119	000023496	390714483	362699044	473825074	884598285	790613139	729942861
Serpina3n	266806788	-0969747308	00678983	-14282351	282E-46	166628235	185194132	18902596	346916371	348280489	364795541
Eif4ebp1	137275257	-0967319416	017867265	-54139199	617E-08	861870183	103731927	877080456	17563763	190979278	179407643
Ccl12	385686843	-0958183791	035198212	-27222513	000648388	160882434	239381369	383092613	551271395	513385155	466108092
Mlf1	720174523	-0956560888	011791262	-81124558	496E-16	390714483	519385032	557500566	87177802	976458566	100521047
Lcat	968974968	-0953973774	022184235	-43002329	171E-05	540105315	710890127	725859688	115382385	117051815	151265268
Gm17501	183296038	-0952714414	047167111	-20198702	004339686	804412171	130571656	161302153	243585035	246424875	237451292
Igfbp3	709383038	-0948912044	00912314	-10401156	245E-25	449321656	496897691	504069228	964083928	897397252	944528473
Depp1	799590124	-0948501875	025943807	-36559857	00002562	333256471	703636146	584720304	99998067	106784112	110810603
Tagln	237054999	-0946912749	014995186	-63147783	271E-10	132153428	179898726	171383537	328198784	290575998	32011952
Btla	209878194	-0946423539	044402805	-21314499	00330521	114916024	145079618	171383537	217944505	266960281	3429852
Egr2	678604309	-0946369095	024779181	-38192105	000013388	413697688	471508758	504069228	833317225	995967202	853065753
Kcne4	331994706	-0944371883	035610433	-26519528	000800277	183865639	195857484	302441537	43588901	451778937	42213563
Ackr3	166209092	-093125282	016269094	-57240607	104E-08	105722743	114612898	121984753	22691869	224862698	203152772
Ncapg	285778486	-0928756851	038981213	-23825756	0017192	114916024	24663535	22179046	371787685	390172718	369368677
Cd72	296695423	-0926606732	036434547	-25432091	001098395	229832049	203111465	181464922	397428215	390172718	378163169
Bcl2a1b	332289383	-0922273516	035357128	-26084515	000909529	252815254	195857484	241953229	5128106	359369609	430930123
Kif14	185057501	-0916385133	015842357	-57843988	728E-09	134451749	134198646	115935922	241020982	221782387	26295532
Tnfaip2	358606399	-0915268128	035212548	-2599267	000934231	310273266	188603503	252034614	48717007	544188265	369368677
Bub1b	531941192	-0914541539	010825888	-84477276	297E-17	366582118	356170462	38510889	685884177	62838343	769518076
Cela1	601168808	-0914194236	027126046	-33701713	000075121	333256471	500524681	403255382	884598285	790613139	694764892
Gm13821	368348451	-0912785685	011840725	-77088668	127E-14	287290061	238655971	242961368	475631831	489769438	475782033
Mndal	375220292	-0910883989	032975104	-27623385	000573889	218340446	275651274	282278768	551271395	492849749	430930123
Rps27a-ps3	215678675	-0909113401	045129438	-20144576	004396151	804412171	210365446	151220768	294866095	266960281	290218246
Gm42788	224539625	-0908355343	045024916	-20174504	004364853	919328196	181349522	191546307	30768636	205354062	369368677

Litaf	314719227	-090575529	012097225	-74872982	703E-14	23327953	213992436	210700937	41024848	413788435	406305544
Slc16a4	480482758	-0905563617	030069688	-30115498	000259918	367731278	391714968	241953229	58973219	605794483	685970399
2900057B20F	14940951	-090278663	01755683	-51420821	272E-07	850378581	1138875	111903369	199996134	193032818	192599381
Lad1	394699431	-0898928754	011755454	-76469081	206E-14	294185023	270573487	262115998	521784785	553429198	466108092
Trim30a	538985416	-089587518	028504171	-31429617	000167248	459664098	391714968	282278768	679474045	646865296	773915322
Pbk	259529615	-0894697785	040369971	-22162458	002667467	229832049	130571656	191546307	320506625	29776339	386957661
Calca	376236175	-0894391899	033208851	-26932335	000707627	241323651	253889331	292360152	576911925	400440421	492491569
Srgn	141786691	-0885146203	018116185	-48859415	103E-06	896344991	104457325	104846399	158971286	187898967	204911671
Spp1	70089236	-0884507973	012676543	-69775173	300E-12	37462624	544773965	555484289	869213967	872754764	988500934
Adgrl4	94220873	-0884165884	008399917	-10525888	656E-26	710181031	621666162	657306273	125895002	121261574	119253316
Lcp2	104058865	-0879049317	020169051	-43584069	131E-05	666512942	725398089	806510765	135894809	125265978	143350224
Nlrp5-ps	317867927	-0877243145	035814321	-244942	001430865	218340446	261143312	191546307	346147155	441511234	448519107
5730507C01F	291602095	-0876940045	037899542	-23138539	002067574	229832049	152333599	241953229	320506625	400440421	404546646
Cebpd	148020514	-0874544709	007458317	-11725765	940E-32	104458666	101338113	107568373	204226821	187693613	182837495
Slc13a4	155384889	-0865070632	017117292	-50537822	433E-07	109170223	108809713	111903369	22691869	177631264	197876077
Socs3	446251297	-0861454339	010604655	-81233602	453E-16	299930824	321351353	327644998	605116508	56061659	562847507
Optc	813472675	-0856066482	011020206	-77681529	796E-15	463111579	645604299	624037704	103972349	104422541	106413357
Ube2l6	22762722	-0855651464	014438781	-59260643	310E-09	165479075	166116162	155253322	258969353	312138175	30780723
Abi3bp	104733093	-0853179952	020653443	-41309332	361E-05	827395376	631096337	79642938	125638597	130399829	146868021
Slc16a12	635137766	-0850219703	026374001	-32237039	000126544	402206086	384460987	57463892	87177802	821416249	756326338
Cxcl1	263342633	-0848657	040603927	-2090086	003661008	183865639	195857484	181464922	35896742	431243531	2286568
Kcne3	383512276	-084687632	032787707	-2582908	000979714	241323651	311921178	262115998	53845113	533920562	413341138
B2m	147352423	-0846860579	011804566	-71740088	728E-13	814754613	112436704	121783125	18140675	176296462	210716035
Klhl6	683732879	-0840928707	024396891	-34468683	000056712	51712211	435238853	524231997	858957755	852219358	914627199
Gm13136	280490535	-0838020079	038407013	-21819455	002911356	149390832	217619427	231871845	38460795	400440421	299012738
Fgf2	331814276	-0833189557	006732317	-1237597	353E-35	259940047	231329451	224915689	419991881	425185586	429523004
Angpt2	591059956	-0832434639	009409465	-88467799	901E-19	417145169	430886465	427450705	723062946	784452518	763361932
Bmp4	526129334	-0831424383	027352018	-30397186	000236799	390714483	391714968	352848459	66665378	677668405	677175907
Gbp3	924853392	-0829853233	022853904	-36311223	000028219	540105315	65285828	806510765	110254279	105757342	138952978
Tfpi	240053312	-0828233869	014335434	-57775292	758E-09	160882434	158862181	199611414	310250413	315218485	295494941
Lox	551275808	-0827953233	027050708	-30607452	000220787	390714483	413476911	383092613	79485643	657132999	668381415
Ggta1	121235092	-0827295979	020984787	-39423605	807E-05	632038135	884985669	109887092	155125206	142721073	167974803
Adgre1	688530384	-0825290821	024914649	-33124722	000092475	586071725	435238853	473825074	961519875	883022467	791504307

Gpx3	210702863	-0823962661	014903189	-55287673	322E-08	145943351	152333599	158277738	267943538	243344564	29637439
Ctla2b	47032846	-0823421331	03349428	-24583939	0013956	172374037	377207006	46374369	525630865	667400702	615614461
Rab38	635470267	-0817548424	026664657	-30660377	000216916	459664098	435238853	493987843	64101325	780345436	100257212
Lenep	403710484	-0815971863	034077494	-23944597	001664487	172374037	391714968	302441537	499990335	554455968	501286061
Zc3hav1	723299841	-081344608	023624614	-34432143	000057484	4711557	536794586	564557535	884598285	92409328	958599661
Depdc1a	284399871	-0810730821	039882772	-20327845	004207429	149390832	290159236	171383537	346147155	441511234	30780723
Myct1	585968665	-0808337085	026107369	-30962028	000196016	436680893	435238853	403255382	79485643	75981003	685970399
Il13ra1	126429376	-0807424418	018297749	-44126981	102E-05	965294605	964779458	826673534	162817365	152988776	167095354
P2rx7	467292667	-0807242401	030605599	-26375645	000835037	241323651	340937102	433499536	628192985	544188265	615614461
Ltbp1	271658518	-0801910808	01312812	-61083448	101E-09	212594645	191505095	190538168	365377552	323432648	346502997
Rarres2	164838827	-0800871932	016464826	-48641384	115E-06	126407627	118239889	115935922	229482743	187898967	211067815
Gm42048	755336719	-0799455971	008549376	-93510448	868E-21	567685161	537519984	550443597	94485353	945655456	985862587
Apold1	148046097	-0798152449	0174111	-45841585	456E-06	101126102	118965287	103838261	170509524	188925737	204911671
Cep55	328651471	-0797626665	035883309	-22228348	002622694	172374037	290159236	252034614	423068745	359369609	474902584
Accsl	26369658	-0796910164	040494695	-19679372	004907527	241323651	123317675	22179046	294866095	349101906	351779692
Crhbp	42825361	-0796054975	011282849	-70554429	172E-12	288439221	324252946	323612444	592296243	530840251	510080553
Nupr1	483364176	-0795922371	029651754	-26842337	000726963	222937087	426534076	409304213	656397568	542134724	642877387
Fcgr2b	546571767	-0795619688	027849363	-28568685	000427843	448172495	355445064	403255382	576911925	800880842	694764892
Gm17330	357531922	-0791951608	035135516	-22539917	002419669	206848844	348191083	22179046	48717007	379905015	501286061
Nudt6	421003836	-0791317754	012506467	-63272688	250E-10	364283798	279278264	285303183	496144255	526733169	574280347
Trim30d	302914294	-0790470158	035950187	-21987929	002789265	241323651	224873408	201627691	346147155	390172718	413341138
Olfml2a	331509971	-0789626181	039125818	-20181717	004357339	356239676	181349522	201627691	28204583	554455968	413341138
Niban1	256433954	-078814433	013434593	-58665291	445E-09	179268998	203836863	179448645	326916757	337807432	311325027
Zfp951	143256724	-078787113	017957728	-43873652	115E-05	101126102	108809713	105854538	157689259	177631264	208429467
Ctla2a	479447701	-0782499592	011832186	-66133138	376E-11	299930824	389538774	365954259	555117474	647892066	618252809
Prss56	101527034	-0782497051	008568263	-91325048	669E-20	79636805	761667993	680493458	133971769	12865432	122683168
Gm5954	355575434	-0779942296	0342108	-22798131	002261878	333256471	224873408	231871845	48717007	390172718	466108092
Zic4	497030645	-0774491916	030272264	-25584208	001051488	241323651	457000796	393173998	576911925	636597593	677175907
Tm4sf1	521355859	-0774353598	012504046	-61928244	591E-10	310273266	430886465	409304213	699986469	59347324	684211501
Ggt5	423003536	-0773444144	033692053	-22956278	002169717	195357242	435238853	292360152	576911925	554455968	483697076
4930523C07F	685330919	-0772061323	025472005	-3031019	00024373	494138905	580318471	443580921	74357537	821416249	10289556
Emp3	79082245	-0769688626	025807754	-29823929	000286005	367731278	602080414	776266611	103844146	975431795	984983137
Gstm2	313211784	-0766954669	03883546	-19748824	004828149	229832049	195857484	272197383	46152954	236157172	483697076

H2-Q7	124949056	-0766950152	018352947	-41788936	293E-05	953803003	943017516	877080456	164099392	143747844	164457006
Rbp1	179652261	-0766279667	010600427	-72287625	487E-13	104688498	150302484	14365973	218457316	226608208	23419733
Ccr5	526942591	-0764616542	029365525	-26037898	000921993	275798459	478762739	403255382	7692159	636597593	598025476
Fbln1	38436369	-0763065916	011413285	-66857694	230E-11	257411895	298138615	297400844	494862229	488742668	469625889
S100a11	486772578	-0761587311	015113044	-50392715	467E-07	248218613	422907086	409304213	593578269	616062187	630565098
Ctsc	13863187	-0760711782	007838129	-97052721	286E-22	942311401	108084315	106156979	170124917	171059934	182133936
Gm15983	980824494	-0760607983	021042073	-36147008	000030069	60905493	819699841	746022457	123074544	116025045	131917384
Tyrobp	140863149	-0758650182	021985013	-34507607	000055901	632038135	124043073	125009169	176919657	16428325	191719932
Icam1	525122988	-0758482247	028185966	-26909925	000712398	298781664	449746815	413336767	679474045	667400702	641997938
Sdc1	66450313	-0756204188	02496534	-30290162	000245352	425189291	558556529	493987843	782036165	821416249	905832707
Bub1	41818876	-0751849654	030959484	-24284954	001516162	367731278	297413216	272197383	551271395	492849749	527669538
Gm13707	476522403	-0751320775	031960717	-23507632	001873495	448172495	224873408	403255382	653833515	513385155	615614461
Lgals1	470070414	-0750221852	014685747	-51085033	325E-07	251666094	409124522	388133305	546143289	590392929	634962344
Ptprc	619515605	-07469337	025291625	-29532847	000314412	459664098	471508758	453662305	82049696	790613139	721148369
Tmem267	763302914	-0746890494	009240103	-80831405	631E-16	510227149	606432802	590769135	935879345	955923159	980585891
Gadd45b	611981816	-0745567812	009727532	-76645118	180E-14	435531733	444669029	492979705	728191052	764943882	805575495
Usp18	315879151	-0744294012	036815627	-20216796	004320946	321764868	181349522	211709076	397428215	431243531	351779692
Myl9	404366621	-0743912903	014657929	-5075157	387E-07	220638767	333683121	350832183	471785752	509278074	539981827
Lpxn	442177373	-0743313653	032413096	-2293251	002183356	195357242	384460987	403255382	602552455	513385155	554053015
Phldb2	151658362	-0740957318	016595571	-44647894	801E-06	109170223	114612898	116944061	17948371	1971399	192599381
Icam2	910019733	-0738933358	021131591	-34968183	000047084	643529737	739906051	655289996	117946438	109864423	1143284
Aoc3	580480102	-073861564	026553043	-2781661	000540815	436680893	377207006	493987843	782036165	698203811	694764892
Zfp968-ps	688309493	-0737747286	031726369	-23253442	002005356	344748073	493270701	715778304	551271395	811148546	121363994
Ecscr	198617562	-0737327542	017199718	-42868582	181E-05	108021063	165390764	171383537	255123273	240264253	25152248
Tnnt2	947216276	-0734778683	02200437	-33392397	000084008	689496147	696382165	756103842	987160405	112944734	142470775
Acta2	514732232	-0733958197	011205541	-65499576	576E-11	333256471	383010191	442572782	632039064	651999147	645515735
H2-M3	389215234	-0732596084	033704684	-21735735	002973718	218340446	355445064	292360152	602552455	46204664	404546646
Gm8251	624887799	-0732436163	02607826	-28086083	000497561	448172495	48601672	473825074	807676695	636597593	897038215
Mgp	1221531	-0728085024	011034479	-65982727	416E-11	722821794	99452078	103737447	156150828	156890504	144405564
Pqlc3	312241383	-0726985729	036757289	-19778002	004795125	310273266	188603503	211709076	397428215	431243531	334190707
Lrrc2	167223485	-0725661767	006771286	-1071675	849E-27	121351322	127815143	128840095	206406266	205046031	213882053
Clec14a	217527653	-0722804384	014207545	-50874687	363E-07	167777396	157411385	168359122	253841247	283388606	27438816
Adgrf5	350335098	-0718603116	011580775	-62051384	546E-10	250516933	267671895	276229937	425632798	42816322	453795803

Areg	365943766	-0714822193	033144627	-21566759	003103092	310273266	290159236	231871845	41024848	513385155	439724615
Tmem45a	324710962	-0714025678	035735039	-19981108	004570465	252815254	203111465	282278768	48717007	379905015	3429852
Samd9l	167477952	-0712351721	016829109	-42328547	231E-05	116065185	11751449	148196353	192303975	207407603	223380104
Serpine1	198631719	-0710495605	015537806	-45726893	482E-06	132153428	169743153	148196353	257687326	228969779	255040277
Anxa2	137709904	-071005993	007941027	-89416637	383E-19	946908042	108084315	110491975	166791648	168903716	177296965
Bpifb6	823103786	-0709360295	022424822	-31632818	000156001	666512942	565810509	645208612	111536305	934360983	101136661
Ifit3	229998563	-0708626406	013869974	-5109068	324E-07	170075716	177722532	17642423	267943538	284415376	303409984
Tlr2	589388763	-0708290439	026770435	-26457936	000814995	367731278	420730892	554476151	705114575	749542327	738737353
Fxyd5	166527042	-0705970229	018801111	-37549389	000017338	942311401	14435422	140131245	187175869	194059589	239210191
Col3a1	259145984	-0703933078	013965657	-5040458	464E-07	216042126	171919347	205660245	311532439	341914514	30780723
Gng11	362719075	-0702697642	012410844	-56619652	150E-08	263157696	29596242	269172968	392300109	476421424	47929983
Cfi	785336621	-0702610048	023181056	-30309665	000243772	56308852	674620223	544394766	108972252	934360983	905832707
Gbp2	127360336	-070237349	017862339	-39321472	842E-05	976786208	993795382	937568764	15384318	161202939	158300861
Psmb8	770618602	-0701771312	026521805	-26460164	000814459	402206086	616588376	735941073	101280093	739274624	111690052
Agpat2	42190765	-0700839314	031923146	-21953955	002813524	229832049	348191083	383092613	499990335	472314343	598025476
Tmem252	831600913	-0699625193	024898608	-28098968	000495574	425189291	718144108	746022457	112818332	872754764	109931154
Hpgds	42493075	-0696819959	031140417	-22376706	002524254	287290061	348191083	33268569	525630865	616062187	439724615
Scube1	98931116	-0694846109	021327685	-32579537	000112219	850378581	674620223	746022457	12820265	134506911	103775009
Btc	474561144	-0694658469	010898698	-63737746	184E-10	337853112	353994267	396198413	547425315	625303119	586592636
Cdk1	126985698	-0693862482	018940262	-3663426	000024886	930819798	855969745	112911507	166663445	142721073	160939209
Cd248	887853009	-0693702668	021963489	-31584357	000158618	574580122	783429936	665371381	114100358	103703801	112569501
Clec1a	439733973	-0690746433	032138753	-21492634	003161353	218340446	391714968	393173998	53845113	472314343	624408953
Ifitm3	338311768	-0689436719	012855834	-53628316	819E-08	224086248	260417914	292360152	387172003	426109679	439724615
Itgb2	876243351	-0689342491	021974291	-31370408	000170662	574580122	761667993	665371381	107690226	114998275	10289556
Fhad1	182071926	-0689059801	017847389	-38608437	0000113	170075716	111711306	138114968	237174902	239237482	196117178
Col5a2	179958996	-0688936298	015534354	-44349207	921E-06	131004268	145079618	13710683	20512424	23102332	230415698
H2-Q2	117025371	-0688419782	018820993	-36577229	000025447	919328196	863223726	90732461	15384318	13245337	146868021
Cd109	108158662	-0683681622	022096384	-30940882	000197419	106871903	65285828	786347995	123074544	151962006	123122892
Xlr	350459882	-0681526395	033283445	-20476438	004059491	252815254	290159236	262115998	448709275	400440421	448519107
Cryzl2	170209466	-0680940186	015803979	-43086628	164E-05	128705947	13274785	131057999	194868028	222809157	211067815
Prtg	535753943	-0680233229	010418079	-65293536	661E-11	451619976	408399124	376035644	683320124	623249579	671899212
Itga1	214950052	-067779202	014587836	-4646282	338E-06	185014799	154509793	158277738	251277194	274147673	266473117
Gja1	610215748	-0676675831	009578056	-70648558	161E-12	465409899	438140446	507093643	752549555	750569097	747531845

Skap2	1286788	-0674583645	019527553	-34545222	000055127	804412171	116789092	987975687	156407233	143747844	175889846
Nid1	867799873	-0674094782	008499892	-79306275	218E-15	615949891	686226592	701664365	106408199	108529622	105358018
Anxa4	186300455	-0672525333	015937266	-42198288	244E-05	126407627	138551035	165334707	241020982	23102332	215465061
Cd53	105709467	-0672048588	019747183	-34032631	000066586	815903774	841461783	786347995	134612782	118078586	13719408
Arpc1b	421411021	-067031926	011028663	-60779737	122E-09	339002272	316998965	31957989	537169103	539054413	476661483
Bst2	826132433	-06691755	023034991	-29050391	000367207	655021339	747160032	504069228	10256212	995967202	10289556
Sycp2	449103563	-0667654417	029928729	-22308145	002569342	321764868	384460987	33268569	53845113	492849749	624408953
Ednra	20076064	-066726112	015448005	-43193998	156E-05	134451749	166116162	164326568	22691869	245398104	267352566
Trim34a	818084159	-0666788148	027501949	-24245123	001532896	356239676	848715764	675452765	103844146	872754764	111690052
Atf3	403617158	-0664456117	011627668	-57144399	110E-08	286140901	351092675	296392706	506400467	491822979	489853221
Lin7b	13985571	-0663798826	018067463	-36740013	000023878	976786208	106633519	120976615	152561153	170443872	190840483
Erg	548164279	-0662116005	027092612	-24438988	00145295	436680893	471508758	362929844	64101325	708471515	668381415
Arl6	892277386	-0661925758	007001606	-94539134	326E-21	636290027	719667444	717189697	105318477	104227454	118505784
C4b	493143424	-0660626404	010499834	-62917796	314E-10	413697688	358346656	376035644	612808667	62632989	571641999
Colec12	603951231	-0658987584	025617627	-2572399	001009964	436680893	471508758	493987843	79485643	687936108	738737353
Gpr37l1	624641057	-0657673761	026458147	-24857136	00129292	367731278	48601672	594801689	79485643	800880842	703559384
Piwil4	112776986	-0657072613	020028288	-32807227	000103541	769937364	100830334	836754918	12820265	155042317	131917384
Hmgb1-ps4	761470123	-0655667131	02335377	-2807543	00049921	60905493	623842357	544394766	807676695	954896389	10289556
E2f6	146398014	-0655213185	007337589	-89295437	428E-19	110089551	111203527	119867662	182816979	170957257	183453109
Blvrb	171174048	-0653864321	018268879	-35791158	000034476	98827781	161763774	136098692	20512424	223835928	201393874
Fcer1g	116028194	-0653835595	02663093	-24551737	001408165	448172495	121866879	101821984	151279127	124239208	152144717
Robo4	137443673	-0652563747	017347809	-37616494	00001688	102275262	103731927	114927784	162817365	169417101	1714926
Cd84	650676162	-0650080342	025769648	-2522659	001164713	482647303	507778662	534313382	61537272	89329017	870654738
Zfp97	779608588	-0649969729	009895712	-65681957	509E-11	547000276	617313774	656298135	912802868	899450792	104478569
Hvcn1	390319916	-0649919719	032824525	-19799821	004770554	367731278	282905255	262115998	56409166	390172718	474902584
Map3k8	7760728	-06498298	02412628	-26934522	000707163	60905493	594826433	604883073	114100358	739274624	967394153
Irgm1	137344167	-0648705349	017800774	-36442536	000026817	106871903	110985908	102830122	174355604	147854925	181166541
Tubb6	407831265	-0648704812	010987877	-5903823	355E-09	302229144	332957723	315547337	517938706	505170993	473143686
Muc2	523820764	-0644743181	01082988	-59533733	263E-09	37462624	443218232	404263521	680756071	656106229	583954289
S100a6	766576971	-064380342	010817893	-59512829	266E-09	502183027	663739251	625045843	916648947	101239553	87944923
Higd1b	944537972	-0641057071	027114793	-23642337	001806741	448172495	826953821	937568764	84613749	113971505	146868021
Anxa3	298522411	-0640697009	012652868	-50636506	411E-07	206848844	244459156	246993922	369223632	360396379	363212532
Abracl	126211091	-06400455	019915076	-32138742	000130957	838886979	928509554	119968476	134612782	150935236	175010397

Angptl4	101967433	-0639776026	021971438	-29118532	000359291	655021339	739906051	998057071	112818332	142721073	116966748
Kdelr3	198791711	-0636232156	016442798	-38693666	000010912	121810986	161763774	18247306	229482743	253612267	243607437
Snhg1	318089326	-0635674587	032094399	-19806402	004763163	150539992	309744984	286311321	342301075	392226259	427412326
Ugt1a6a	460557625	-0633777096	03129939	-20248864	004287903	344748073	297413216	443580921	64101325	420975827	615614461
Arhgdib	33990811	-0632975923	013054043	-48488881	124E-06	228682889	286532245	282278768	433324957	370664082	437965716
Il1r1	224213721	-062870352	015271651	-41168014	384E-05	205699684	158862181	165334707	289737989	243344564	282303203
Fignl1	724946172	-0628659763	024167327	-26012797	000928767	540105315	587572452	584720304	71793484	934360983	984983137
Clec7a	645161236	-062511279	025825474	-24205278	001549799	425189291	478762739	614964458	84613749	811148546	694764892
LOC10816736	117435178	-0624496544	019544137	-31953139	000139679	746954159	105182723	957731533	147433047	141694303	139832428
Tagln2	713621491	-0624128441	00864332	-72209344	516E-13	576878443	549126353	559516843	874342073	875835075	846030159
S100a4	634245446	-062374776	026266133	-23747225	001756215	436680893	500524681	564557535	64101325	739274624	923421691
Slc11a1	495576346	-0622226343	028689693	-21688149	003009674	367731278	442492834	352848459	66665378	616062187	527669538
Tpm4	224899728	-0621412778	006283733	-98892303	464E-23	171339792	180333965	1798519	265892296	27425035	277730067
Rnf128	111448271	-062109256	020109863	-30884973	000201172	953803003	935763535	746022457	134612782	120132126	150385818
Gm41263	41545156	-0620640788	011403191	-5442694	525E-08	287290061	333683121	359905429	506400467	504144223	501286061
Gipr	313709824	-0620374197	012098403	-51277364	293E-07	242472812	259692516	238928814	38076187	365530231	394872704
Nmi	952982695	-0618224103	021169151	-2920401	000349581	689496147	81244586	746022457	112818332	130399829	103775009
Lyn	288857351	-0615095896	014071457	-43712311	124E-05	260859376	192955892	232879983	375633764	323432648	347382446
Agtpbp1	122947473	-0610909562	004861953	-12565106	328E-36	963685781	965432317	989991963	145920256	150575866	149277712
Mcub	512065559	-0610113597	028942258	-21080373	003502776	310273266	398968949	504069228	653833515	554455968	65079243
St3gal6	851674368	-0610039362	024386538	-25015415	001236539	482647303	797937898	735941073	858957755	104730572	118725646
Hebp1	927718862	-0609593903	023379377	-26074001	000912327	60905493	681874204	90732461	134612782	913825577	110810603
Gm3604	841027281	-0609233699	024129324	-25248685	001157416	574580122	805191879	614964458	79485643	107810883	117846197
Ddx4	466671052	-0608848769	030988627	-19647491	004944328	298781664	40622293	393173998	71793484	544188265	439724615
Tlr4	630436543	-0607804777	025732096	-23620492	001817423	413697688	544048567	534313382	74357537	852219358	694764892
Glipr2	129682922	-0607009821	018169978	-33407295	000083559	113766864	97928742	977894302	1499971	148881695	169733701
Ptprb	339008992	-0606660932	011901653	-50972831	345E-07	278096779	271298885	257075306	394864162	440484463	392234357
Gm52861	816038104	-0604990817	022734325	-26611338	00077878	632038135	747160032	554476151	10256212	934360983	100257212
Lpar6	281767372	-0604301486	012651607	-477648	178E-06	210296325	235754379	223806737	337172969	332673581	350900243
Tm6sf1	824396955	-060405571	02513229	-24035044	001623877	413697688	739906051	79642938	10256212	104730572	923421691
Shcbp1	521587225	-06026182	027375741	-22012854	002771582	436680893	40622293	403255382	576911925	646865296	659586922
Ets1	304657775	-0601411359	012405606	-48478998	125E-06	23327953	25824172	232879983	374351738	380931785	348261895
Des	490844223	-0601235624	030420814	-19763956	004810999	4711557	413476911	282278768	66665378	636597593	474902584

Gm10030	131997029	-0598853651	018238675	-32834273	000102553	896344991	114612898	109887092	155125206	152988776	169733701
Olfml2b	89640045	-0597649644	021499436	-27798387	000543859	655021339	718144108	766185226	99998067	120132126	103775009
Tnnt1	23289356	-0597030319	014965025	-39895044	662E-05	155136633	192955892	207676522	2564053	284415376	300771637
Gm19378	91663359	-0595698421	020928538	-28463452	000442242	678004544	761667993	746022457	108972252	116025045	106413357
Uhrf1	443750651	-0592508863	029788334	-19890635	00466942	310273266	348191083	403255382	5128106	533920562	554053015
Dnlz	382247491	-0591889117	011950468	-4952853	731E-07	287290061	290884634	335710106	491016149	468207262	420376732
Gm6548	632310167	-059144722	026287966	-22498782	002445668	379222881	558556529	564557535	858957755	729006921	703559384
Mgst1	247037347	-0590227077	01391223	-42425051	221E-05	173523197	195857484	22179046	294866095	293656309	302530535
Cd44	74568158	-0589832184	009889784	-5964055	246E-09	679153705	542597771	567581951	919213	873781535	891761519
Zfp966	191404792	-0589508411	021860454	-26966888	000700327	148241672	163939968	146180076	17563763	336780662	177648744
Col6a3	966143312	-0585663008	020793305	-28165942	000485358	884853388	754414013	68553415	110254279	119105356	117846197
Mis18bp1	459870764	-0585159651	029188563	-20047566	004498907	379222881	333683121	393173998	576911925	513385155	562847507
Grm4	240212221	0584991227	014082638	415398884	327E-05	311422426	27347508	279254352	210252346	190979278	175889846
Ttc34	671763867	0585938231	025161723	232868883	001987556	930819798	689128185	806510765	499990335	585259077	518875046
Dnah11	147510419	0589374923	017313663	340410303	000066382	199953883	163939968	168359122	119228464	120132126	113448951
Mal	671177257	059105699	026043058	226953755	002323566	873361786	906747611	635127227	53845113	616062187	4573136
Adat3	631120993	0595760546	025186973	236535192	001801295	792920569	768921974	715778304	53845113	451778937	518875046
Gal3st3	383218372	0599911745	011341489	52895327	123E-07	475752341	452648408	457694859	289737989	33062004	292856594
Gna14	736066591	0601046154	024709569	243244294	001499735	105722743	855969745	746022457	679474045	523652859	554053015
Wnk4	211624364	0602358526	014829492	406189585	487E-05	279245939	259692516	226831153	169227498	163256479	1714926
Igfn1	667679226	0607745608	009292886	653990188	616E-11	803263011	773274363	84179561	53845113	552402427	496888815
Pou3f3	879279269	0608913773	024890856	244633519	001443168	136750069	754414013	107870815	64101325	739274624	694764892
Rab3b	275354782	0619410979	014898784	415745983	322E-05	267754337	359797452	37200309	221790584	201246981	229536249
Pcdhga8	138624725	0620431304	018135331	34211193	000062364	18731312	160312978	157269599	108972252	965164092	121363994
Wdr46	142319077	0623237048	018863406	330394759	000095334	206848844	14798121	16331843	120510491	114998275	100257212
A930017K11F	86148598	0635494782	021828723	29112779	000359954	111468544	106633519	967812917	61537272	718739218	685970399
H19	432216029	0643575047	014035123	458546056	453E-06	399907765	596277229	582704027	343583102	335753892	335070157
Slc13a5	113687948	0644768589	020910732	30834338	000204627	164329915	1138875	138114968	101280093	800880842	844271261
Glb1l3	29270965	064981978	013020528	499073312	602E-07	319466548	384460987	367970536	223072611	223835928	237451292
Asb2	563115144	0651789709	028248128	230737312	002103403	838886979	565810509	665371381	474349805	359369609	474902584
Prickle4	544472806	0654797987	030605112	213950528	003239477	689496147	819699841	493987843	30768636	410708124	545258523
Gm40078	385453316	0658125184	032499633	202502343	004286496	4711557	457000796	493987843	23076477	308031093	351779692
Cilp2	432155608	0658540958	03041743	216501182	003038678	482647303	500524681	604883073	33332689	328566499	3429852

Rps2-ps10	164563216	0662249698	017413033	380318403	000014285	23557785	192955892	17642423	134612782	13348014	1143284
9630028B13F	707860168	0670563218	024093281	278319589	000538263	919328196	870477707	816592149	551271395	605794483	483697076
Krt1	460774656	0676283449	030029413	225207016	002431784	586071725	500524681	614964458	397428215	287495687	378163169
Adat2	513413952	0685084941	028989675	236320327	001811773	666512942	500524681	735941073	397428215	410708124	369368677
3010001F23F	402225784	0692335573	033080959	209285218	003636235	379222881	478762739	635127227	269225565	308031093	3429852
Perp	814547134	0704213561	024248808	290411621	000368291	781428966	986541401	126017307	602552455	605794483	65079243
Uty	709792782	072517241	009180043	78994443	280E-15	917029875	877731688	858933964	556399501	492849749	555811913
Kdm5d	628155664	0735145129	009241485	795483748	179E-15	788323928	789233121	775258472	499990335	451778937	464349193
Gm13584	446063357	0741906036	033571098	220995464	002710831	60905493	398968949	675452765	243585035	441511234	30780723
Nptxr	378027563	0754149383	014483972	520678565	192E-07	568834321	375030812	481890182	293584068	270040592	278785406
Ppp1ccb	128028161	0757570168	022732959	333247491	000086077	222937087	13274785	128033584	99998067	841951655	100257212
Gm14137	500229569	0761944992	029756227	25606237	001044845	735462557	587572452	564557535	371787685	451778937	290218246
Ntn3	436962072	0768308191	035943586	213753908	003255417	861870183	391714968	403255382	346147155	328566499	290218246
Gm6999	707587245	0771358324	024860905	310269605	000191766	815903774	950271497	917405995	43588901	492849749	633203446
Dock8	235147348	0782374526	014510867	539164551	698E-08	291886702	309744984	288327598	20127816	156069087	163577557
Asprv1	730511627	0782882731	025017374	312935617	00017519	114916024	819699841	806510765	576911925	503117452	527669538
Ung	256829786	0792059761	039414917	200954316	004447956	287290061	362699044	322604306	217944505	174550953	175889846
Col1a2	221450772	0799712588	015669076	510376364	333E-07	313720747	263319506	266148552	174355604	176604493	134555732
Vwa5b1	617730933	0807969136	026156342	308899897	000200832	769937364	696382165	897243226	41024848	492849749	439724615
Ndufa12-ps	875028026	0809769316	022797352	355203233	000038227	999769413	125493869	107870815	64101325	739274624	53646403
Ywhaq-ps3	28680068	0813845589	038068952	213781977	003253137	275798459	384460987	433499536	217944505	215621765	193478831
Pou6f2	269332198	0817199461	03964707	206118502	003928539	413697688	282905255	33268569	243585035	184818656	158300861
Ccdc63	398843261	0818701649	032959394	248397059	001299265	643529737	457000796	433499536	2564053	277227984	325396215
Gm46911	345775368	082235126	038269676	214883259	003164767	620546532	398968949	302441537	33332689	225889468	193478831
Gm4202	367314052	0826808901	036072518	229207428	002190136	632038135	442492834	33268569	33332689	225889468	237451292
Igsf1	510080139	0839876532	02918563	287770571	000400579	689496147	710890127	554476151	448709275	349101906	30780723
Itpka	25865936	0841026848	041840042	201010038	004442057	402206086	275651274	322604306	192303975	112944734	246245784
Gm39876	264753329	0857946531	041884597	204835809	004052492	321764868	420730892	272197383	28204583	13348014	158300861
Gm34640	88356892	0859913893	023626107	363967663	000027298	110319383	107358917	12400103	730755105	441511234	712353876
Gm30273	425632907	08638788	032993965	261829336	000883708	574580122	464254777	614964458	30768636	205354062	386957661
Rbx1-ps	26211546	0879445272	040776462	215674736	003102535	344748073	333683121	342767075	192303975	112944734	246245784
Wdr72	271927627	0886356187	039623661	223693666	002529048	321764868	333683121	403255382	166663445	256692578	149506369
Lcn2	770109971	0896812753	014081441	636875674	191E-10	717075993	114395279	114322901	544861262	511331615	560209159

Impdh2-ps	25354193	0901137936	041694652	216127945	003067376	413697688	355445064	22179046	166663445	205354062	158300861
Tmem150b	473975606	0912081082	029728261	306806062	000215453	51712211	689128185	645208612	35896742	308031093	325396215
Prokr1	211307374	0926612766	044736874	207125057	003833539	310273266	311921178	211709076	10256212	16428325	167095354
Fam107a	139724956	0933844281	017784906	52507687	151E-07	172374037	179898726	198603276	87177802	102677031	976188645
4930526A20	231824858	0954903496	043223096	220924362	00271577	287290061	348191083	282278768	115382385	225889468	131917384
Gm13112	270825396	0966114151	01371811	704261833	189E-12	337853112	372854618	361921706	196150054	196113129	16005976
Chil1	447117208	0971893137	0112926	860646055	754E-18	534359514	597728025	643192335	316660545	292629539	298133289
1700001K19	389338288	0979108269	036455606	268575504	000723661	769937364	420730892	362929844	269225565	266960281	246245784
Gm30266	446222376	0979832033	031615886	309917624	00019406	746954159	478762739	554476151	30768636	308031093	281423754
Gm12405	233134499	1002301994	042615599	235196038	001867476	367731278	304667197	262115998	166663445	112944734	184684338
Gm21596	506518568	1008475332	039520568	255177336	001071762	976786208	565810509	504069228	15384318	266960281	571641999
Il17rb	16853532	1010126808	05113492	197541484	004822109	275798459	261143312	141139384	89741855	102677031	140711877
Olfm4	290414852	1024490425	013835191	740496064	131E-13	333256471	417103901	417369321	180765736	202273751	191719932
LOC1154904	179695811	1039432074	051719478	200974972	004445768	379222881	15958758	191546307	89741855	143747844	1143284
Gm14760	616514545	1057760201	033147674	319105405	000141755	131004268	638350318	554476151	41024848	451778937	334190707
Mid1-ps1	841472043	1079991647	026114112	413566292	354E-05	953803003	137100239	109887092	43588901	749542327	439724615
Scube2	200001337	1081899	047157872	229420656	002177864	310273266	210365446	292360152	15384318	154015547	791504307
Gm31796	180596014	1082280279	049591912	218237255	002908205	321764868	24663535	171383537	7692159	143747844	123122892
Mybphl	276312513	1085046498	039648262	273668109	000620624	321764868	384460987	413336767	243585035	154015547	140711877
Pax7	22496816	109272218	045760467	238791745	001694415	402206086	333683121	181464922	15384318	16428325	1143284
Gm40099	132213408	1112486285	055153444	201707491	00436877	206848844	174095541	161302153	89741855	821416249	791504307
Prr15l	235134616	1122567468	048878471	229665014	002163874	528613713	224873408	211709076	20512424	143747844	967394153
F2rl1	759525242	1124397021	024423708	460371134	415E-06	110319383	102281131	100813846	423068745	410708124	589230984
Gm17250	136691156	115244354	055887288	206208528	003919962	126407627	217619427	22179046	7692159	718739218	105533908
Bdkrb2	21664663	115324628	043744198	263634115	000838054	241323651	311921178	342767075	12820265	143747844	131917384
Gm2000	32221932	1170605647	048743096	240158245	001632433	838886979	203111465	302441537	217944505	13348014	237451292
Cldn10	555271711	1189882002	028724757	41423571	344E-05	861870183	834207802	614964458	397428215	29776339	325396215
Steap4	956563981	123451869	023557495	524044969	160E-07	124109306	127670064	152228907	499990335	451778937	747531845
Gm10039	161831969	1257771802	05701262	220612877	002737499	298781664	145079618	241953229	7692159	16428325	439724615
Myh7	136054877	1283075427	057008185	225068631	002440541	229832049	217619427	131057999	89741855	513385155	967394153
Aqp5	190281865	1287404126	049436395	260416263	00092099	241323651	232127388	342767075	5128106	13348014	140711877
Cited4	275412163	131506228	044010264	298808085	000280735	586071725	282905255	312522921	141022915	215621765	1143284
Gm30483	157453333	131803759	05364692	245687466	001401516	321764868	203111465	151220768	89741855	821416249	967394153

Krt6b	175723821	1338946787	050907203	263017158	000853418	252815254	217619427	282278768	115382385	13348014	527669538
Meltf	132613642	1373037014	057284861	239685841	001653631	160882434	15958758	252034614	89741855	718739218	615614461
Prss27	11957424	138818732	060874115	2280423	002258261	195357242	123317675	201627691	7692159	410708124	791504307
Gm35365	126912034	1389433985	064199728	216423657	00304462	264306856	870477707	201627691	89741855	308031093	87944923
A530076I17R	113159413	1404858278	062309421	225464826	002415542	195357242	108809713	191546307	5128106	616062187	703559384
Traf1	136941926	1423729397	056176215	253439892	001126404	172374037	210365446	211709076	10256212	718739218	527669538
Gm46738	109350648	1457653885	064216552	226990368	002321343	229832049	123317675	131057999	38460795	718739218	615614461
Gm52523	927543675	1493069188	069149329	215919548	0030835	172374037	137825637	100813846	38460795	718739218	351779692
Gm39323	115256177	1519272836	064287555	236324565	001811566	149390832	101555732	262115998	64101325	616062187	527669538
5730409K12F	833567541	1529726132	078040505	196016944	004997599	218340446	725398089	806510765	64101325	205354062	439724615
Amd-ps4	168943657	1534951856	072253544	212439665	003363699	597563327	108809713	504069228	89741855	616062187	105533908
LOC10524691	765150071	15542368	076703711	202628633	004273545	149390832	725398089	120976615	38460795	513385155	263834769
Gm52619	782190956	1598531154	07869848	203120969	004223373	114916024	174095541	604883073	64101325	205354062	351779692
Abcc2	753157873	1695148645	078075786	217115797	002991923	149390832	943017516	100813846	38460795	513385155	175889846
Krt6a	141617026	1747227564	0562032	310876885	000187869	206848844	24663535	201627691	64101325	513385155	791504307
Xlr4b	102194342	178283678	069800537	255418777	001064358	183865639	797937898	211709076	5128106	513385155	351779692
BC055402	78463433	1783694252	025761977	692374752	440E-12	999769413	130571656	133074276	41024848	379905015	281423754
Gm30938	857417183	1835231985	072374118	253575729	001122045	149390832	123317675	131057999	2564053	410708124	439724615
Gm9824	141832683	1835899219	075192189	244160895	001462198	459664098	101555732	11089523	0	821416249	967394153
Krt15	149112185	1836167777	056421725	325436307	000113647	172374037	224873408	302441537	5128106	821416249	615614461
Atp13a4	888659822	1900317196	071650392	265220768	000799673	160882434	130571656	131057999	2564053	410708124	439724615
Vis1	55776736	1995493771	099264497	201027944	004440162	459664098	116063694	100813846	5128106	205354062	0
Krt12	433770265	2065526524	033808673	610945761	100E-09	60905493	768921974	725859688	141022915	16428325	193478831
Actb-ps1	611932538	217833609	100445587	216867277	003010754	137899229	870477707	806510765	0	0	615614461
Gm17803	581816985	2187576092	098146775	22288823	002582174	183865639	435238853	604883073	12820265	308031093	175889846
Gm39786	394789856	2219363526	110612709	200642724	004481068	689496147	65285828	604883073	12820265	205354062	087944923
Ldoc1	814590562	2246399049	081319349	276244102	000573709	218340446	943017516	90732461	38460795	205354062	263834769
Gm31399	416304503	2307043295	109277639	2111176	003475719	689496147	580318471	806510765	12820265	205354062	087944923
Trp63	166463169	2327682552	057133984	407407709	462E-05	356239676	275651274	201627691	64101325	308031093	703559384
Anxa8	432780271	2358793885	109737199	214949344	003159531	103424422	435238853	705696919	12820265	205354062	087944923
Gm10433	356057382	2573919699	125653658	204842401	004051847	804412171	725398089	302441537	12820265	0	175889846
Mb	173816268	2593653187	056920406	455663156	520E-06	287290061	261143312	342767075	7692159	308031093	439724615
Gm14824	368383869	263532882	125609087	209803994	003590162	103424422	580318471	302441537	0	205354062	087944923

Krt5	189996177	2654936836	054427223	487795758	107E-06	356239676	304667197	322604306	64101325	308031093	615614461
Gm10269	266884652	2917415093	141049646	206836045	003860614	128705947	870477707	403255382	7692159	308031093	791504307
Gm32768	279279312	2920367947	148094932	197195671	004861455	459664098	435238853	604883073	0	0	175889846
Gm50287	298840284	2990780431	143176228	208888059	003671847	574580122	725398089	302441537	0	102677031	087944923
Rps18-ps3	314581212	3089103811	153974669	200624156	004483048	126407627	145079618	302441537	0	0	175889846
Gm17875	367560087	32409151	148627753	21805585	002921609	919328196	072539809	100813846	0	205354062	0
Gm39425	566337374	3321105669	113931915	291499152	000355698	804412171	137825637	90732461	0	308031093	0
Gm3985	383598275	3354750572	151795056	221005259	002710151	0	108809713	100813846	0	205354062	0
LOC1008619	425694404	3492122523	150618371	231852363	002042088	114916024	217619427	201627691	0	205354062	0
Gm9522	318639544	3512838058	048859525	71896689	649E-13	60905493	638350318	514150612	2564053	718739218	527669538
Gm36736	242792251	3585133353	161975924	221337422	002687186	574580122	290159236	504069228	0	0	087944923
Cldn4	245865842	3618468464	175696315	205950162	003944621	574580122	797937898	0	0	102677031	0
Alb	16196624	38348482	171248612	223934556	002513344	873361786	145079618	201627691	2564053	205354062	175889846
Dhrs9	137387063	3859123057	195990883	196903193	004894942	114916024	507778662	201627691	0	0	0
Gm16073	146485203	3908530397	189216065	206564406	00388621	459664098	217619427	201627691	0	0	0
Odf3l1	158260245	4004899829	19053699	210190148	003556191	574580122	072539809	302441537	0	0	0
Gm15191	156236421	4015697865	189568056	211834101	00341462	114916024	217619427	604883073	0	0	0
Gm7511	162960971	4042680966	195547107	206736936	003869936	804412171	072539809	100813846	0	0	0
Gm10536	172700576	4133290868	184590223	223917107	002514479	689496147	145079618	201627691	0	0	0
Gm17980	189502884	4268116607	179025964	238407688	001712203	689496147	145079618	302441537	0	0	0
Mgat4e	18949127	4272484648	195492723	218549549	002885254	919328196	217619427	0	0	0	0
Krt17	19420361	4300183006	184655056	232876538	00198715	919328196	145079618	100813846	0	0	0
Gsx1	192494712	4327276068	174005659	248685938	001288763	344748073	507778662	302441537	0	0	0
Acox2	213694433	445336453	17081964	26070565	000913243	459664098	217619427	604883073	0	0	0
1700047F07R	264078129	4755769226	163403511	291044495	000360915	919328196	362699044	302441537	0	0	0
