# Supplementary material for: Zeb2 Controls Retinal Physiological and Pathological Angiogenesis by Regulating Astrocyte Proliferation and Differentiation
Source: Cell Prolif. 2026 May 26:e70236. Online ahead of print. doi: 10.1111/cpr.70236 (PMC13325830; doi:10.1111/cpr.70236)
Supplement: Supplementary file 3 — Table S3: Vascular markers and primary antibodies used in Western blotting, immunostaining, ChIP or CUT&Tag Table S4: Secondary antibodies used in Western blotting or immunostaining Table S5: Gene‐specific primer sequences used for qRT‐PCR analysis Table S6: Gene‐specific primer sequences used for ChIP assay [file CPR-9999-e70236-s004.docx]

**Supplementary Tables S3-S6**

**Table S3. Vascular markers and primary antibodies used in Western blotting, immunostaining, ChIP or CUT&Tag**

| Name | Supplier | Cat. No | Host | Dilution |
| --- | --- | --- | --- | --- |
| IB4-488 | Invitrogen | I21411 |  | 1:500 |
| IB4-568 | Invitrogen | I21412 |  | 1:500 |
| Zeb2 | Santa Cruz | sc-48789 | Rb | 1:200 |
| Pax2 | R&D Systems | AF3364 | Gt | 1:500 |
| GFAP | Dako | Z0334 | Rb | 1:500 |
| ERG-647 | Abcam | ab196149 | Rb | 1:500 |
| GAPDH | Cell Signaling Technology | 5174 | Rb | 1:800 |
| C3 | Abcam | ab321966 | Rb | 1:800 |
| Serpina3n | R&D Systems | AF4709 | Gt | 1:800 |
| S100a10 | Proteintech | 11250-1-AP | Rb | 1:800 |
| Nlrp3 | Abcam | ab214185 | Rb | 1:800 |
| Gsdmd | Abcam | ab219800 | Rb | 1:800 |
| Casp1 | Abcam | ab179515 | Rb | 1:800 |
| TNF-a | Abcam | ab215188 | Rb | 1:800 |
| HIF-2a | Novus Biologicals | NB100-122 | Rb | 1:800 |
| VEGFA | Abcam | ab52917 | Rb | 1:800 |
| S100B | Abcam | ab52642 | Rb | 1:1000 |
| SOX9 | Millipore | AB5535 | Rb | 1:1000 |
| IgG | Cell Signaling Technology | 5415 | Ms | 1:250 |
| H3K27me3 | Active Motif | 39157 | Rb | 1:250 |
| IgG | Cell Signaling Technology | 2729 | Rb | 1:250 |

**Table S4. Secondary antibodies used in Western blotting or immunostaining**

| Name | Supplier | Dilution |
| --- | --- | --- |
| Anti-rabbit IgG, HRP-linked Antibody | Cell Signaling Technology | 1:4000 |
| Anti-mouse IgG, HRP-linked Antibody | Cell Signaling Technology | 1:4000 |
| Anti-goat IgG, HRP-linked Antibody | Cell Signaling Technology | 1:4000 |
| Donkey anti-Goat Alexa 488 IgG | Invitrogen | 1:1000 |
| Donkey anti-Rabbit Alexa 546 IgG | Invitrogen | 1:1000 |
| Donkey anti-Rabbit Alexa 488 IgG | Invitrogen | 1:1000 |
| Donkey anti-Rabbit Alexa 405 IgG | Invitrogen | 1:1000 |

**Table S5. Gene-specific primer sequences used for qRT-PCR analysis**

| Gene | Forward Primer | Reverse Primer |
| --- | --- | --- |
| *Gapdh* | TGACCTCAACTACATGGTCTACA | CTTCCCATTCTCGGCCTTG |
| *C3* | ACTGTGGACAACAACCTACTGC | GCATGTTCGTAAAAGGCTCGG |
| *Serpina3n* | AGGCTGACCTGTCTGCAATC | GTCAGAGGGTACAGTTTCGCA |
| *H2-D1* | ACCTGCAGTTCGCCTATGAAG | TAATGCTCTGCAGCACCACTCT |
| *Gbp2* | CCTGACCAGAGTGGGGTAGA | CAGTCGCGGCTCATTAAAGC |
| *Serping1* | TAGAGCCTTCTCAGATCCCGA | ACTCGTTGGCTACTTTACCCA |
| *S100a10* | ATGCCATCCCAAATGGAGCA | GAACTCCCGTTCCATGAGCA |
| *Tgm1* | TCTGGGCTCGTTGTTGTGG | AACCAGCATTCCCTCTCGGA |
| *Ptgs2* | TGAGCAACTATTCCAAACCAGC | GCACGTAGTCTTCGATCACTATC |
| *Hspb1* | ATCCCCTGAGGGCACACTTA | GGAATGGTGATCTCCGCTGAC |
| *Ccl2* | TTAAAAACCTGGATCGGAACCAA | GCATTAGCTTCAGATTTACGGGT |
| *Ccl6* | GCTGGCCTCATACAAGAAATGG | GCTTAGGCACCTCTGAACTCTC |
| *Ccl11* | TCTATTCCTGCTGCTCACGG | GCTTTCAGGGTGCATCTGTTG |
| *Ccr1* | CTCATGCAGCATAGGAGGCTT | ACATGGCATCACCAAAAATCCA |
| *Ccr2* | ATCCACGGCATACTATCAACATC | CAAGGCTCACCATCATCGTAG |
| *Cxcl10* | CCAAGTGCTGCCGTCATTTTC | GGCTCGCAGGGATGATTTCAA |
| *Il-1b* | GCAACTGTTCCTGAACTCAACT | ATCTTTTGGGGTCCGTCAACT |
| *Il-2* | GATGAACTTGGACCTCTGCG | GAAAGTCCACCACAGTTGCTG |
| *Il-5* | CTCTGTTGACAAGCAATGAGACG | TCTTCAGTATGTCTAGCCCCTG |
| *Il-6* | TAGTCCTTCCTACCCCAATTTCC | TTGGTCCTTAGCCACTCCTTC |
| *Il-10* | GCTCTTACTGACTGGCATGAG | CGCAGCTCTAGGAGCATGTG |
| *Cldn5* | GCAAGGTGTATGAATCTGTGCT | GTCAAGGTAACAAAGAGTGCCA |
| *Sox18* | CCTGTCACCAACGTCTCGC | GCAACTCGTCGGCAGTTTG |
| *Aplnr* | GGTTACAACTACTATGGGGCTGA | AGCTGAGCGTCTCTTTTCGC |
| *Icam1* | TGCCTCTGAAGCTCGGATATAC | TCTGTCGAACTCCTCAGTCAC |
| *Icam2* | GCAGGACAACCAAATGGTCAT | AGAACAGCAGTATTGACACCAC |
| *Pecam1* | GGTGCATGGCGTATCCAAG | TGGAGGTCTTATCTATCCTTCGC |
| *Tgfbi* | CAGCACGGCCCCAATGTAT | GGGACCTTTTCATATCCAGGACA |
| *Cd93* | ATCTCAACTGGTTTGTTCCTGC | ACTCTTCACGGTGGCAAGATT |
| *Eng* | AGGGGTGAGGTGACGTTTAC | GTGCCATTTTGCTTGGATGC |
| *Ptprb* | TCAAGGCAGGACAGTACCC | TGTATTTCTCCCATTCGCCTAGA |
| *Ets1* | TCCTATCAGCTCGGAAGAACTC | TCTTGCTTGATGGCAAAGTAGTC |
| *Anxa3* | ATGGCCTCTATCTGGGTTGGA | CAAGTCCTCTGATCGCTTTCC |
| *Clec14a* | CTTCACCACGCTACCTTCAAG | AAACCCCTTAAAGGCTCTTTCTC |
| *Tspan12* | CAGAGAAGATTCCGTGAAGTGTT | TCAGGTAGTCCCTCATCCAAG |
| *Angpt1* | CACATAGGGTGCAGCAACCA | CGTCGTGTTCTGGAAGAATGA |
| *Angpt2* | CAGCCACGGTCAACAACTC | CTTCTTTACGGATAGCAACCGAG |
| *Angpt4* | TGAAGCACAATCTGCACGCT | GGGCTACAATCCGTACCAGG |
| *Angptl2* | GGGAGACCTATAAGCAAGGGT | CATGGTTACCAGCAGTTTGTAGT |
| *Angptl4* | CTCCGTGGGGACCTTAACTG | GCCGTGGGATAGAGTGGAAG |
| *Six3* | GCTCCCGGCTTCTCTTACC | CGGCGAAGTTTGGCAACAAG |
| *Col15a1* | CTTTGACGGTCGGGATGTGA | GTCCACAAGACGGACACCAT |
| *Nkd1* | CAGCTTGCTGCATACCATCTAT | GTTGAAAAGGACGCTCCTCTTA |
| *Mal* | AGTGGCTTCTCGGTCTTCAC | GGTAGGCTGCATCAAACTCAC |
| *Gpx3* | CCTTTTAAGCAGTATGCAGGCA | CAAGCCAAATGGCCCAAGTT |
| *Esm1* | CTGGAGCGCCAAATATGCG | TGAGACTGTACGGTAGCAGGT |
| *Prss23* | GGTGAGTCCCTACACCGTTC | GGCGTCGAAGTCTGCCTTAG |
| *Mcam* | AAGCTGGTCACTTTAACCACC | ATTCACTCTTACGAGTCGGGG |
| *Fzr1* | GTTTCAGAGATGCGGAGAACC | CAGGCCGTCTTTGCCATTG |
| *Apln* | GGACCGAGTTGCAGCATGAA | AGGTAGCGCATGCTTCCTTC |
| *Serpine1* | TCTGGGAAAGGGTTCACTTTACC | GACACGCCATAGGGAGAGAAG |
| *Nid2* | TGGATTACCCAATGGATTGACCT | GTGGTTTTGGATGACACGTCG |
| *Plxnd1* | TCGCTGCCAATCCCTAATAAGA | TGACCTGGTTTGGAACTGTTG |
| *Rhoj* | GTAAACCCAGCCTCTTACCAC | GATGAGCACATAAGGCACATGA |
| *Nfe2l2* | TCTTGGAGTAAGTCGAGAAGTGT | GTTGAAACTGAGCGAAAAAGGC |
| *Hmox1* | AAGCCGAGAATGCTGAGTTCA | GCCGTGTAGATATGGTACAAGGA |
| *Igfbp3* | CCAGGAAACATCAGTGAGTCC | GGATGGAACTTGGAATCGGTCA |
| *Tie1* | TTTTCTTGGCCTCTCATGTTGG | CGCACGATGCGATCATCCTT |
| *Gdnf* | CCAGTGACTCCAATATGCCTG | CTCTGCGACCTTTCCCTCTG |
| *Tgm2* | CTAGAGGCTTCTACTGGCTACC | GCGTAAGGACATATTCCCGTC |
| *Cdh5* | GTCGATGCTAACACAGGGAATG | AATACCTGGTGCGAAAACACA |
| *Wnt4* | TGCGAGGTAAAGACGTGCTG | CTTGAACTGTGCATTCCGAGG |
| *Ntrk1* | GCCTAACCATCGTGAAGAGTG | CCAACGCATTGGAGGACAGAT |
| *C3ar1* | TCGATGCTGACACCAATTCAA | TCCCAATAGACAAGTGAGACCAA |
| *Cd34* | AAGGCTGGGTGAAGACCCTTA | TGAATGGCCGTTTCTGGAAGT |
| *Cldn1* | GGGGACAACATCGTGACCG | AGGAGTCGAAGACTTTGCACT |
| *Ednra* | ATGAGTATCTTTTGCCTTGCGG | GTCTTCCATGTGGCTGCTTAG |
| *Vegfa* | GCACATAGAGAGAATGAGCTTCC | CTCCGCTCTGAACAAGGCT |
| *Nkx6-1* | CTGCACAGTATGGCCGAGATG | CCGGGTTATGTGAGCCCAA |
| *Fgf17* | GCTGCCTAACCTTACCCTGTG | CCTGGTCCCTCACGTACTG |
| *Fgf23* | ATGCTAGGGACCTGCCTTAGA | AGCCAAGCAATGGGGAAGTG |
| *Cyp1b1* | CAGTCTGGCGTTCGGTCAC | GCTGCGTTGGATCGAGGAA |
| *Cdkn1b* | TCAAACGTGAGAGTGTCTAACG | CCGGGCCGAAGAGATTTCTG |
| *Hif1a* | ACCTTCATCGGAAACTCCAAAG | ACTGTTAGGCTCAGGTGAACT |
| *Epas1* | CTGAGGAAGGAGAAATCCCGT | TGTGTCCGAAGGAAGCTGATG |
| *Fgf2* | GCGACCCACACGTCAAACTA | TCCCTTGATAGACACAACTCCTC |
| *Slc2a1* | AGCAGCAAGACCGATGAACA | TAGCCGAACTGCAGTGATCC |
| *Ldha* | TGTCTCCAGCAAAGACTACTGT | GACTGTACTTGACAATGTTGGGA |

**Table S6. Gene-specific primer sequences used for ChIP assay**

| Gene | Forward Primer | Reverse Primer |
| --- | --- | --- |
| *Vegfa* site 1 | TGGCTTTCCTTTCTGACTCC | AGTTAGTAAATGTTTGCTGGTCC |
| *Vegfa* site 2 | TCGTGAACTTGGGCGAGCC | CACCTCACAAACACACTACACA |
| *Vegfa* site 3 | ACCCGGAGAAACTCCCATACCTCT | CCAGGAAAGGTCCGATGAGTCTTC |
| *Vegfa* site 4 | AGGGCAAGATTGGAACGGGACT | ACTCTGGACGGCGTCAATTCTGA |
