# Supplementary material for: Zeb2 Controls Retinal Physiological and Pathological Angiogenesis by Regulating Astrocyte Proliferation and Differentiation
Source: Cell Prolif. 2026 May 26:e70236. Online ahead of print. doi: 10.1111/cpr.70236 (PMC13325830; doi:10.1111/cpr.70236)
Supplement: Supplementary file 4 — Figure S1: Zeb2 is transiently expressed in astrocyte progenitor cells in developing mouse retinas. (A–D) Double‐immunofluorescence staining of wild‐type whole‐mount retinas for Pax2 (green) and Zeb2 (red) with DAPI counterlabeling (blue) at developmental stages E15.5, E17.5, and P0. The image shown in (B) is from the central region of a representative E17.5 retina and that in (C) is from the intermediate to peripheral region. Note that the Zeb2‐immunoreactive cells in (D) are retinal ganglion cells and amacrine cells located within the retinal ganglion cell layer. Abbreviation: ONH, optic nerve head. The insets in (B, C) show corresponding outlined regions at a higher magnification. Scale bar, 100 μm. Figure S2: The retinal and eyeball sizes of control and Zeb2CKO mice. (A) PCR analysis of genomic DNA from Zeb2fl/fl, Zeb2+/+ and Zeb2+/fl mice. The wild‐type and floxed (fl) alleles yield a product of 197 bp and 339 bp, respectively. (B) Retinal morphology of P16 control and Zeb2CKO mice. (C) The morphology of eyeballs (upper) and retinas (lower) of P25 control and Zeb2CKO animals. Scale bar: B, C, 1 mm. Figure S3: The astrocytes and vasculature are normal in Zeb2CKO retinas after P12. (A) P12 control and Zeb2CKO flat‐mount retinas were triple‐immunostained for IB4, Pax2 and GFAP. Shown are representative confocal images in the central, intermediate and peripheral regions. (B‐D) Quantification of Pax2+ cells (B), GFAP+ area (C) and GFAP+/Pax2+ area ratio (D) in P12 control and Zeb2CKO retinas in the central, intermediate and peripheral regions. Data are presented as mean ± SEM (n = 6). (E) IB4 immunofluorescence (vitreal surface vasculature, blue; IPL vasculature, green; OPL vasculature, red) images of P16 control and Zeb2CKO flat‐mount retinas in the central, intermediate and peripheral regions. (F‐H) Quantification of IB4+ vascular area of P16 control and Zeb2CKO retinas in the central (F), intermediate (G) and peripheral (H) regions. Data are presented as mean ± S [file CPR-9999-e70236-s003.docx]

**Supplementary Figures S1-S11**


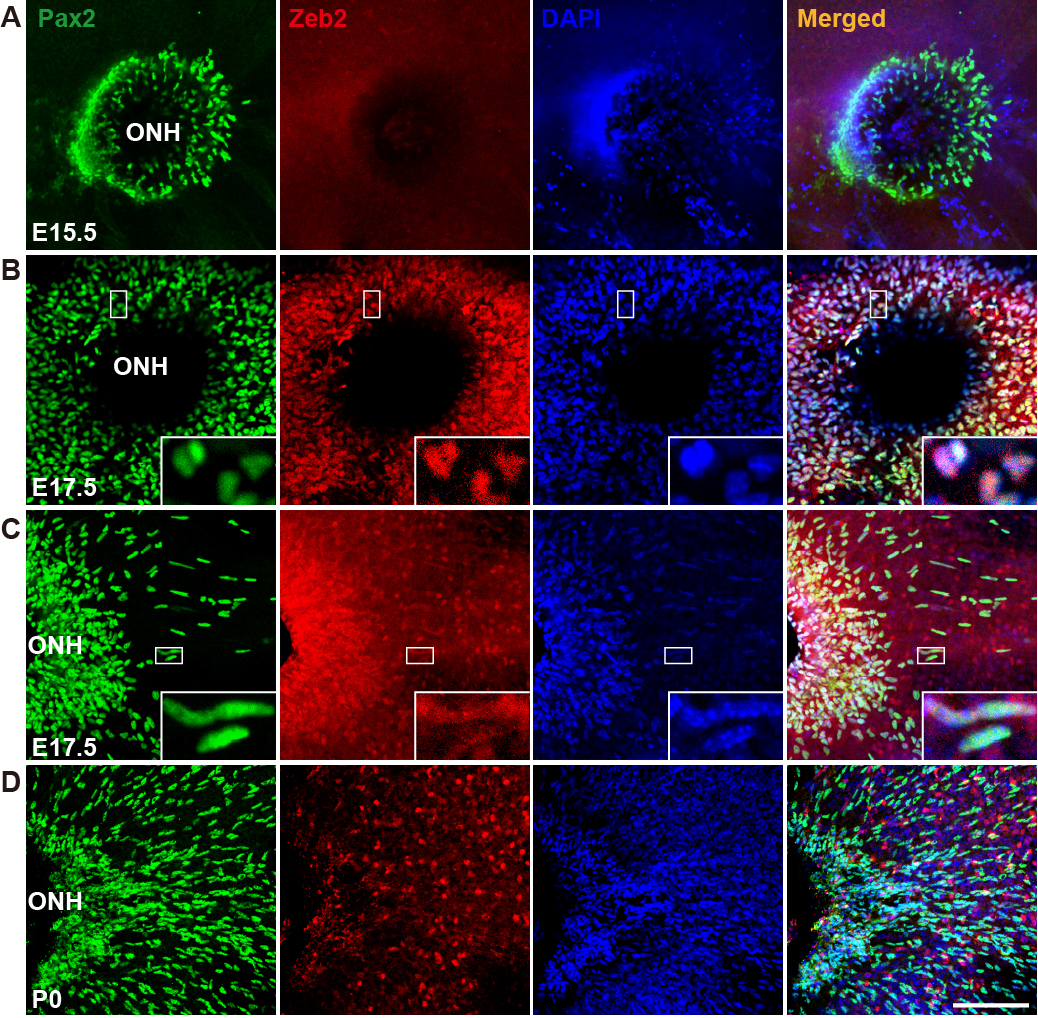


**Figure S1. Zeb2 is transiently expressed in astrocyte progenitor cells in developing mouse retinas.** **(A-D)** Double-immunofluorescence staining of wild-type whole-mount retinas for Pax2 (green) and Zeb2 (red) with DAPI counterlabeling (blue) at developmental stages E15.5, E17.5 and P0. The image shown in (B) is from the central region of a representative E17.5 retina and that in (C) is from the intermediate to peripheral region. Note that the Zeb2-immunoreactive cells in (D) are retinal ganglion cells and amacrine cells located within the retinal ganglion cell layer. Abbreviation: ONH, optic nerve head. The insets in (B,C) show corresponding outlined regions at a higher magnification. Scale bar, 100 μm.


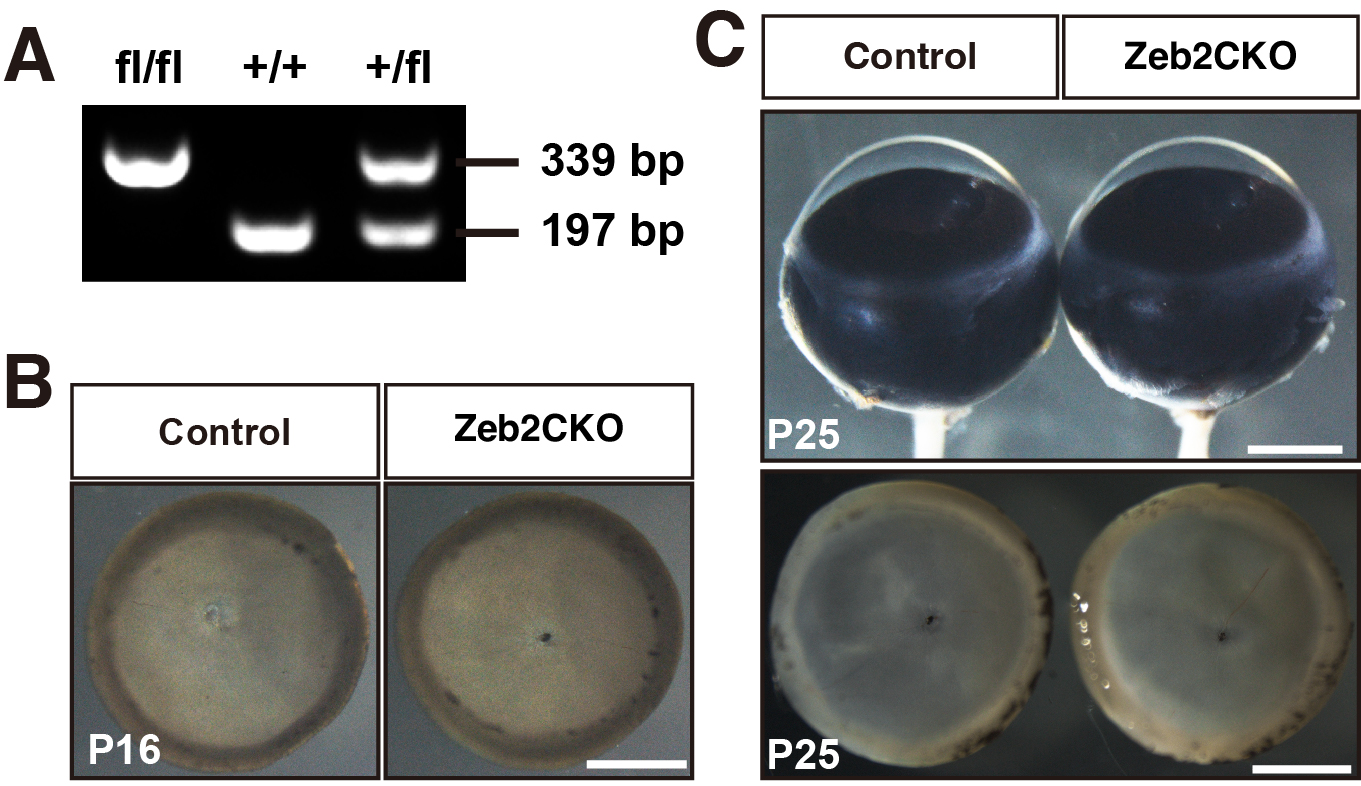


**Figure S2. The retinal and eyeball sizes of control and Zeb2CKO mice.** **(A)** PCR analysis of genomic DNA from Zeb2^fl/fl^, Zeb2^+/+^ and Zeb2^+/fl^ mice. The wild-type and floxed (fl) alleles yield a product of 197 bp and 339 bp, respectively. **(B)** Retinal morphology of P16 control and Zeb2CKO mice. **(C)** The morphology of eyeballs (upper) and retinas (lower) of P25 control and Zeb2CKO animals. Scale bar: B,C, 1 mm.


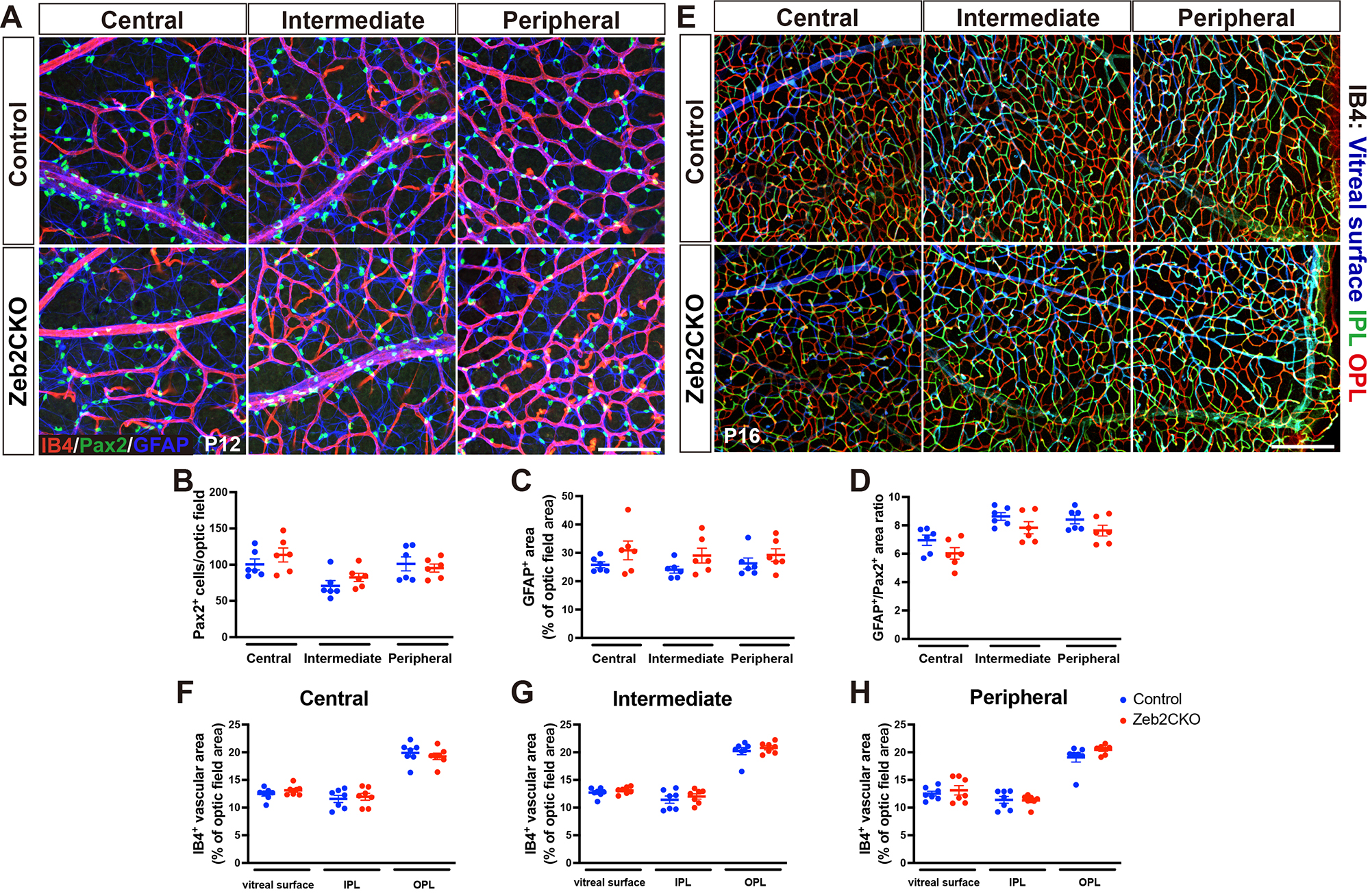


**Figure S3. The astrocytes and vasculature are normal in Zeb2CKO retinas after P12.** **(A)** P12 control and Zeb2CKO flat-mount retinas were triple-immunostained for IB4, Pax2 and GFAP. Shown are representative confocal images in the central, intermediate and peripheral regions. **(B-D)** Quantification of Pax2^+^ cells (B), GFAP^+^ area (C) and GFAP^+^/Pax2+ area ratio (D) in P12 control and Zeb2CKO retinas in the central, intermediate and peripheral regions. Data are presented as mean ± SEM (n = 6). **(E)** IB4 immunofluorescence (vitreal surface vasculature, blue; IPL vasculature, green; OPL vasculature, red) images of P16 control and Zeb2CKO flat-mount retinas in the central, intermediate and peripheral regions. **(F-H)** Quantification of IB4^+^ vascular area of P16 control and Zeb2CKO retinas in the central (F), intermediate (G) and peripheral (H) regions. Data are presented as mean ± SEM (n = 7). Abbreviations: IPL, inner plexiform layer; OPL, outer plexiform layer. Scale bar: E, 200 μm; A, 100 μm.


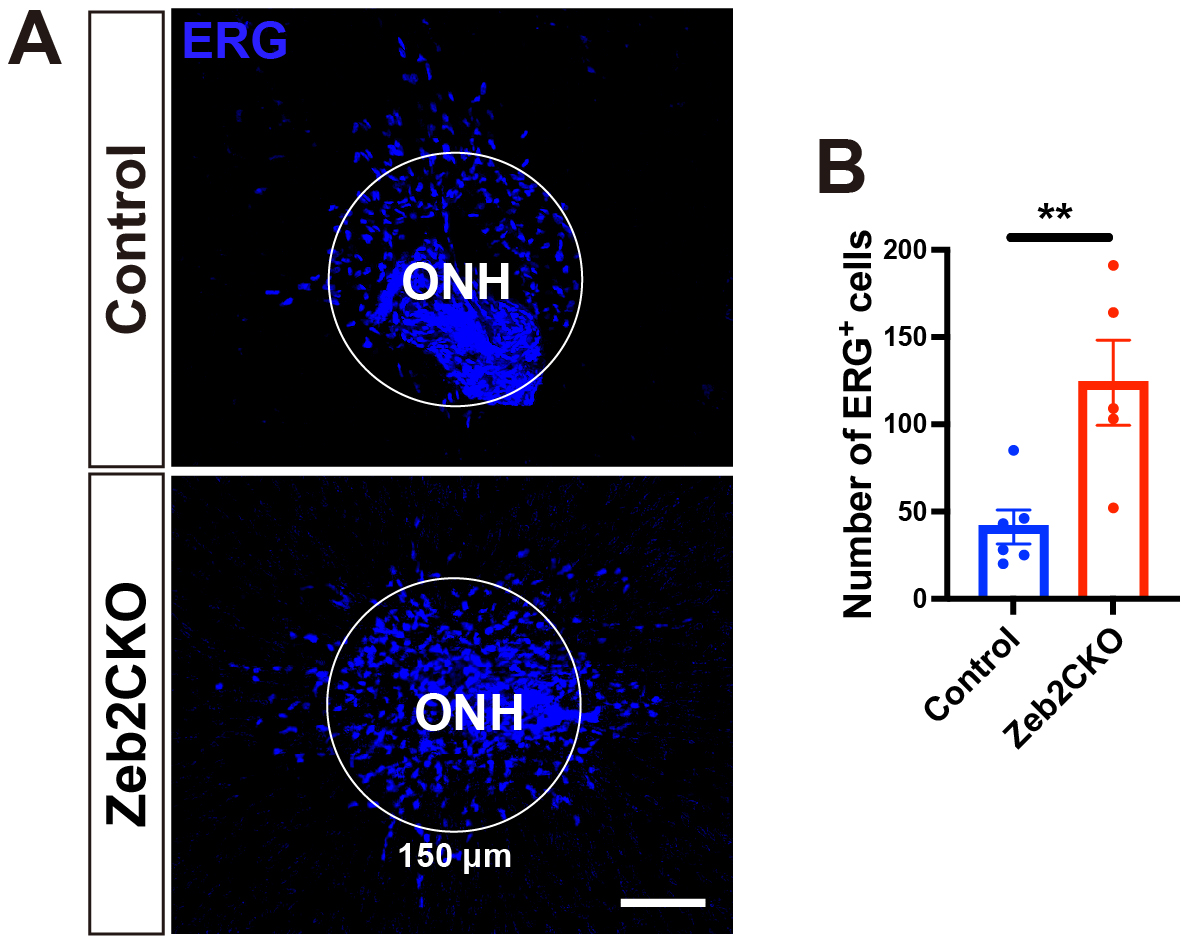


**Figure S4. Increased endothelial cells in P0 Zeb2CKO retinas.** **(A)** ERG immunofluorescence images of P0 control and Zeb2CKO flat-mount retinas around the ONH (optic nerve head) area. **(B)** Quantification of all ERG^+^ cells in the region beyond 150 μm from the ONH (denoted in A) in P0 control and Zeb2CKO retinas. Data are presented as mean ± SEM (n = 5-6). **p < 0.01. Scale bar: A, 100 μm.


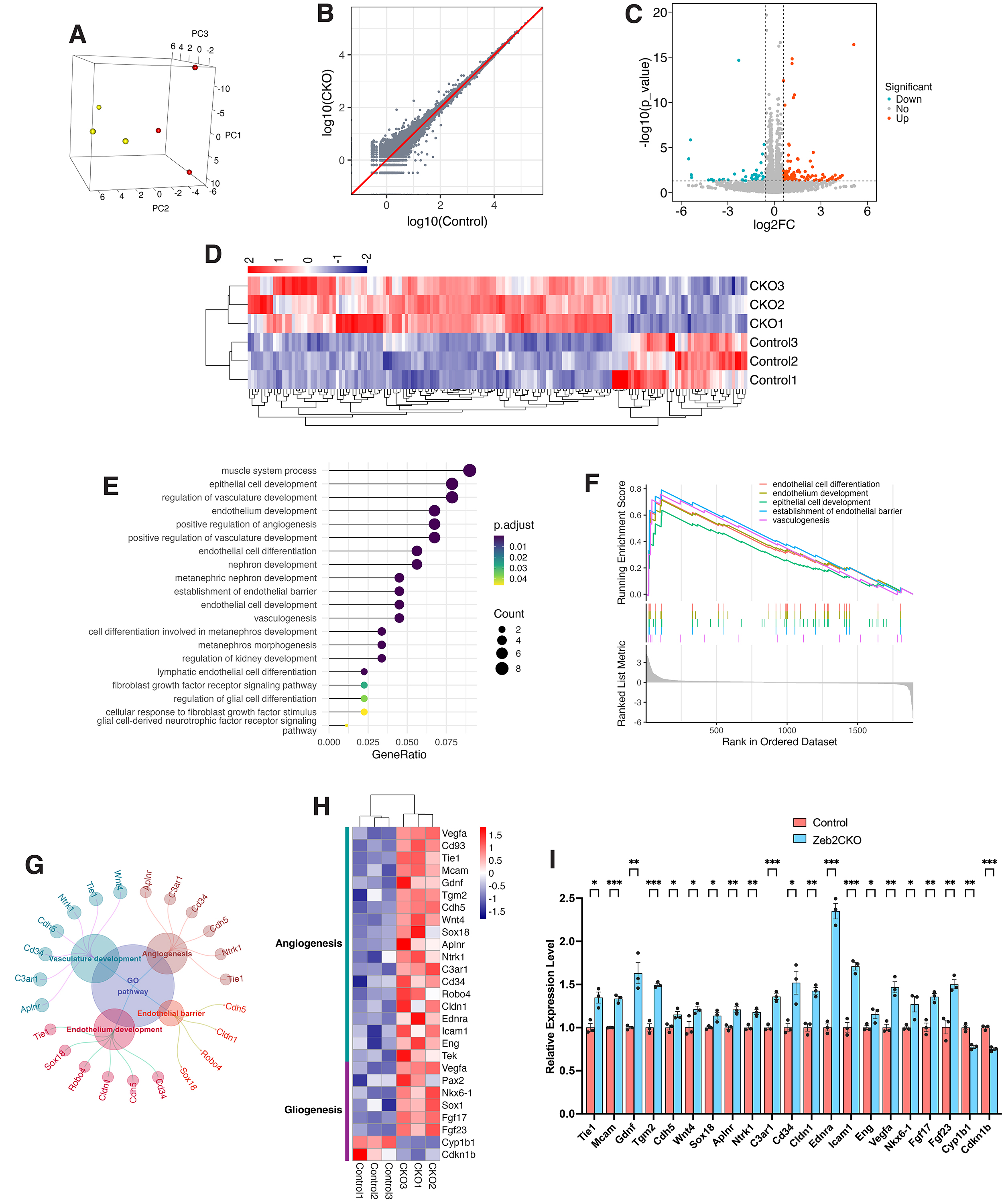


**Figure S5. Altered transcriptome profiles detected by RNA-seq analysis between E17.5 Zeb2CKO and control retinas.** (**A**) Principal component (PC) analysis of RNA-seq data showing that the Zeb2CKO retinal samples (red) are discriminated from the control samples (yellow). (**B**) Scatter plot of global gene expression profiles in CKO and control retinas. Gene expression levels are depicted in log10 scale. The diagonal line represents equal expression in the two genotypes. (**C**) Volcano plot (significance vs fold change) of significantly downregulated (green) and upregulated (red) genes (fold change ≥1.5 and p < 0.05) between the CKO and control retinas. (**D**) Heatmap of differentially expressed genes (DEGs) reveals a large group of significantly upregulated genes as well as a smaller cluster of significantly downregulated ones in CKO retinas. (**E**) Top 16 enriched GO terms plus 4 representative GO terms associated with glial cell differentiation, FGF receptor signaling, or GDNF receptor signaling for the DEGs between the CKO and control retinas. (**F**) GSEA of the RNA-seq data identifies enriched gene sets associated with endothelium development, endothelial cell differentiation, establishment of endothelial barrier, vasculogenesis, and epithelial cell development. (**G**) Network plot of 4 representative enriched angiogenesis-related GO terms or gene sets (nodes) and their associated DEGs. Node size represents the gene-set size. (**H**) Expression heatmap of a set of DEGs involved in angiogenesis, gliogenesis or FGF signaling. (**I**) qRT-PCR assay of the RNA expression levels of the indicated genes involved in angiogenesis, gliogenesis or FGF signaling in P0 control and Zeb2CKO retinas. Data are presented as mean ± SEM (n = 3). *p < 0.05; **p < 0.01; ***p < 0.001.


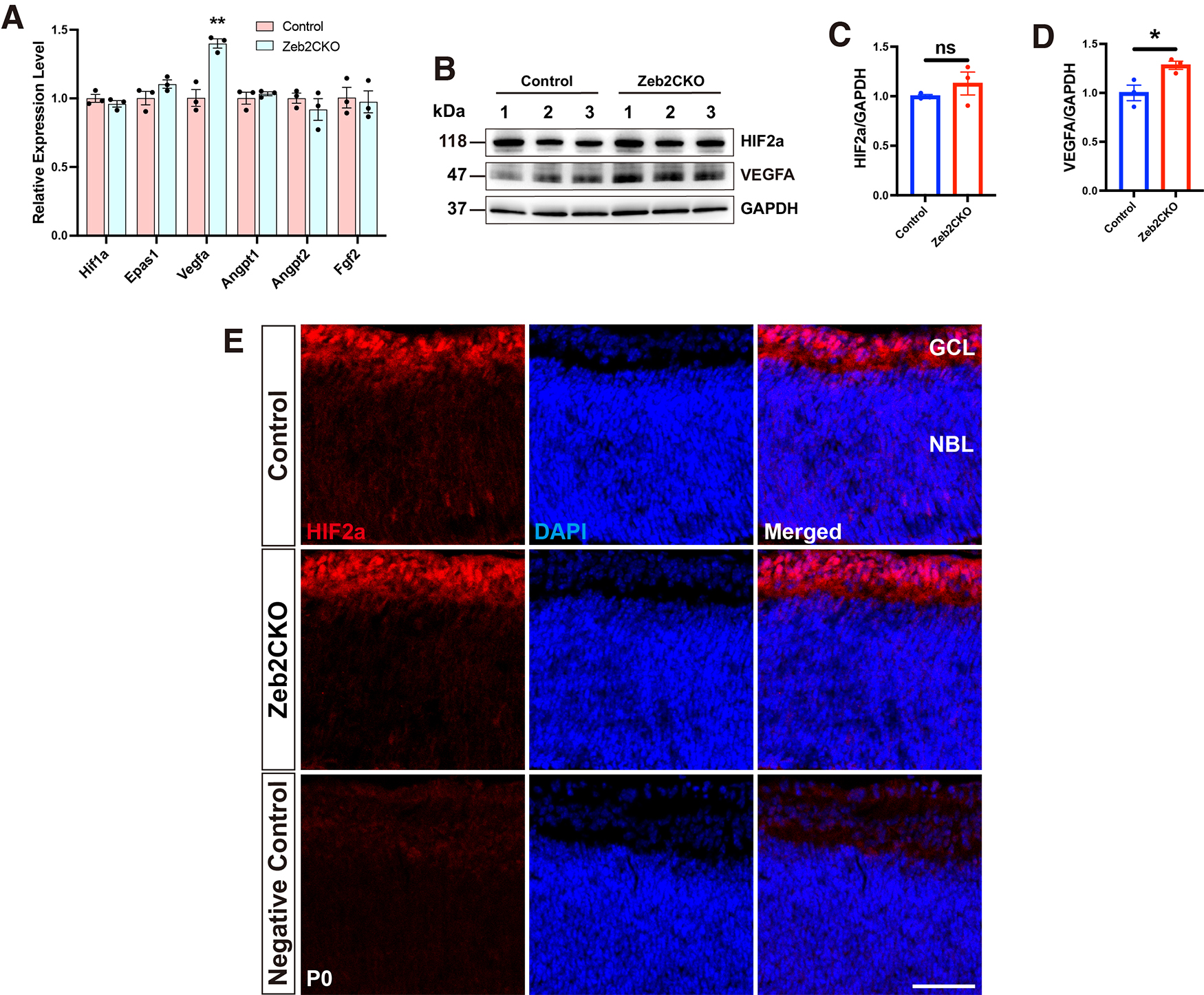


**Figure S6. Upregulation of *Vegfa* expression in P0 Zeb2CKO retinas.** **(A)** Relative RNA expression levels of *Hif1a*, *Epas1 (Hif2a), Vegfa, Angpt1, Angpt2,* and *Fgf2* were determined by qRT-PCR assay in P0 control and Zeb2CKO retinas. Data are presented as mean ± SEM (n =3). **p < 0.01. **(B)** Western blot analysis of HIF2a and VEGFA protein levels in 3 each P0 control and Zeb2CKO retinas. GAPDH served as the internal protein control. **(C,D)** Quantification of HIF2a and VEGFA protein levels in P0 control and Zeb2CKO retinas. Data are presented as mean ± SEM (n =3). *p < 0.01; ns, no significance. **(E)** Representative confocal images of HIF2a immunofluorescence and DAPI labeling of P0 control and Zeb2CKO retinal sections. Abbreviations: GCL, ganglion cell layer; NBL, neuroblastic layer. Scale bar: D, 50 μm.


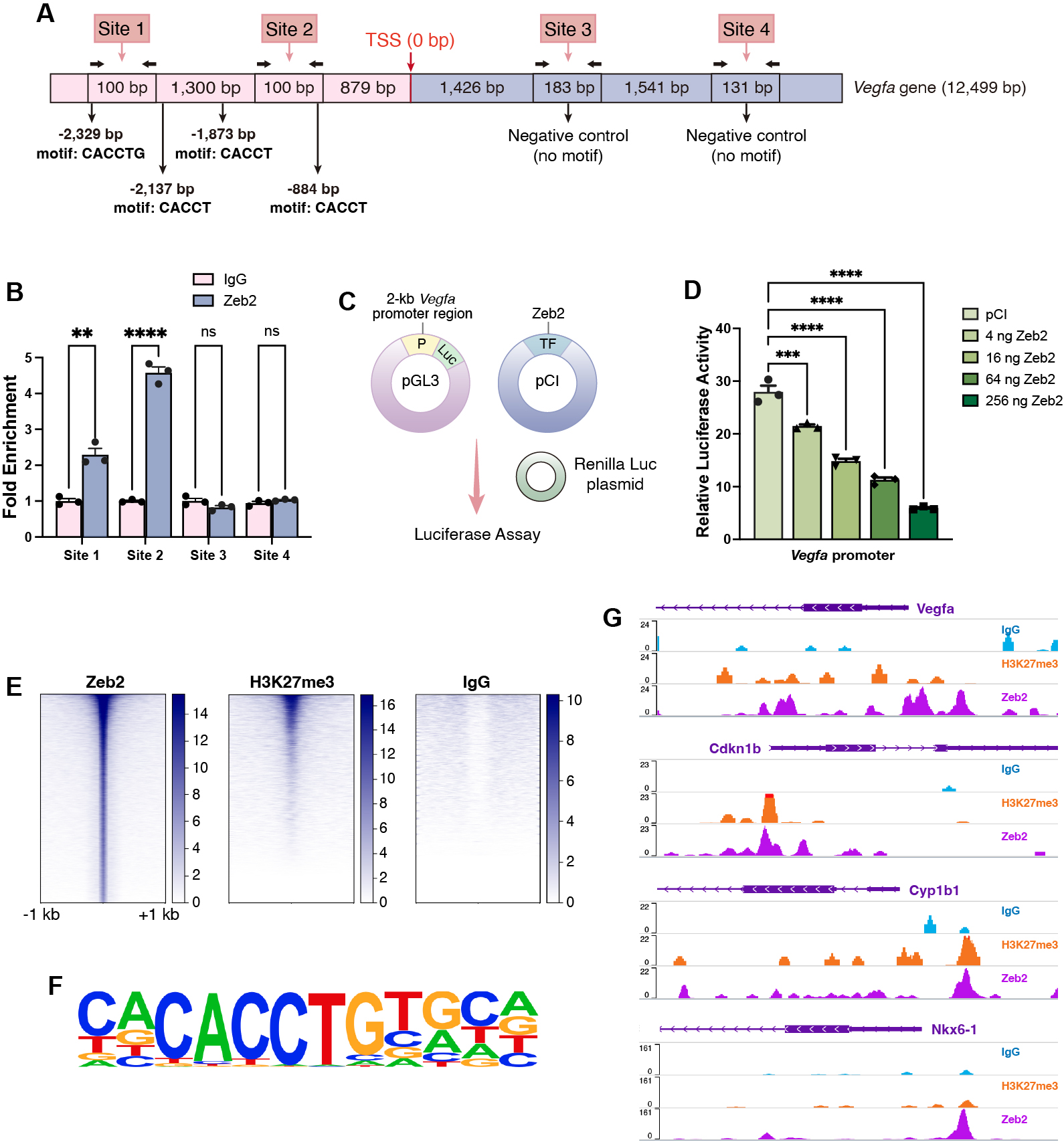


**Figure S7. Direct regulation of downstream gene expression by Zeb2.** **(A)** Schematic of the *Vegfa* promoter region with the positions of the 4 putative Zeb2 binding motifs [5’-CACCT(G)-3’] indicated. The horizontal arrows indicate the positions of PCR primers used to amplify the precipitated DNA fragments (sites 1-4) in the chromatin immunoprecipitation (ChIP) assay. The negative control fragments (sites 3 and 4) without binding motifs are located within the intron region. Indicated also is the transcription start site (TSS). **(B)** In the ChIP assay, chromatin DNA was prepared from adult mouse retinas, immunoprecipitated by an anti-Zeb2 antibody, and quantified by qRT-PCR. Data are presented as mean ± SEM (n = 3). **p < 0.01; ****p < 0.0001; ns, no significance. **(C)** Schematic of the luciferase assay. A 2-kb *Vegfa* promoter (P) fragment was inserted upstream of Luc (luciferase) in the pGL3-Basic vector. The open reading frame of the Zeb2 transcription factor (TF) gene was inserted into the pCI expression vector. **(D)** Relative luciferase activities after cotransfection of the *Vegfa* reporter plasmid with the control (pCI) plasmid or the indicated increasing amount of Zeb2 expression plasmid in 293T cells. Histograms represent the mean ± SEM of triplicate assays in a single experiment. ***p < 0.001; ****p < 0.0001. **(E)** CUT&Tag analysis of E17.5 mouse retinas were conducted to map genomic sites bound by Zeb2. Shown are heatmaps of the Zeb2 and H3K27me3 CUT&Tag signals around the Zeb2 CUT&Tag peak region. Each row represents a 1-kb region centered on the Zeb2 peak summit, sorted by Zeb2 or H3K27me3 signal enrichment. IgG serves as the negative control. **(F)** A top-ranked Zeb2-binding motif (P = 1e-67) identified by de novo motif search in a 300-bp window centered at the peak summit. **(G)** Genome browser views of Zeb2, H3K27me3, and IgG CUT&Tag signals at the *Vegfa, Cdkn1b, Cyp1b1,* and *Nkx6-1* loci. The y axis represents the number of normalized reads.


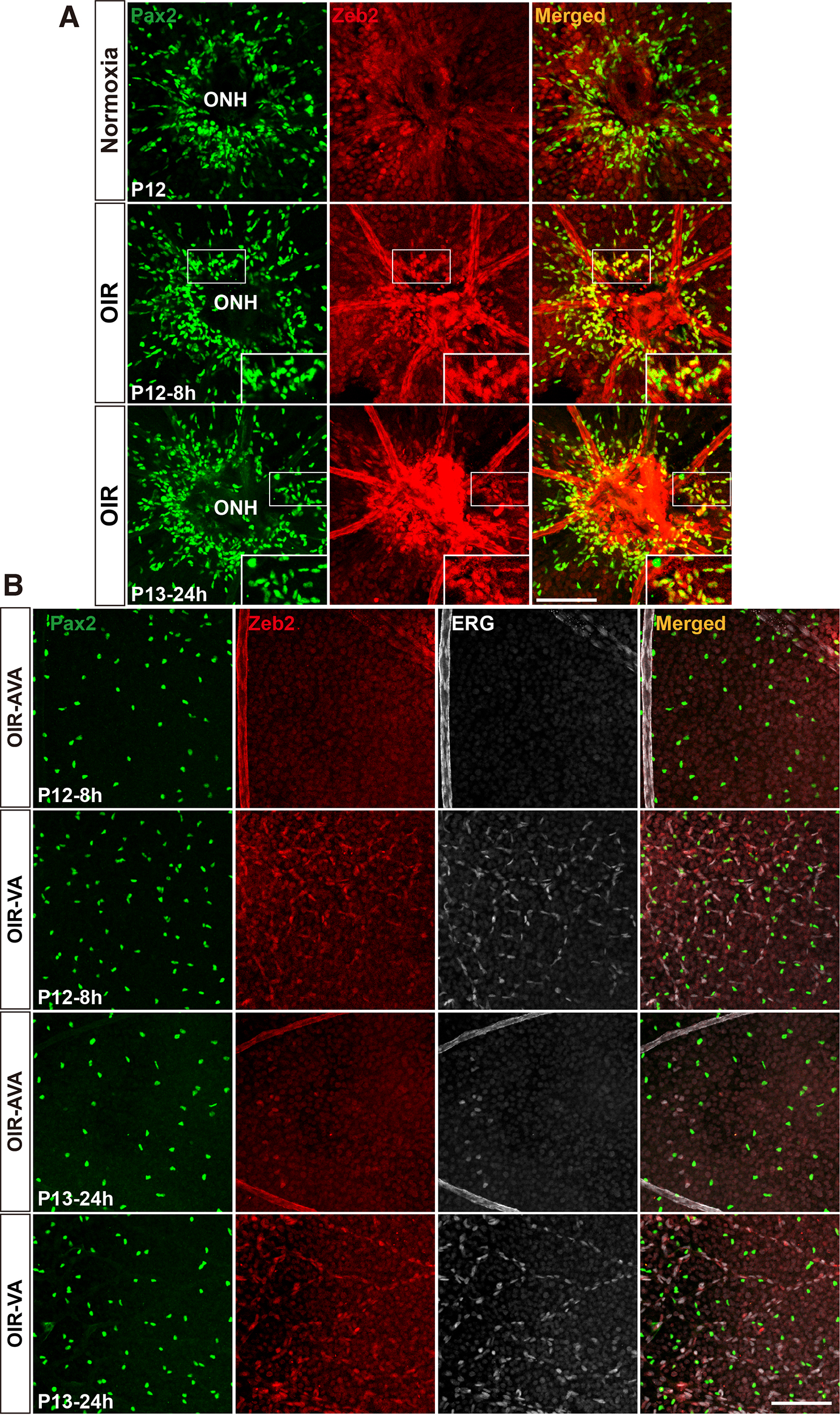


**Figure S8. Upregulation of Zeb2 expression in retinal astrocytes after hypoxic injury.** **(A)** Pax2 and Zeb2 double-immunofluorescence staining of flat-mount retinas from wild-type mice at P12 under the normoxia condition, or 8 (P12-8h) and 24 (P13-24h) hours post vaso-obliteration under the OIR condition. The insets show the corresponding outlined regions at a higher magnification. **(B)** Pax2, Zeb2 and ERG triple-immunofluorescence staining of flat-mount retinas from wild-type mice at 8 (P12-8h) and 24 (P13-24h) hours post vaso-obliteration under the OIR condition. Abbreviations: AVA, avascular area; OIR, oxygen-induced retinopathy; ONH, optic nerve head; VA, vascular area. Scale bar: A, B, 100 μm.


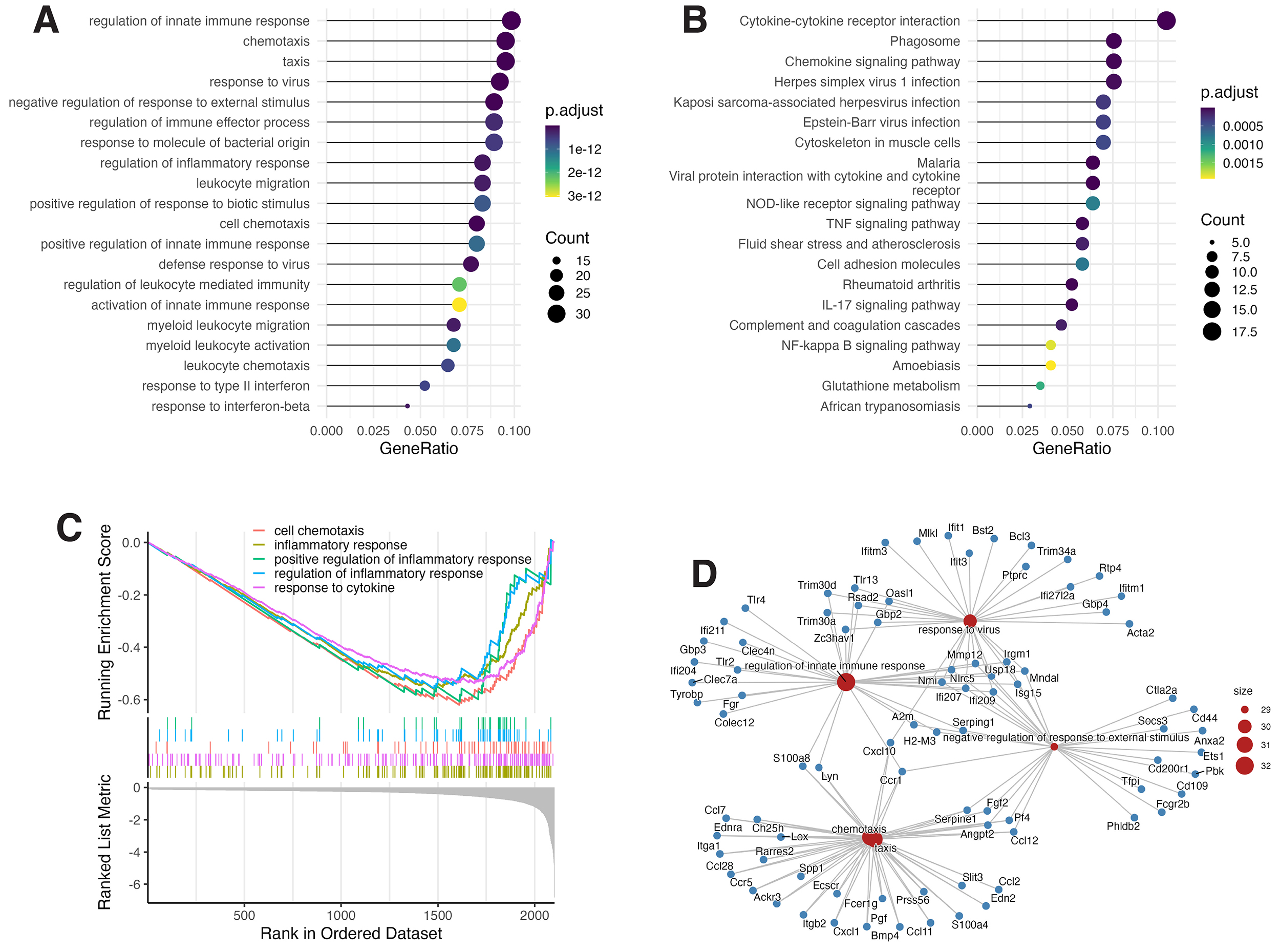


**Figure S9. GO enrichment, KEGG pathway enrichment and gene set enrichment analyses (GSEA)** **of the downregulated DEGs between P17 OIR Zeb2CKO and control retinas.** (**A,B**) Top 20 enriched GO terms (A) and KEGG pathways (B) for the downregulated DEGs. (**C**) GSEA of the downregulated genes identifies enriched gene sets associated with inflammatory response, regulation of inflammatory response, positive regulation of inflammatory response, cell chemotaxis, and response to cytokine. (**D**) Network plot of the top 5 enriched GO terms and their associated downregulated DEGs.


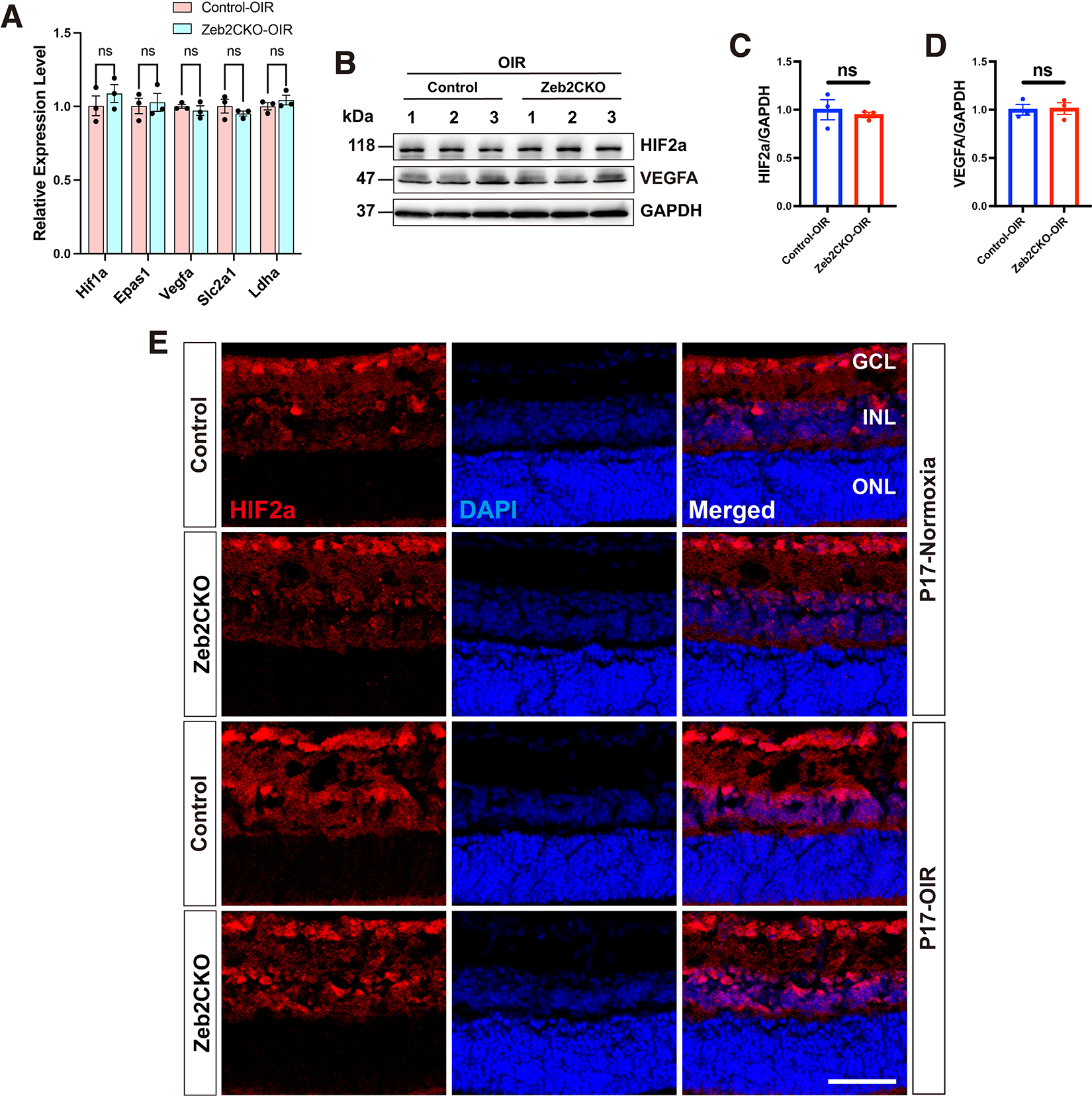


**Figure S10. Unaltered expression of HIF/VEGF pathway components in P17 Zeb2CKO retinas.** **(A)** Relative RNA expression levels of *Hif1a, Epas1(Hif2a), Vegfa,* *Slc2a1(Glut1),* and *Ldha* were determined by qRT-PCR assay in P17 OIR control and Zeb2CKO retinas. Data are presented as mean ± SEM (n =3). ns, no significance. **(B)** Western blot analysis of HIF2a and VEGFA protein levels in 3 each P17 OIR control and Zeb2CKO retinas. GAPDH served as the internal protein control. **(C,D)** Quantification of HIF2a (C) and VEGFA (D) protein levels in P17 OIR control and Zeb2CKO retinas. Data are presented as mean ± SEM (n =3). ns, no significance. **(E)** Representative confocal images of HIF2a immunofluorescence and DAPI labeling of retinal sections from P17 control and Zeb2CKO animals under the normoxic and OIR conditions. Abbreviations: GCL, ganglion cell layer; INL, inner nuclear layer; OIR, oxygen-induced retinopathy; ONL, outer nuclear layer. Scale bar: E, 50 μm.


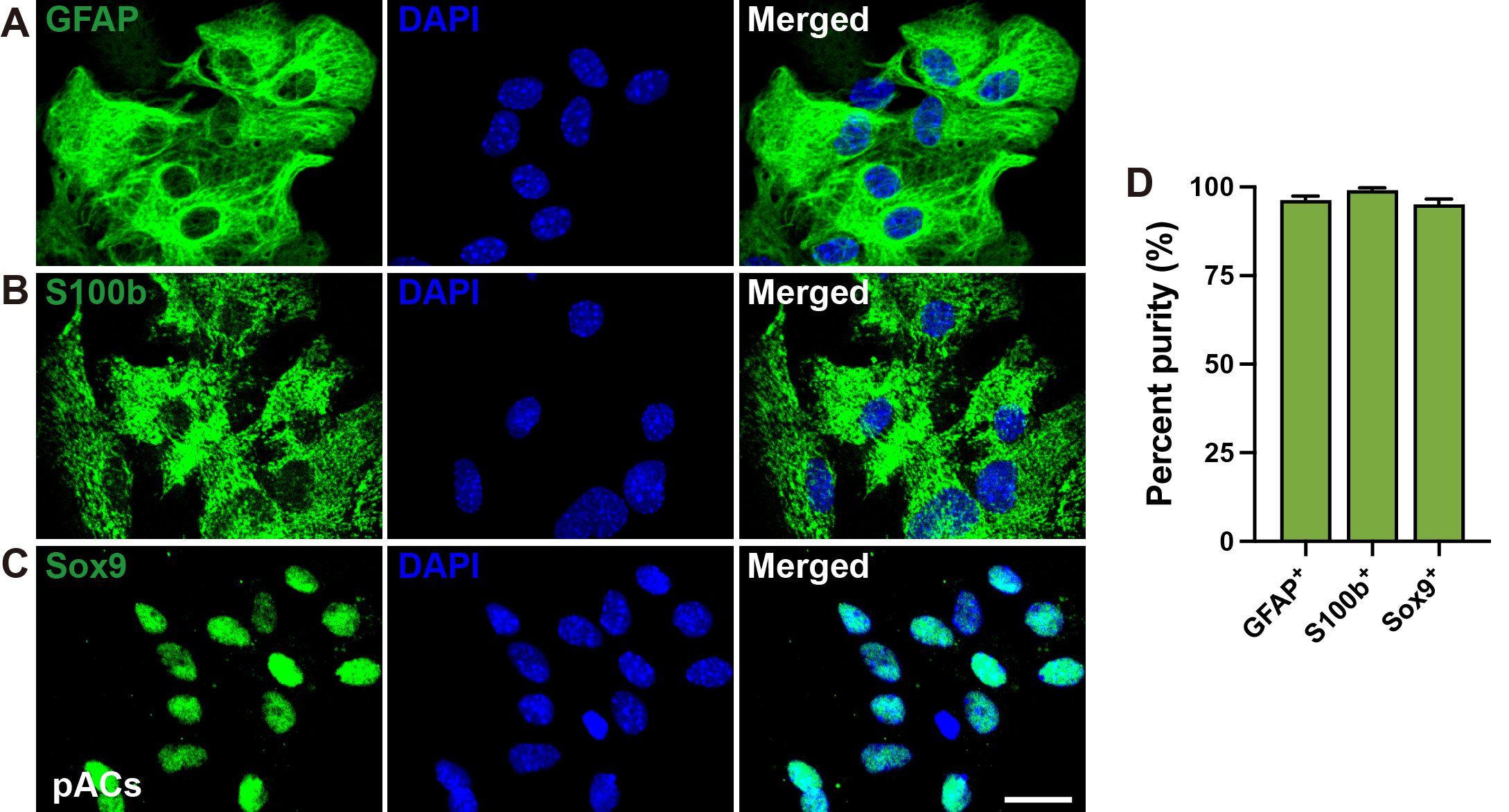


**Figure S11. Assessment of the purity of the primary astrocytes (pACs) by immunofluorescence.** **(A-C)** Representative confocal images of pACs immunostained for astrocyte-specific protein markers GFAP (A), S100b (B) or Sox9 (C) and counterstained with DAPI. pACs were isolated from P2 pups of control mice. Scale bar: 20 μm. **(D)** Quantification of the proportions of marker-positive pACs (GFAP^+^/DAPI^+^, S100b^+^/DAPI^+^ or Sox9^+^/DAPI^+^). Data are presented as mean ± SEM (n = 20).
